# Top-Down Dual-Interface Carrier Management for Highly Efficient and Stable Perovskite/Silicon Tandem Solar Cells

**DOI:** 10.1007/s40820-024-01631-x

**Published:** 2025-02-11

**Authors:** Xin Li, Zhiqin Ying, Shuo Li, Lei Chen, Meili Zhang, Linhui Liu, Xuchao Guo, Jun Wu, Yihan Sun, Chuanxiao Xiao, Yuheng Zeng, Jian Wu, Xi Yang, Jichun Ye

**Affiliations:** 1 Zhejiang Provincial Engineering Research Center of Energy Optoelectronic Materials and Devices, Ningbo Institute of Materials Technology and Engineering, Chinese Academy of Sciences, Ningbo, 315201 People’s Republic of China; 2 University of Chinese Academy of Sciences, No.19(A) Yuquan Road, Shijingshan District, Beijing, 100049 People’s Republic of China; 3 CSI Solar Technologies (JiaXing) Co.,Ltd, No.325 Kanghe Road, Gaozhao Street, Xiuzhou Jiaxing, Zhejiang, 314001 People’s Republic of China

**Keywords:** Perovskite/silicon tandem solar cells, Carrier management, Dual-interface, Post-treatment

## Abstract

Despite significant advancements in the power conversion efficiency (PCE) of perovskite/silicon tandem solar cells, improving carrier management in top cells remains challenging due to the defective dual interfaces of wide-bandgap perovskite, particularly on textured silicon surfaces. Herein, a series of halide ions (Cl^−^, Br^−^, I^−^) substituted piperazinium salts are designed and synthesized as post-treatment modifiers for perovskite surfaces. Notably, piperazinium chloride induces an asymmetric bidirectional ions distribution from the top to the bottom surface, with large piperazinium cations concentrating at the perovskite surface and small chloride anions migrating downward to accumulate at the buried interface. This results in effective dual-interface defect passivation and energy band modulation, enabling wide-bandgap (1.68 eV) perovskite solar cells to achieve a PCE of 22.3% and a record product of open-circuit voltage × fill factor (84.4% relative to the Shockley–Queisser limit). Furthermore, the device retains 91.3% of its initial efficiency after 1200 h of maximum power point tracking without encapsulation. When integrated with double-textured silicon heterojunction solar cells, a remarkable PCE of 31.5% is achieved for a 1.04 cm^2^ monolithic perovskite/silicon tandem solar cell, exhibiting excellent long-term operational stability (*T*_80_ = 755 h) without encapsulation in ambient air. This work provides a convenient strategy on dual-interface engineering for making high-efficiency and stable perovskite platforms.

**Supplementary Information:**

The online version contains supplementary material available at 10.1007/s40820-024-01631-x.

## Highlights

- An innovated top-down dual-interface carrier management strategy is developed to effectively improve both interfaces of the wide-bandgap perovskite using a multi-functionalized piperazinium chloride post-treatment.
- The 1.68 eV unencapsulated single-junction perovskite solar cells exhibit a champion PCE of 22.3%, with a record *V*_OC_ × FF product (84.4% relative to the Shockley–Queisser limit).
- An impressive PCE of 31.5% for the 1.04 cm^2^ monolithic perovskite/silicon tandem solar cell based on silicon heterojunction bottom cell is demonstrated.

**Supplementary Information:**

The online version contains supplementary material available at 10.1007/s40820-024-01631-x.

## Graphical abstract

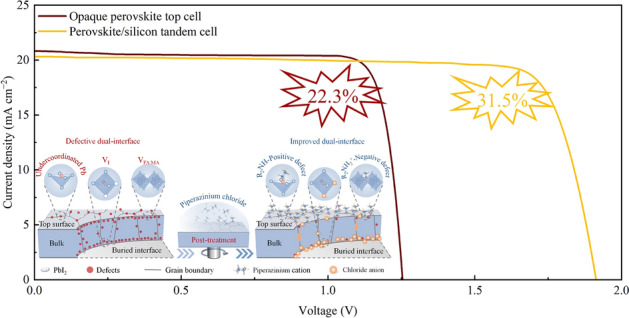

## Introduction

Recently, remarkable advancements have been made in monolithic inverted perovskite/silicon tandem solar cells (PVSK/Si TSCs), with a certified power conversion efficiency (PCE) reaching to 34.6% [1]. By comparing the achieved performance with the theoretical limit of 44.3% [2], as estimated by the Shockley–Queisser (S–Q) theory, it is evident that while the *J*_SC_ (short-circuit current density)/*J*_SQ_ (maximum possible current calculated by the S–Q limit) ratio of PVSK/Si TSCs reaches 96%, indicating near-perfect light management, there is still room for improvement in charge carrier management, as the *V*_OC_ (open-circuit voltage) × FF (fill factor) product typically reaches only around 80% or less of the S–Q limits, significantly lower than the 87% and 85% achieved by perovskite and silicon single-junction solar cells [3–5], respectively.

Previous studies suggested that the defects, in particular at film interfaces of wide-bandgap (WBG) perovskite top cells, could be recognized as the primary source to trigger the *V*_OC_ × FF losses [6–8]. To date, in PVSK/Si TSCs, most interface engineering efforts have primarily focused on passivating the defective perovskite/C_60_ top interface [9–12]. However, passivation of the buried interface, which has recently been recognized as equally crucial for device performance of perovskite single-junction solar cells [13–15], has rarely been reported [16–18]. The primary challenge lies in achieving a continuous and conformal passivation layer on the rough pyramid surface of the silicon bottom cell [19], especially for solution-processed interface materials [20, 21], without compromising carrier transport. Additionally, most interface materials are readily dissolved in the highly polar aprotic solvents typically used in perovskite precursor inks, such as *N, N*-dimethylformamide and dimethylsulfoxide [22], which diminishes the passivation effect and affects the perovskite crystallization process. Therefore, exploring a suitable and feasible method for passivating both interfaces of WBG perovskite on textured silicon surfaces is essential for effective carrier management in PVSK/Si TSCs.

In this work, an innovated top-down dual-interface carrier management strategy is developed to effectively improve both interfaces of the wide-bandgap perovskite using a multi-functionalized piperazinium chloride post-treatment. We demonstrate that piperazinium cations with large steric hindrance preferentially concentrate at the perovskite top surface, suppressing nonradiative recombination and improving energy band alignment by reducing surface defects and forming a negative surface dipole, thus enabling favorable contact between the perovskite and C_60_. Meanwhile, chloride anions with smaller ion radius spontaneously migrate downward and eventually accumulate at the buried interface, where they effectively heal defects and reduce energy offset by forming Pb–Cl bonds and inducing a *p*-type transformation of the surface energetics. As a result, the corresponding unencapsulated 1.68 eV perovskite single-junction devices exhibit a champion PCE of 22.3%, with a record *V*_OC_ × FF product (84.4% relative to the S–Q limit) and enhanced stability (91.3% retention after 1200 h of maximum power point tracking in ambient air). Furthermore, an impressive PCE of 31.5% for the 1.04 cm^2^ monolithic PVSK/Si TSCs based on silicon heterojunction bottom cell is demonstrated.

## Experimental Section

### Materials

Indium tin oxide (ITO) glass substrates (7‒9 Ohm per square, ≥ 89% transparency) were purchased from Advanced Election Technology Co., Ltd. Cesium iodide (CsI, 99.999%), dimethyl sulfoxide (DMSO, 99.8%), N, N-dimethylformamide (DMF, 99.8%), chlorobenzene (CB, 99.8%) and isopropanol (IPA, > 99.5%) were ordered from Sigma-Aldrich. Methylammonium bromide (MABr) and formamidinium iodide (FAI) were acquired from GreatCell Solar Ltd. [2-(3,6-Dimethoxy-9H-carbazol-9-yl) ethyl] phosphonic acid (MeO-2PACz), [4-(3,6dimethyl-9H-carbazol-9-yl)butyl] phosphonic acid (Me-4PACz), lead (II) bromide (PbBr_2_) and lead (II) iodide (PbI_2_) were supplied by TCI. C_60_ (99.5%) and bathocuproine (BCP, 99%) were procured from Lumtec Corp. Piperazinium iodide (PI) was purchased from Xi’an Polymer Light Technology Corp. Ethanol (anhydrous, ≥ 99.5%) was ordered from Aladdin. [2-(4-(Bis(4-methoxyphenyl)amino)phenyl)-1-cyanovinyl]phosphonic acid (MPA-CPA) was ordered from Zhoushan Huazhou Chemical Ltd. All materials were used as received.

### Devices Fabrication

#### Single-Junction Perovskite Solar Cells Fabrication

ITO substrates (2.5 × 2.5 cm^2^) were sequentially cleaned by sonication with detergent, deionized water, acetone and anhydrous ethanol for 20 min, respectively. The substrates were then dried at 85 °C for 2 h in an oven, subjected to UV-ozone treatment for 15 min and finally transferred into a N_2_-filled glovebox before use.

In this work, self-assembled monolayers (SAMs) MeO-2PACz were applied as hole-selective layers. For MeO-2PACz-based devices, SAMs were dissolved in anhydrous ethanol at a concentration of 0.3 mg mL^−1^ and stirred for 30 min before use. The prepared SAM solution was then dropped onto the ITO substrate and left for 30 s before being spin-coated at 4000 rpm for 30 s, followed by annealing at 100 °C for 10 min. Afterward, the substrates were dynamically washed with ethanol (400 μL) twice using spin coating at 4000 rpm for 50 s. The Me-4PACz and MPA-CPA were dissolved in anhydrous ethanol at a concentration of 1.0 mg mL^−1^ with the same deposition process as MeO-2PACz. For the sequential deposition of Me-4PACz and MPA-CPA, a washing process was performed for Me-4PACz while absent for MPA-CPA.

For perovskite precursor preparation, a 1.26 M perovskite precursor solution was prepared by mixing 0.06 mmol of CsI, 1.0 mmol of FAI, 0.2 mmol of MABr, 0.2 mmol of PbBr_2_ and 1.1 mmol of PbI_2_ (3% of excess) in 1 mL of a DMF: DMSO (4: 1 v/v) mixed solvent, yielding a chemical formula of Cs_0.05_(FA_0.83_MA_0.17_)_0.95_Pb(I_0.83_Br_0.17_)_3_, corresponding to a 1.63 eV perovskite. A 1.2 M perovskite precursor solution was prepared by mixing 0.204 mmol of CsI, 0.996 mmol of FAI, 0.36 mmol of PbBr_2_ and 0.84 mmol of PbI_2_ in 1 ml of DMF: DMSO (4: 1 v/v) mixed solvent, resulting in a chemical formula of Cs_0.17_FA_0.83_Pb(I_0.8_Br_0.2_)_3_, which forms a 1.65 eV MA-free perovskite. Lastly, a 1.5 M perovskite precursor solution was prepared by mixing 0.075 mmol of CsI, 1.098 mmol of FAI, 0.327 mmol of MABr, 0.354 mmol of PbBr_2_ and 1.146 mmol of PbI_2_ in 1 mL of DMF: DMSO (4: 1 v/v) mixed solvent, leading to a chemical formula of Cs_0.05_(FA_0.77_MA_0.23_)_0.95_Pb(I_0.77_Br_0.23_)_3_, which corresponds to a 1.68 eV perovskite.

For the fabrication of 1.63 eV perovskite films, the perovskite precursor was spin-coated onto the as-prepared substrates with MeO-2PACz at 3500 rpm for 35 s, during which 200 μL CB was dripped onto the center of film at 25 s before the end of spin coating. The substrates were then immediately transferred to a hotplate and annealed at 100 °C for 20 min. For the fabrication of 1.65 and 1.68 eV perovskite films, the perovskite precursor was spin-coated on the SAM-coated substrates at 3500 rpm for 40 s, with 200 μL of CB dripped onto the center of film at 15 s before the end of spin coating. The substrates were immediately transferred to a hotplate and annealed at 100 °C for 20 min. For the devices with surface treatment, PCl materials were ultrasonically dissolved in isopropanol (IPA) at different concentrations (0.1, 0.15, 0.3 and 0.5 mg mL^−1^) and dynamically spin-coated onto the cooled-down perovskite films at 6,000 rpm for 30 s, followed by annealing at 100 °C for 10 min. The PBr and PI surface treatments involved preparing precursor solutions by dissolving 0.20 mg of PBr and 0.26 mg of PI in 1 mL of IPA, respectively. The spin coating process for PBr and PI was similar to that of PCl.

Finally, 20 nm of C_60_ (0.3 Å s^−1^), 6 nm of BCP (1.0 Å s^−1^) and 100 nm silver (Ag) electrode (1.0 Å s^−1^) were thermally evaporated under high vacuum (< 5 × 10^−4^ Pa) to obtain opaque perovskite solar cells. The device area was defined and characterized as 0.1 cm^2^ by a metal shadow mask.

#### Monolithic Perovskite/Silicon Tandem Solar Cells Fabrication

The front-junction silicon bottom cells were fabricated using n-type Czochralski (CZ) monocrystalline Si wafers with a thickness of approximately 150 μm and a resistivity of 2 Ω cm. A double-side random-pyramid texture was achieved using an aqueous KOH solution, resulting in an average pyramid height distribution of less than 1 μm on both sides. Following standard RCA cleaning, the wafers were immersed in buffered HF solution to remove the thin chemical oxide layer from the surface. On the front side, intrinsic hydrogenated amorphous silicon (a-Si:H) and phosphorus-doped hydrogenated microcrystal silicon (μc-Si:H(*n*)) layers were deposited using a plasma-enhanced chemical vapor deposition (PECVD) system for dangling bond passivation and electron selection, respectively. Subsequently, intrinsic a-Si:H and boron-doped μc-Si:H (μc-Si:H(*p*)) layers were deposited on the rear side using the same system. A stack of ITO and Ag was deposited on the surface of μc-Si:H(*p*) through a shadow mask, resulting in a contact area 1.3 × 1.3 cm^2^, slightly larger than the active area of the completed tandem cell (1.1 × 1.1 cm^2^). The front side was finalized by sputtering a 10-nm-thick ITO layer through the same shadow mask. The wafers were finally cut down to 2.5 × 2.5 cm^2^ substrates and annealed for 10 min at 200 °C in ambient air to recover the sputtering damage. For perovskite top cells, the substrates were UV-ozone treated for 15 min. Then, the NiO_*X*_ layer was RF-sputtered onto the pre-cleaned substrates at room temperature with a chamber pressure of 0.40 Pa, RF power of 90 W, argon flow rate of 20 sccm for 3 min without further annealing and UV-ozone treatment. Subsequently, the NiO_*X*_ substrates were directly transferred into the N_2_ glove box for the subsequential spin coating process. The deposition processes of hole-selective layer MeO-2PACz, perovskite film, electron selective layer C_60_ and hole blocking layer BCP were the same as that of the single-junction perovskite solar cells. After the deposition of BCP, 1 nm of thin Ag was thermally deposited on the top of BCP to form a BCP: Ag buffer layer, after which an indium zinc oxide (IZO) transparent electrode (100 nm, 60 Ohm per square, 1.3 × 1.3 cm^2^ defined by a shadow mask) was deposited by radio frequency (RF) magnetron sputtering at room temperature with a RF power of 80 W. An Ag finger with a thickness of 500 nm was thermally evaporated using a high-precision shadow mask with a deposition rate of 1.5 Å s^−1^. The finger width is 50 µm (with 2.5 mm spacing in between subsequent fingers) and the busbar that surrounds the active area is 1.25 mm. To protect the samples from damaging by needle probes in subsequent device measurements, a low-temperature Ag paste was applied to a pad area (of about 5 mm^2^) by a handheld brush, and the samples were annealed at 85 °C for 5 min in a drying oven. Finally, a 100-nm-thick MgF_x_ layer was full-area evaporated on the samples by an e-beam system as an antireflection layer with a deposition rate of 1 Å s^−1^. The thicknesses of the C_60_, BCP, IZO and MgF_x_ layers were calibrated by spectroscopic ellipsometry. The deposition rate and thickness of each experiment were monitored by quartz-crystal microbalance sensors. All thermal evaporation processes were performed on a thermal evaporation system (Beijing Technol Science Co., Ltd.).

### Characterization

#### Material Characterization

Nuclear magnetic resonance (NMR) measurements including ^1^H-NMR spectra were performed using an AVANCE NEO 600 (Bruker, Switzerland) with DMSO-d_6_ as the locking solvent. The ^13^C-NMR spectra were recorded in DMSO-d_6_ using a Bruker Avance III-400 MHz NMR spectrometer. Fourier transform infrared spectroscopy (FTIR) was conducted using a THEMOR-NICOLET 6700.

#### Film Characterization

The top-view and cross-section images of perovskite films and perovskite/Si tandems were characterized uisng a Hitachi S-4800 FEG scanning electron microscope (SEM) at an accelerating voltage of 4.0 kV. General X-ray diffraction (XRD) and grazing incidence XRD patterns of perovskite films were recorded using a D8 ADVANCE DAVINCI (Bruker, German) equipped with Cu K*α*_1_ irradiation of *λ* = 1.5418 Å. Depth-resolved grazing incident XRD (GIXRD) was performed with a Rigaku Smart Lab five-axis X-ray diffractometer, utilizing Cu K*α* radiation (*λ* = 1.54050 Å) at 45 kV and 200 mA to obtain residual stresses within the perovskite films. Notably, a slow scan rate of 0.15° min^−1^ was employed to ensure fine structural information for the residual stress test. UV–Vis absorption spectra of perovskite films and toluene solvent were obtained uisng a Perkin-Elmer Lambda 950 UV–Vis spectrophotometer, with a measured wavelength range from 300 to 850 nm. Time-resolved photoluminescence (TRPL) were conducted using a HORIBA FL3-111 spectrometer with 450 nm excitation. X-ray photoelectron spectroscopy (XPS) and ultraviolet photoelectron spectroscopy (UPS) spectra were obtained using a multifunctional photoelectron spectrometer (Axis Ultra DLD, Kratos) under ultrahigh vacuum (3.0 × 10^−8^ Torr) with nonmonochromatic He-I excitation (21.22 eV). XPS mapping measurements were conducted on the Thermo ESCALAB 250Xi with the Al Kα radiation (1486.6 eV, 150 W), and the signal collection area for each sample was 1 × 1 mm^2^. The XPS spectra were calibrated using the binding energy of 284.8 eV for C 1s. Time-of-flight secondary-ion mass spectrometry (ToF–SIMS) was conducted with a PHI nano-TOF II TOF–SIMS (ULVAC-PHI, Japan) system. During data acquisition, a pulsed 30 keV Bi_3_^++^ LMIG primary ion beam was utilized to generate secondary ions. All resultant ToF–SIMS spectra were obtained from an 80 × 80 μm^2^ area on a Glass/ITO/MeO-2PACz/perovskite/PX (*X* = Cl, Br, I) stack. The root mean square (RMS) surface roughness of the perovskite films was measured using a Dimension 3100 (Vecco, USA). KPFM measurements were conducted with the Park NX20 system under contact mode. The surface distribution of PCl, PBr and PI ions on the perovskite films was characterized with a NanoIR3-FS Atomic Force Microscope (NanoIR3-FS AFM). Chemical images were obtained using a Contact Mode AFM Probe for nanoIR3 in ambient atmosphere. For photoluminescence quantum yield (PLQY) measurements, three configurations were considered: (1) quartz glass with perovskite directly deposited onto this substrate; (2) glass/ITO/SAMs/perovskite/PX (*X* = Cl, Br, I) half-stack devices; (3) glass/ITO/SAMs/perovskite/PX (*X* = Cl, Br, I)/C_60_ full-stack devices. Excitation for the PLQY measurements was performed with a 520 nm CW laser (Class 3b) through an optical fiber into an integrating sphere. The laser intensity was adjusted to a 1-sun equivalent intensity by illuminating 2.5 × 2.5 cm^2^-size perovskite films under short-circuit conditions and matching the current density to the *J*_SC_ under the sun simulator (20.8 mA cm^−2^ at 100 mW cm^−2^). A second optical fiber was used to connect the output of the integrating sphere to a spectrometer equipped with a silicon CCD camera. The system was calibrated using a calibrated halogen lamp with specified spectral irradiance, which was directed into the integrating sphere. A spectral correction factor was established to match the spectral output of the detector to the calibrated spectral irradiance of the lamp. The spectral photon density was obtained from the corrected detector signal (spectral irradiance) by division through the photon energy (ℎv), and the photon numbers for excitation and emission were derived from numerical integration using MATLAB.

#### Device Characterization

The current density versus voltage (*J‒V*) curves and the dependence of open-circuit voltage (*V*_OC_) on light intensity were measured using an Enlitech SS-F5-3A solar simulator under 1-sun conditions (100 mW cm^−2^, AM 1.5G). The *J‒V* curves of the perovskite single-junction solar cells and perovskite/Si tandems were recorded in air. The voltage range for the forward and reverse scans of perovskite single-junction devcies was maintained between − 0.1 → 1.28 V and 1.28 →  − 0.1 V, respectively, and for perovskite/Si tandem solar cells, it was maintained between − 0.1 → 1.95 V and 1.95 →  − 0.1 V, respectively. All *J‒V* measurements were conducted at a scan rate of 10 mV s^−1^. The aperture areas of the small- and large-area shade masks are 0.1 and 1.04 cm^2^, respectively, while the active area of the tandems for the large area was 1.1 × 1.1 cm^2^. External quantum efficiency (EQE) measurements were conducted using a QE measurement system (QE-R, EnliTech), with light intensity at each wavelength calibrated using certified reference cells (Si and Ge reference solar cells) for the 300‒1200 nm wavelength range. For perovskite/Si tandem solar cells, the perovskite top cell was measured while saturating the Si bottom cell with continuous biased light from a white light source equipped with a long-pass (> 850 nm) filter. To maintain short-circuit conditions, a bias voltage of ~ 0.3 V was applied during the measurement. Conversely, the Si bottom cell was measured while saturating the perovskite top cell with continuous biased light from a white light source equipped with a low-pass (< 550 nm) filter. All EQE characterizations were performed at ambient air and without encapsulation. Electrochemical measurements, including capacitance versus frequency (*C*–*F*) curves, trap density of states (t-DOS) curves and interface defect density (*N*_SS_), were obtained using a 1240A impedance analyzer. Transient photovoltage (TPV) and transient photocurrent (TPC) spectra were measured with a TranPVC W900 system (oriental spectra technology (GuangZhou) CO. Ltd.), where the cell was excited by a 517 nm pulse laser (NanoQ, 18 ns, 4 nm). Mott-Schottky plots were measured on an electrochemical workstation (CH1660E). Grazing incident wide-angle X-ray scattering (GIWAXS) measurements were performed with a Xeuss 2.0 SAXS/WAXS laboratory beamline using a Cu X-ray source (8.05 keV, 1.54 Å) and a Pilatus3R 300 K detector, with an incidence angle of 0.2°. Electroluminescence (EL) spectra and the external quantum efficiency of electroluminescence (EQE_EL_) for perovskite solar cells were recorded using a commercialized system (XPQY-EQE, Guangzhou Xi Pu Optoelectronics Technology Co., Ltd.) equipped with an integrated sphere and a photodetector array.

The long-term operational stability of the perovksite single-junction solar cells was assessed following ISOS-L-1 procedures. The measured devices were unencapsulated, and no cooling was implemented under constant illumination. Measurements were carried out under ambient conditions at temperatures ranging from 25 to 35 °C, with relative humidity of 40%‒60% (measured using a calibrated humidity tracker). The devices were monitored at the maximum power point (MPP) using a solar cell aging test system (91 PVKSOLAR) with a light-emitting diode (LED) simulating the AM 1.5G spectrum. During the MPPT test, the *J‒V* curves of the devices were obtained every 5 h to determine the appropriate loads for the MPPT. For the long-term operational stability of monolithic perovskite/silicon tandem solar cells, the light-emitting diode was replaced with a xenon lamp while other test conditions remained unchanged.

## Results and Discussion

### Top-Down Dual-Interface Distribution of Piperazinium Chloride

In this work, the triple-cation n-type perovskite Cs_0.05_(FA_0.77_MA_0.23_)_0.95_Pb(I_0.77_Br_0.23_)_3_ absorber prepared using a one-step deposition method was employed [23] and the self-assembled monolayer (SAM) of [2-(3,6-dimethoxy-9H-carbazol9-yl) ethyl] phosphonic acid (MeO-2PACz) was selected as the hole-selective contact. We initiated our investigation by optimizing the concentration of piperazinium chloride (PCl). As shown in Fig. S1 and Table S1, the optimal concentration for PCl was determined to be 0.15 mg mL^−1^ (1.2 mmol mL^−1^). Notably, piperazinium bromide (PBr) and piperazinium iodide (PI) were also introduced for parallel studies. The detailed synthesis procedures are available in Figs. S2 and S3. The concentrations of PBr and PI were set to approximately 0.20 and 0.26 mg mL^−1^, respectively, closely aligning with the optimal molar concentration of PCl. Unless otherwise stated, subsequent characterizations were carried out based on these optimized concentrations.

**Fig. 1:**
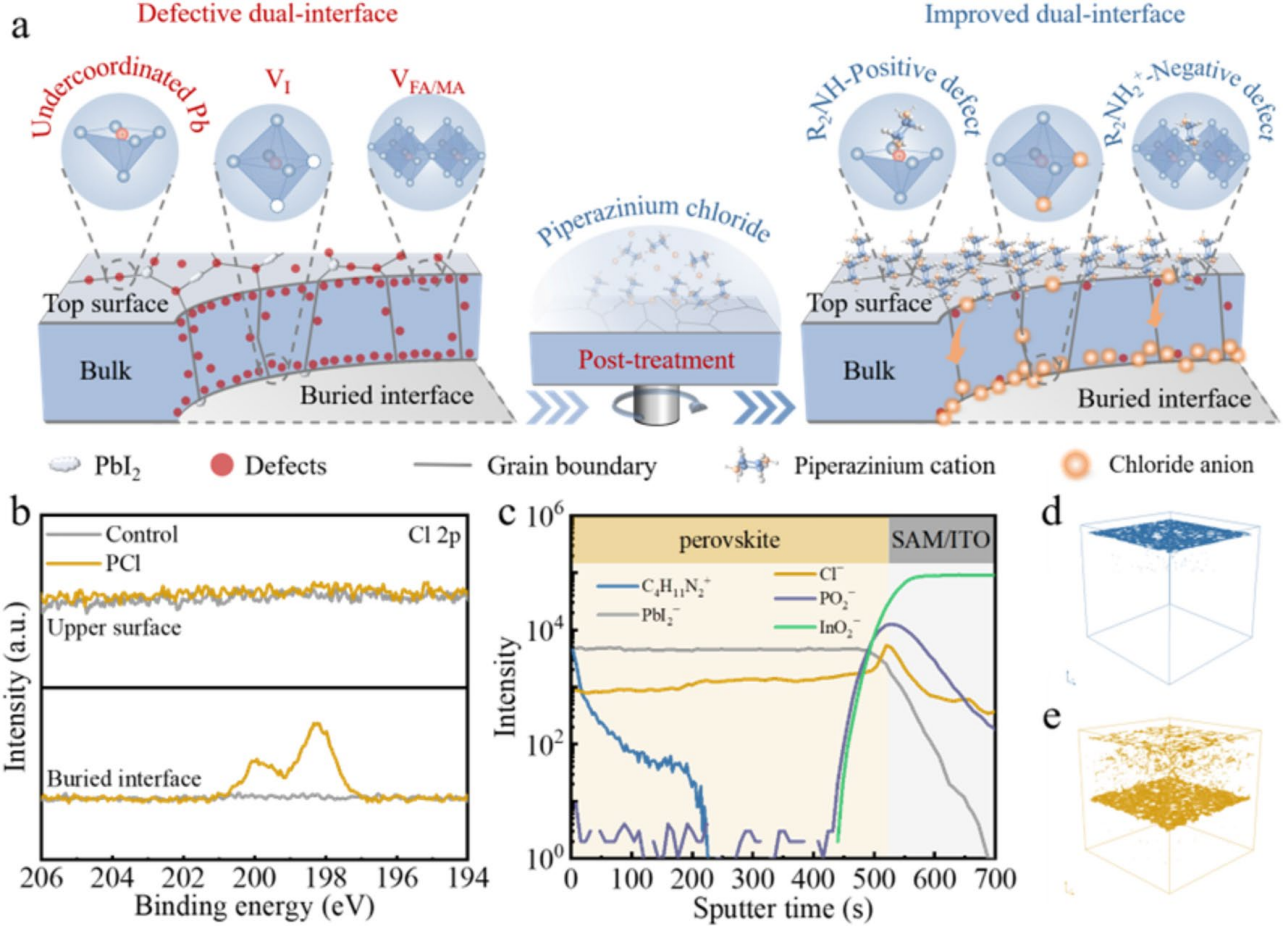
**a** Schematic illustration demonstrating the spatial distribution of piperazinium cation and chloride anion after PCl post-treatment. **b** Cl 2*p* XPS spectra of the PCl perovskite film. **c** ToF–SIMS depth profile for the PCl perovskite film. C_4_H_11_N_2_^+^ is piperazinium cation. **d** ToF–SIMS 3D reconstructed image of piperazinium cations. **e** ToF–SIMS 3D reconstructed image of chloride anions

As illustrated in Fig. 1a, the pristine perovskite film without post-treatment exhibits defective dual-interfaces, including undercoordinated Pb ions, iodine vacancy (V_I_) and A-site vacancy (V_FA/MA_) defects, etc., giving rise to significant nonradiative recombination. Upon PCl post-treatment, piperazinium cations tend to enrich on the perovskite top surface due to their large steric hindrance [24, 25]. Since the piperazinium cation contains both electron donor (R_2_NH) and electron acceptor (R_2_NH_2_^+^) functional groups within the same six-membered ring, both negative and positive perovskite surface traps can be passivated, thereby reducing nonradiative recombination. Additionally, the surface work function of the perovskite can be altered by the molecular dipole (Fig. S4), improving surface energy band alignment [26, 27]. In contrast, Cl^−^ ions, with smaller ion radius, spontaneously migrate into the perovskite bulk and end up enriching at the buried interface, in turn healing buried defects and reducing energy offset [28, 29]. Consequently, a top-down carrier management strategy for improving both interfaces of the perovskite is achieved.

To verify our hypothesis, we first conducted high-resolution X-ray photoelectron spectroscopy (XPS) measurements to explore the element profiles of the perovskite upper and bottom surfaces before and after surface treatments. The bottom surface of the perovskite film was exposed by using UV-curable glue to peel off the perovskite layer from the indium tin oxide (ITO)/MeO-2PACz substrate as shown in Fig. S5 [30]. For the top surface, a new peak appearing at 285.4 eV in the C 1s spectra are observed after PCl, PBr and PI treatments, which is attributed to the C–NH–C bond of piperazinium cation [31] (Fig. S6). Turning to the N 1s spectra, we notice an obvious increase in the higher binding energy peak at 402.2 eV, likely resulting from the characteristic C–NH–C bond [24, 32, 33], consistent with the N 1s spectra of the pure piperazinium halide powders (Fig. S7). These results confirm the presence of the piperazinium cation on the perovskite top surface. In contrast, no visible signals associated with the piperazinium cation are observed on the perovskite bottom surfaces after surface treatments (Fig. S8), indicating that piperazinium cations may not diffuse to the perovskite buried interface, but concentrate at the perovskite top surface. Furthermore, we examined the anion profiles of the perovskite upper and bottom surfaces before and after surface treatments (Figs. 1b and S9). After PBr and PI treatments, an obvious increase in signals across the Br 3*d* and I 3*d* environments on the perovskite top surface is detected, indicating the successful incorporation of Br in PBr and I in PI on the perovskite top surface (Fig. S9). However, no significant increase in Br and I signals at the buried interfaces is probed in the PBr and PI films, suggesting that the incorporated Br and I ions mainly congregate near the perovskite top surfaces without penetrating to the perovskite bottom surfaces [34]. Surprisingly, no visible Cl signals are found on the top surface in the PCl case from the Cl 2*p* XPS spectra, but apparent Cl peaks are detected at the buried interface (Fig. 1b). Similar trends can also be observed from the XPS mapping images of Cl 2*p* (Fig. S10). These results imply that Cl ions prefer to diffuse through the perovskite bulk and concentrate at the buried interface as compared to Br and I ions regardless of the substrates (Fig. S11), forming a chloride-rich region, thus improving the buried interface and enabling more effective carrier management.

To further investigate the spatial distributions of piperazinium cations and halide anions across the film thickness, time-of-flight secondary-ion mass spectrometry (ToF–SIMS) measurements were performed. The characteristic ionic species and corresponding depth profiles of PCl-, PBr- and PI-treated perovskite films are shown in Figs. 1c and S12. As expected, piperazinium cations exhibit a gradient distribution, with an intense signal at the top surface that drop sharply as the probe deepens (Fig. 1c), suggesting that piperazinium cations are mainly enriched on the top surface of the perovskite layers, agreeing well with the above XPS results. Regarding the anions, both Br^−^ and I^−^ ions exhibited limited diffusion into the perovskite films, primarily enriching near the top surface (Fig. S12), in line with previous reports [28, 35, 36]. Strikingly, the signal of Cl^−^ ions is found to primarily concentrate at the buried interface, with a small number of Cl^−^ ions distributed at the top surface and bulk of the perovskite film (Fig. 1c), reflecting the XPS results. The discrepancy in the spatial distribution between halide anions may be attributed to the following three aspects. First, halogen vacancy filling or solid-state ion exchange of Cl^−^ (or Br^−^) with I^−^ in the [PbI_6_]^4−^ octahedron or PbI_2_ is likely to occur after post-treatments [37, 38], as verified by the ^1^H nuclear magnetic resonance (NMR) spectra (Fig. S13). The solid-state ion exchange reaction of Cl/I is found to be much stronger than that of Br/I, implying that more Cl^−^ can adsorb on the perovskite surface under the same molar concentration, providing an abundant Cl^−^ source replenishment. Second, it has been revealed that absorbed Cl^−^ on the perovskite surface spontaneously diffuses into the perovskite bulk without any external field [28, 39], whereas absorbed Br^−^ and I^−^ preferentially remain on the perovskite surface or migrate driven by annealing [28, 36]. This suggests that it is thermodynamically favorable for Cl^−^ ions to migrate toward the buried interface, minimizing the free energy of the film system. Third, Cl^−^ possesses the smallest ion radius among the candidate PX species, resulting in the lowest activation energy barrier [40], making it much easier for Cl^−^ to diffuse from the perovskite surface to the bulk until reaching the buried interface.

We further illustrate corresponding three-dimension (3D) reconstructed images of piperazinium cations and Cl^−^ ions to vividly observe their spatial distributions. The 3D image demonstrates that the piperazinium cations introduced by post-treatment unambiguously concentrate at the perovskite top surface (Fig. 1d), likely attributed to the strong adhesion of piperazinium cations to the perovskite surface [24]. Notably, the piperazinium cations exhibit relatively uneven surface coverage, which may attribute to the nonuniform distribution of surface defects (with some defect-free regions) causing a preferential coordination of piperazinium cations with the surface defect sites, thus leading to incomplete occupation on the perovskite surface [41, 42]. The selective distribution of piperazinium cations on the perovskite top surface is further verified by the atomic force microscopy-based infrared spectroscopy measurements (Figs. S14 and S15). In striking contrast, Cl^−^ ions diffuse into the perovskite bulk upon surface treatment and predominantly accumulate at the interface between the perovskite and the hole transport layer (HTL), allowing to act as passivators of interface defects and regulators of surface energetics. These findings collectively confirm the spatially bidirectional distribution of piperazinium cations and chloride anions upon PCl post-treatment, demonstrating a top-down carrier management characteristic, which is expected to provide comprehensive defect passivation and surface energetics regulation at both interfaces, thus leading to better performance than that achieved with PBr and PI treatments.

### Improved Morphology, Crystal Quality and Surface Chemistry of Dual-Interfaces

**Fig. 2:**
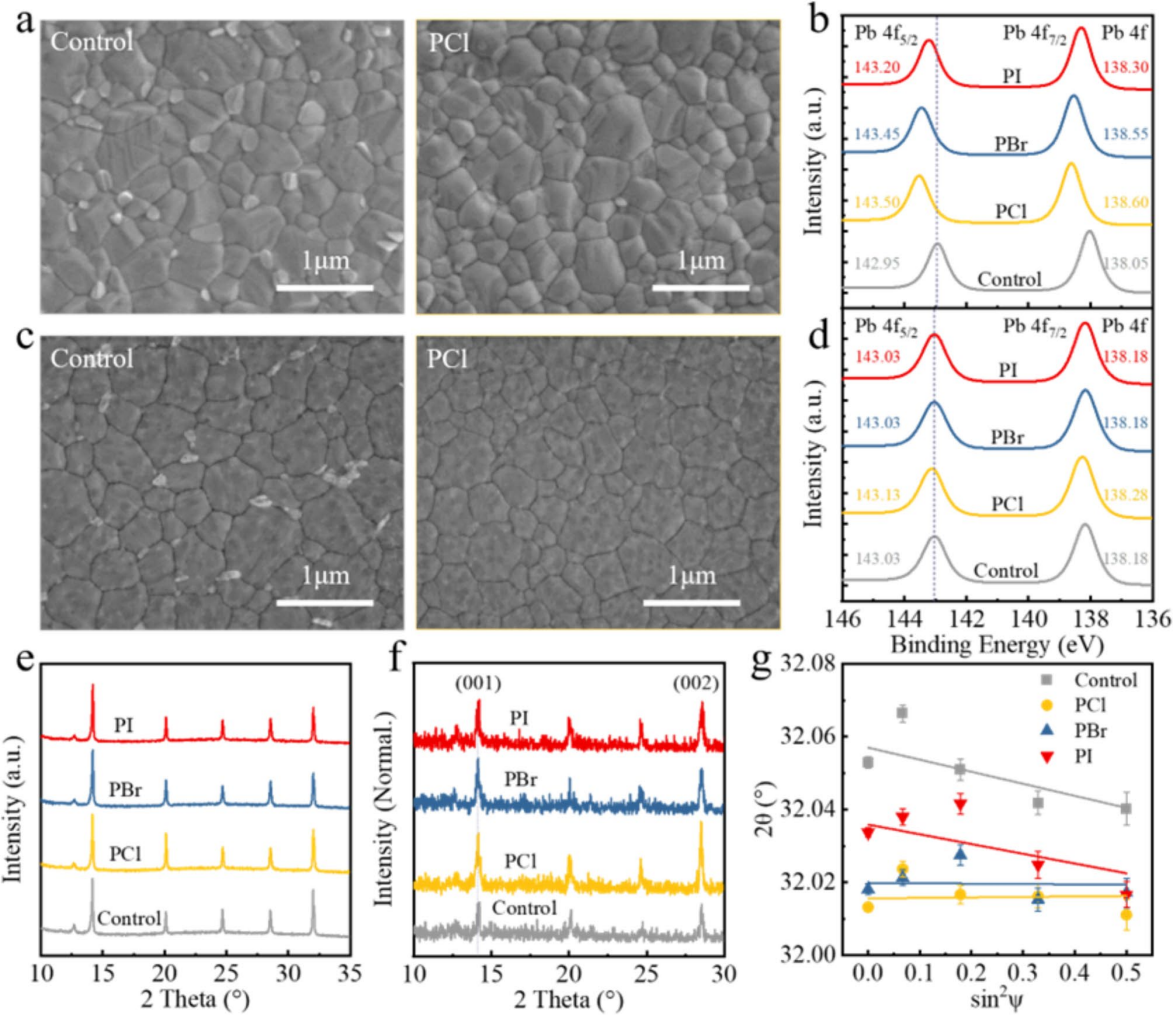
**a** Top-view SEM images of the top surface for the control and PCl-treated perovskite films. **b** Pb 4*f* XPS spectra of the top surface for the control, PCl, PBr and PI perovskite films. **c** Top-view SEM images of the buried interface for the control and PCl-treated perovskite films. **d** Pb 4*f* XPS spectra of the buried interface for the control, PCl, PBr and PI perovskite films. **e** XRD patterns of the control, PCl, PBr and PI perovskite films. **f** GIXRD patterns of the buried interface for the control, PCl, PBr and PI perovskite films. **g** Linear fit of 2*θ*–sin^2^ψ for control, PCl, PBr and PI perovskite films

We subsequently characterize the morphology and crystalline structure of the perovskite film to investigate the potential effects of PCl on the perovskite. SEM images show that the control perovskite film demonstrates discontinuous PbI_2_, filling the grain boundaries of the 3D perovskite (Fig. 2a). After treatment with PBr and PI, part of PbI_2_ at the grain boundaries is reacted (Fig. S16). However, much more PbI_2_ on the surface of the perovskite film reacted after being treated with PCl (Fig. 2a), indicating that PCl is more reactive with PbI_2_ due to the introduction of Cl^−^ with higher electronegativity [43, 44], thus demonstrating a stronger passivation effect than PBr and PI, which is supported by the XPS results (Figs. 2b and S17-S20). Additionally, the terraced structure of the control film is gradually smoothed out (Figs. 2a and S16), and the surface roughness is decreased (Fig. S21), implying that PCl possesses the strongest capability to interact with the perovskite grain surface among the three passivators [33]. The top-view SEM images of buried interfaces reveal that the control, PCl, PBr and PI perovskite films are basically similar in grain size and show no visible voids (Figs. 2c and S22). However, the film treated with PCl exhibits a tighter grain distribution and more completed PbI_2_ transformation, typically an indication of highly crystalline perovskite film [28], which is attributed to the diffusion of Cl^−^ ions inducing microscopic reconstruction and defect passivation of the perovskite buried interface[45], as evidenced by the XPS measurements (Figs. 2d and S23). The Cl^−^ enrichment at the perovskite buried interface after PCl treatment promotes the formation of strong Pb–Cl ionic bonds and compensates for halide ion defects. This results in an upshift of the Pb 4*f* and I 4*d* core levels, consequently providing additional passivation effects for the buried interface [28, 46].

The improvement in morphologies is correlated with the crystallinity of the perovskite films. XRD measurements demonstrate that no obvious peak shifts and/or new phases are observed, indicating negligible impact of PX (*X* = Cl, Br and I) on the bulk crystal quality of the entire perovskite films (Fig. 2e). However, the crystal crystallinity of perovskite films at the top region is gradually improved, as evidenced by grazing incident wide-angle X-ray scattering measurements (Figs. S24 and S25). Grazing incidence XRD (GIXRD) measurements were then performed at an incidence angle of 0.1° (detection depth of 30 nm) to evaluate the quality of perovskite films at the buried region, as shown in Fig. 2f. The buried interface of the control film suffers from poor crystallinity with very weak diffraction peaks for the (001) and (002) facets. After treatment with PBr and PI, the GIXRD diffraction signals slightly increase, which may be related to the microscopic reconstruction of the perovskite bottom interface assisted by the surface passivation [45]. In sharp contrast, the diffraction signals of (001) and (002) facets are markedly enhanced at the buried interface in the PCl-treated perovskite film, indicating higher crystallinity, which is stemmed from the diffusion of Cl^−^ ions in PCl to the bottom surface promoting grain regrowth [28]. Notably, the buried interface GIXRD of the PCl film maintains essentially identical diffraction angles to the control film with tiny offset, implying that Cl^−^ ions accumulating at the buried interface are not incorporated into the perovskite crystal lattice. Instead, they are located in the grain boundaries, promoting the crystal regrowth for the buried interface and passivating the residual defects, e.g., undercoordinated Pb ions or V_I_ defects [28, 46, 47].

Moreover, we found that the notable improvement in the morphology and crystallinity of the perovskite affects the release of residual stress [48]. As shown in Fig. S26, the diffraction peaks of the perovskite film treated with PI, PBr and PCl fitted with Gaussian distribution gradually shift to higher 2*θ* positions as ψ varies from 0° to 45°. The slopes of the fitted lines for the four films, determined by fitting the 2*θ* as a function of sin^2^ψ, are shown in Fig. 2g. The control, PI- and PBr-treated perovskite films exhibit negative slopes, indicating that the films are subjected to tensile stress [31]. In contrast, the slope of the PCl-treated perovskite film shows a diminutive positive value, suggesting a favorable situation with slight compressive stress (Table S2). These results indicate that the top-down carrier management strategy induced by PCl can alleviate the residual tensile stress of perovskite films, which is beneficial for the efficiency and stability of PSCs [48].

### Comprehensive Defect Passivation and Energy Band Modulation of Dual-Interfaces

**Fig. 3:**
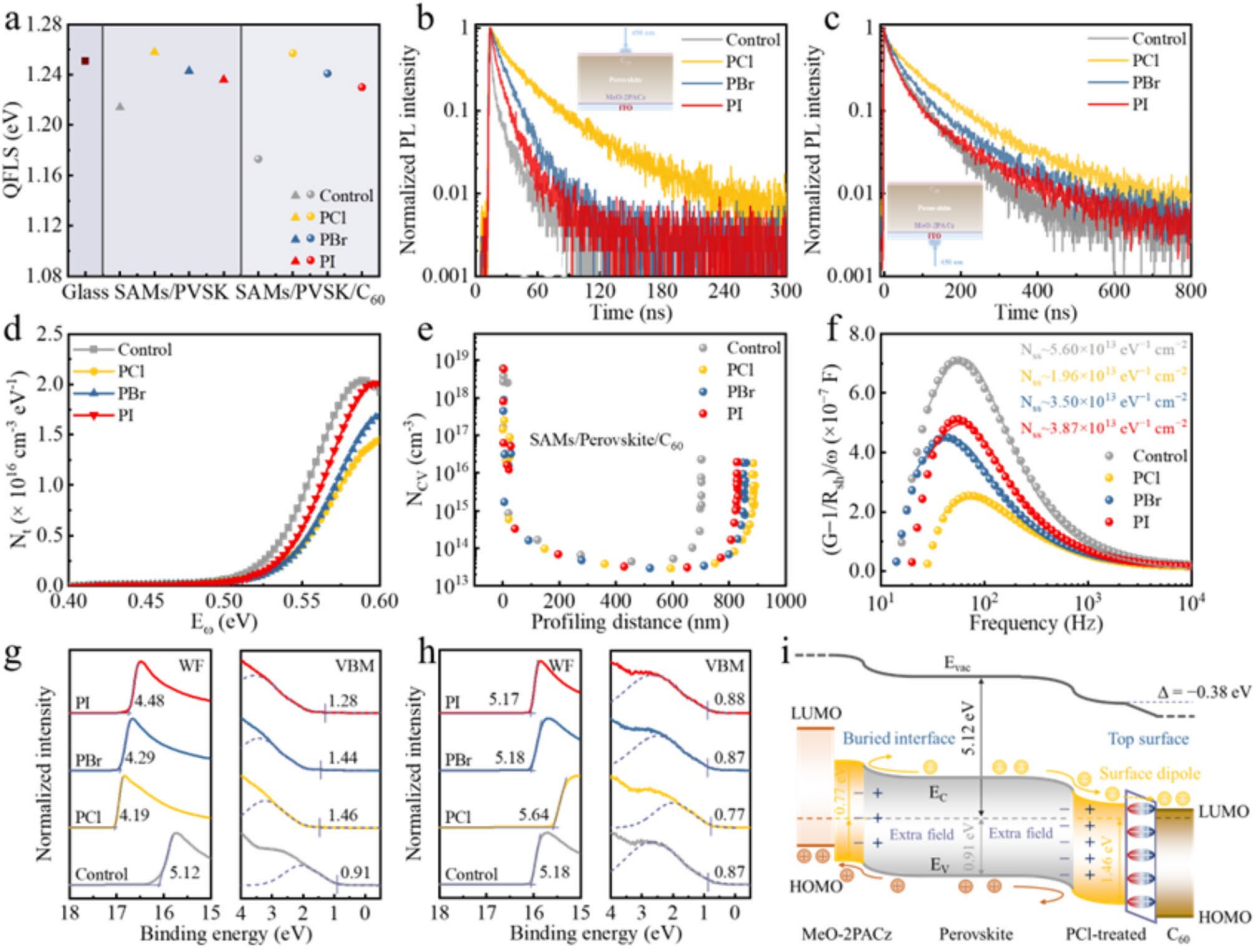
**a** QFLS diagram for perovskite film, ITO/MeO-2PACz/perovskite half stack and ITO/MeO-2PACz/perovskite/C_60_ full stack with and without post-treatments. **b** TRPL spectra of the glass/ITO/MeO-2PACz/perovskite/PX (*X* = Cl, Br and I)/C_60_ stacks with excitation from C_60_ side. **c** TRPL spectra of the glass/ITO/MeO-2PACz/perovskite/PX (*X* = Cl, Br and I)/C_60_ stacks with excitation from ITO side. **d** Trap density of states of control, PCl, PBr and PI devices. **e** Carrier distributions obtained by C–V measurements under low frequency (8 kHz) for control, PCl, PBr and PI devices. **f** Interface defect extraction of control, PCl, PBr and PI devices by using admittance measurement. **g** UPS spectra of the perovskite top surface for control, PCl, PBr and PI films. **h** UPS spectra of the perovskite buried interface for control, PCl, PBr and PI films. **i** Energy band diagrams of the PCl film, illustrating the negative surface dipole arrangement, band bending and built-in electric field distribution. *E*_V_, *E*_C_, WF, *E*_vac_, LUMO and HOMO represent the valence band maximum, conduction band minimum, work function, vacuum energy level, lowest unoccupied molecular orbital and highest occupied molecular orbital, respectively

Motivated by the improved morphology, crystalline structure, surface chemistry and residual stress following PCl treatments, absolute photoluminescence measurements were performed to obtain the photoluminescence quantum yield measurements (PLQY) and internal quasi-Fermi level splitting (QFLS) values of perovskite films, with and without charge transport layers, to evaluate the nonradiative recombination losses (Fig. 3a and Table S3). When directly depositing the perovskite on the quartz glass substrate, the neat perovskite exhibits a high QFLS of 1.251 eV. Upon adding MeO-2PACz to construct a glass/ITO/MeO-2PACz/perovskite half-stack configuration, the QFLS is visibly decreased to 1.214 eV, indicating relatively high recombination losses at the MeO-2PACz/perovskite interface [49, 50]. However, the QFLS values increase to 1.243 and 1.236 eV, respectively, after introducing PBr and PI onto the half stack, respectively. This suggests that PBr and PI modification reduce the nonradiative recombination losses of perovskite through chemical passivation, likely due to the reduced PbI_2_ on the surface as discussed previously [25, 31]. Remarkably, the QFLS value of the PCl-based half-stack further increases to 1.258 eV, even higher than that of the neat perovskite, which indicates that PCl effectively suppresses nonradiative recombination more than PBr and PI. To gain more insight into the surface recombination processes, the QFLS analyses of the full stack of glass/ITO/MeO-2PACz/perovskite/C_60_ were subsequently conducted. When capped with C_60_, the QFLS of the control stack dramatically decreased to 1.173 eV, reflecting serious energy loss at the perovskite/C_60_ interface [7, 51]. In contrast, the full stacks with PBr and PI maintain QFLS values of 1.241 and 1.230 eV, respectively, with minor energy losses, in line with previous reports [25]. Strikingly, when C_60_ is deposited on the PCl-based half stack, only a negligible decrease in QFLS (1.257 eV) is observed, implying that the C_60_-induced interfacial loss is nearly inhibited by PCl treatment. Briefly, the PCl post-treatment simultaneously reduces perovskite loss and interface loss, resulting in improved QFLS, which is expected to enhance the open-circuit voltage (*V*_OC_) and the fill factor (FF) in devices.

To gain more insight into charge dynamics, we subsequently conducted time-resolved photoluminescence (TRPL) measurements on glass/ITO/MeO-2PACz/perovskite/PX (*X* = Cl, Br and I)/C_60_ stacks, with excitation at 450 nm from both the C_60_ and ITO sides. When irradiated from the C_60_ side, as depicted in Fig. 3b, all stacks demonstrate a fast extraction process within 10 ns, followed by long tails governed by nonradiative recombination [10, 49]. The carrier lifetimes extracted from biexponential fits to the PL transients are listed in Table S4. The PBr and PI stacks show an increase in effective carrier lifetime from 2.25 ns (control) to 12.50 and 7.55 ns, respectively. Notably, the PCl stack exhibits the longest carrier lifetime of 25.26 ns, around 11 times enhancement compared to the control stack, indicative of efficient defect passivation of the perovskite top surface and decreased interfacial recombination velocity by PCl [52]. PL transients excited from the ITO sides are shown in Fig. 3c, and the corresponding decay times are obtained from biexponential decay fits (Table S5). Clearly, a fast decay component (*τ*_1_), originated from charge extraction at the interface, and a slow decay component (*τ*_2_), attributed to the nonradiative recombination, are distinguished [49]. The control stack exhibits the longest *τ*_1_ and lowest *τ*_2_, meaning the poor charge extraction and significant nonradiative recombination at the MeO-2PACz/perovskite buried interface [49, 50]. As expected, both the PBr and PI stacks display a lower *τ*_1_ and longer *τ*_2_ compared to the control stack. However, they demonstrate comparable nonradiative recombination lifetimes (*τ*_2_), likely because Br^−^ and I^−^ ions in PBr and PI not penetrate into the buried interfaces, and thus, nonradiative recombination defect sites remain at the bottom surfaces. The prolonged carrier nonradiative recombination lifetimes for PBr and PI stacks may be contributed to the improved crystallinity of the perovskite bottom interface resulting from the surface treatment [28, 45], consistent with the above GIXRD and SEM measurements. In contrast, the PCl stack shows the shortest *τ*_1_ and the longest *τ*_2_, indicating the best charge extraction and the least nonradiative recombination at the buried interface among all samples. This enhanced charge extraction and reduced nonradiative recombination at the buried interface in the PCl stack are attributed to the diffusion of Cl^−^ ions inducing the defect passivation [28, 53] and energetic alignment improvement, which will be confirmed later. Overall, the improved QFLS and mitigated nonradiative recombination in PBr and PI films are mainly ascribed to optimization of the perovskite/C_60_ interfaces. For the PCl perovskite film, the additional effects at the buried interface due to Cl^−^ ions are also significant, further passivating detrimental defects and increasing charge extraction at the buried interface. Therefore, PCl is expected to provide greater benefits to the photovoltaic performance of devices compared to PBr and PI.

The above observations collectively confirm the effective suppression of dual-interface nonradiative recombination in the PCl perovskite film, which is further verified by thermal admittance spectroscopy (TAS), capacitance–voltage (C–V) and interface defect density (*N*_SS_) measurements. Figure 3d demonstrates the trap density of states (tDOS) spectra for each device, estimated from the angular-frequency-dependent capacitance (Fig. S27). The traps at deep energy levels (~ 0.4–0.6 eV, corresponding to surface *V*_I_ defects [54, 55]) are significantly reduced in the PCl device, indicating effective passivation of charge traps in perovskite by PCl. The substantial reduction of surface *V*_I_ defects, featuring *n*-type doping effects [33, 56], in PCl devices is conducive to promoting *p*-type doping at the perovskite bottom surface. Moreover, the formation energy of *V*_I_ is calculated to increase after surface treatments (Fig. S28), which helps mitigate ion migration and enhances stability [57]. Further analysis is provided by C–V measurements, which assess free carrier, interface and bulk defects under low frequency (8 kHz) (Fig. 3e). A discernible decrease in the carrier profile residing on the surface region of the perovskite layer subsequent to the PCl post-treatment is revealed, substantiating that PCl effectively reduces interface defect density. Moreover, *N*_SS_ measurements, conducted using an admittance method to quantitatively extract interface defect profiles within devices (Fig. 3f) [58], show a dramatic decrease in *N*_SS_ for the PCl-modified device. This reduction further verifies the excellent dual-interface defect passivation provided by PCl, attributed to the bidirectional distribution of anions and cations of PCl. The efficient suppression of dual-interface defects in PCl device is shown to prolong the charge recombination lifetime and shorten the charge transport lifetime in devices (Fig. S29).

Next, ultraviolet photoelectron spectroscopy (UPS) measurements were conducted to reveal the surface energetics transformation of the top and bottom surfaces of perovskite films. Figure 3g shows the secondary electron cut-off energies and the valence band maxima (VBM) spectra of the top surface for these films. Treatments with PI and PBr reduce the work function (WF) from 5.12 to 4.48 and 4.29 eV and shift the VBM, determined by Gaussian fitting [59], from 0.91 to 1.28 and 1.44 eV, respectively. This results in downward energy band bending, which effectively facilitates electron extraction and hole blocking, as confirmed by previous works [25, 31, 60]. In contrast, treating the perovskite surface with PCl shifts the WF upward to 4.19 eV, with a VBM of 1.46 eV, suggesting a more pronounced n-type transformation of the perovskite top surface induced by PCl, which is further confirmed by Kelvin probe force microscopy (KPFM) measurements (Fig. S30). Combining these parameters with the optical bandgap of 1.68 eV (Fig. S31), the energy level diagrams of these films are depicted in Fig. S32. Notably, the formation of surface dipoles after surface treatment is observed. The ionization energy (I*) was measured to assess the surface dipoles of perovskites with different surface treatments [10]. The I* values are determined to be 6.03, 5.65, 5.73 and 5.76 eV for control, PCl, PBr and PI films, indicating the formation of a negative surface dipole. PI decreases I* by 0.27 eV, while PBr decreases it by 0.30 eV and PCl decreases by 0.38 eV, underscoring the strongest electrostatic dipole effect of PCl among all samples. Obviously, the more negative surface dipole formed in the PCl film induces a more significant upward shift in the WF [26, 27], thereby creating a stronger built-in electric field for driving the electrons from the perovskite to C_60_ and repelling the holes back to the perovskite layer. In summary, the improved energy band bending and enhanced surface dipole of the PCl-treated top surface not only increase electron selectivity, but also enhance the electrical passivation through an additional field effect, thereby efficiently suppressing the across-interface recombination [7, 10].

**Fig. 4:**
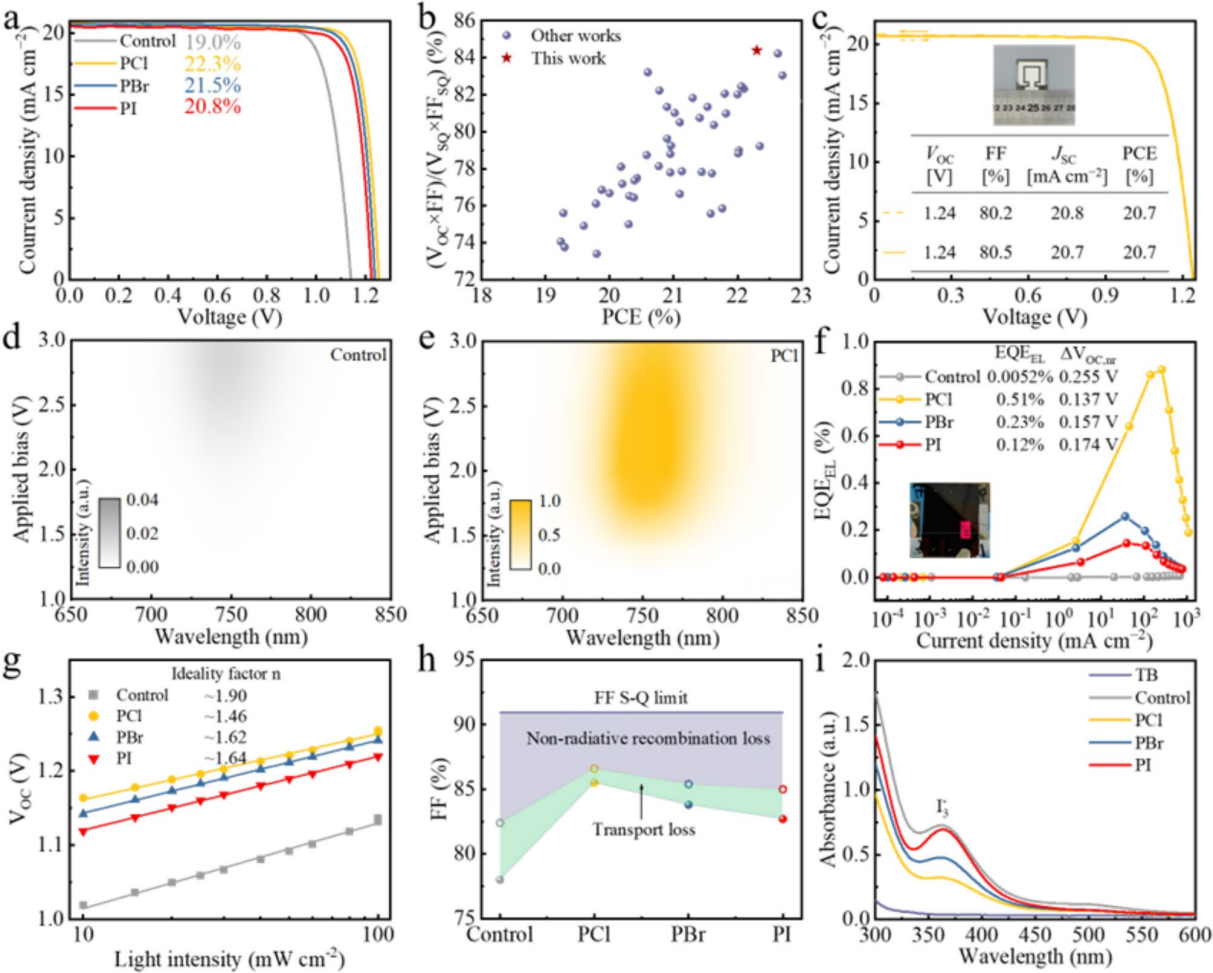
**a** *J–V* curves of the champion control, PCl, PBr and PI single-junction PSCs under simulated AM1.5G solar illumination (100 mW cm^−2^). **b** Plots of (*V*_OC_ × FF)/(*V*_OC,SQ_ × FF_SQ_) against PCE for 1.68 eV PSCs in this work and reported literature from Table S5. **c** *J–V* curve of the champion PCl PSC with an aperture area of 1.21 cm^2^. **d** EL spectra of the control PSC under different applied voltage biases. **e** EL spectra of the PCl PSC under different applied voltage biases. **f** EQE-EL spectra of the control, PCl, PBr and PI devices operating in light-emitting diodes. **g** Light intensity dependent *V*_OC_ values. **h** The device FF S-Q limit, consisting of nonradiative recombination and charge transport losses. **i** UV–Vis absorption spectra of the toluene solutions in which control, PCl, PBr and PI perovskite films were immersed under 1-sun illumination for 24 h

Further UPS measurements were conducted to reveal the surface energetic changes at the buried interfaces of different perovskite films [61, 62], as shown in Figs. 3i and S33. The buried interface of the control film exhibits a weak *n*-type semiconductor characteristic with a WF of 5.18 eV and VBM of 0.87 eV. After treating with PBr and PI, both films demonstrate negligible energetic changes, with similar WFs of 5.18 and 5.17 eV and VBMs of 0.87 and 0.88 eV, respectively. On the contrary, the PCl film exhibits a distinct *p*-type buried interface with a deeper WF of 5.64 eV and a lower VBM of 0.77 eV, implying a transformation from *n*-type to *p*-type at the buried interface and the formation of an additional field. The *p*-type transformation of the buried interface after PCl treatment is ascribed to the diffusion of Cl^−^ ions into the buried interface, filling the *n*-type *V*_I_ defects, which is beneficial for accelerating hole transfer and reducing energy loss at the buried interface. Overall, UPS measurements imply that PCl post-treatment simultaneously optimizes the energy level arrangement and enhances the built-in electric field (Fig. S34) at both interfaces, as schematically demonstrated in Figs. 4f, S35 and S36, which is favorable for accelerating charge transfer and separation, thereby increasing both *V*_OC_ and FF. Overall, the above evidences point to, in comparison with PBr and PI, a bidirectional cation/anion interfacial distribution of PCl and its stronger chemical bonding with defects as responsible to the realization of dual-interfacial defect passivation and energy band modulation.

### Performance of Perovskite Single-Junction Device

To demonstrate the effectiveness of the top-down carrier management induced by PCl at the device level, inverted perovskite solar cells (PSCs) with the architecture of glass/ITO/MeO-2PACz/Cs_0.05_(FA_0.77_MA_0.23_)_0.95_Pb(I_0.77_Br_0.23_)_3_/PX (*X* = Cl, Br, I)/C_60_/BCP/Ag were fabricated (Fig. S37). The optimization processes of PX (*X* = Cl, Br and I) are summarized in Figs. S1, S38, S39 and Tables S1, S6 and S7, respectively. Figure S40 and Table S8 compare the photovoltaic (PV) parameters of control, PCl-, PBr- and PI-modified PSCs. As anticipated, the enhancement in power conversion efficiency (PCE) primarily lies in the *V*_OC_ and FF, and PCl devices demonstrate the highest *V*_OC_ and FF with good reproducibility, indicating more effective carrier management. The current density–voltage (*J–V*) curves of the champion control, PCl-, PBr- and PI-based devices are displayed in Fig. 4a. The PCl-based device achieves a champion PCE of 22.3%, with a high *V*_OC_ of 1.254 V and an FF of 85.6% under reverse scan, which surpasses the champion PCEs of PBr- and PI-based devices, while the control device shows a lower PCE of only 19.0%. The steady-state PCEs for the control, PCl-, PBr- and PI-based devices are 18.8%, 21.8%, 20.9% and 20.4%, respectively (Fig. S41). Notably, the champion PCl device demonstrates an impressive *V*_OC_ × FF product, reaching 84.4% relative to the Shockley–Queisser limit, the highest value reported for a 1.68 eV p-i-n device to date (Fig. 4b and Table S9). The integrated current densities calculated from external quantum efficiency (EQE) spectrum of the corresponding devices are consistent with the values extracted from *J–V* measurements (Fig. S42). We further fabricated PCl-based PSCs on larger substrates with device aperture area of 1.21 cm^2^ (Fig. 4c). The best-performing PCl device achieves a PCE of 20.7% under reverse scan, with a *V*_OC_ of 1.24 V and an FF of 80.5%. The generality of PCl to various perovskite compositions and HTL substrates is also demonstrated (Figs. S43 and S44). The above results highlight the scalability and universality of PCl, facilitating the large-area fabrication of perovskite-based tandem solar cells.

A quantitative estimation of the *V*_OC_ deficit based on the detailed balance theory was conducted, using a complete device as a light-emitting diode. Figures 4d, e and S45 show electroluminescence (EL) spectra of different devices under various applied biases. The EL intensities were normalized to the highest value across all spectra. It is evident that the PCl, PBr and PI devices start to work at lower applied biases than the control device, indicating effective suppression of leakage current after PCl, PBr and PI surface treatments. Notably, the PCl device exhibits higher emission intensity at each applied bias compared to the others, suggesting significantly reduced nonradiative recombination in the PCl devices. Furthermore, in Fig. 4f, under a forward bias injection current density equal to the 1-sun *J*_SC_ (~ 20.8 mA cm^−2^), the PCl, PBr and PI devices determines external quantum efficiency of electroluminescence (EQE_EL_) values of 0.51%, 0.23% and 0.12%, corresponding to nonradiative recombination losses of *V*_OC_ (∆*V*_OC,nr_) of 0.137, 0.157 and 0.174 V, respectively. Following the detailed balance limit model, the theoretical radiative limit of *V*_OC_ (*V*_OC,rad_) has been calculated to be 1.399 V for Cs_0.05_(FA_0.77_MA_0.23_)_0.95_Pb(I_0.77_Br_0.23_)_3_ perovskite with a bandgap of 1.68 eV [63]. Consequently, the calculated *V*_OC_s for the PCl, PBr and PI devices are derived to be 1.262, 1.242 and 1.225 V, respectively, which are close to the *V*_OC_s obtained from *J–V* curves. In comparison, the control device displays a much lower EQE_EL_ of 0.0052%, which corresponds to a much higher ∆*V*_OC,nr_ of 0.255 V, leading to a calculated *V*_OC_ of 1.144 V. Obviously, while PBr and PI treatments substantially suppress nonradiative recombination at the perovskite/C_60_ interface, resulting in *V*_OC_ enhancement, PCl provides a more comprehensive defect passivation effect due to additional defect healing and energy improvement at the buried interface, which increases the *V*_OC_, as also reflected by the QFLS measurements.

Subsequently, the FF enhancement was quantitatively analyzed using the Suns-*V*_OC_ method. As shown in Fig. 4g, the PBr and PI devices exhibit smaller ideality factors (*n*) of 1.62 and 1.64, respectively, compared to the control device (1.90), suggesting suppressed trap-assisted nonradiative recombination. The PCl device shows the smallest ideality factor of 1.46 among the four devices, demonstrating the most effective suppression of nonradiative recombination and the greatest FF enhancement. Furthermore, the FF deficit was explored, as shown in Fig. 4h. The calculated FF_max_ values for the control, PCl, PBr and PI devices are 82.4%, 86.6%, 85.4% and 85.0%, respectively. Accordingly, the nonradiative recombination loss decreases from 8.52% for the control device to 4.32%, 5.52% and 5.92% for PCl, PBr and PI devices, respectively, and the charge transport loss also decreases from 4.4% to 1.1%, 1.6% and 2.3%, respectively. Obviously, both trap-assisted nonradiative recombination loss and charge transport loss have been definitely inhibited by piperazinium halide post-treatments, with PCl being the most effective. This enhanced performance is ascribed to the improved defect passivation and favorable band alignments at both interfaces.

In addition to the enhanced PV performance, the improved stability of PCl-treated perovskite films and devices was also observed. As shown in Fig. S46, the control, PBr and PI films exhibit significant perovskite decomposition, with obvious raise in the PbI_2_ characteristic peak at 12.7° after storage for 2400 h at ambient conditions (40%–60% high relative humidity (RH) and temperature range from 25 to 35 °C). On the contrary, PCl-treated perovskite film still maintains the perovskite diffraction peak at 14.2° under the same condition, suggesting improved humidity stability. When subjected to thermal aging at 85 °C with a low humidity of 10%–15% RH for 500 h, the PCl perovskite film presents the lowest peak area ratio of PbI_2_ to perovskite in comparison with the control, PBr and PI perovskite films (Fig. S47), indicating superior thermal stability. Furthermore, light stability was investigated by immersing the four perovskite films into toluene to extract the I_2_ generated during light soaking. After 24 h of continuous light soaking at 1-sun light intensity, the PCl film shows the lowest intensity of UV–Vis absorption spectra of I_2_ (~ 500 nm) and I_3_^−^ (~ 365 nm) [64] (Fig. 4i). As revealed in previous reports, the release of I_2_ and I_3_^−^ from perovskite film during light soaking was mainly attributed to interstitial iodide (I_i_) or PbI_2_ defects [65, 66]. The PCl treatment effectively eliminates PbI_2_ defects within the perovskite films, leading to reduced I_2_ formation. Additionally, PCl treatment results in a noticeable reduction in residual tensile stress within the perovskite film (Figs. 2g and S26), further enhancing the formation energy of I_i_ defects [67]. As a result, the PCl films demonstrate higher resistance to illumination-induced degradation.

The impact of PCl on device stability was also evaluated. The performance of unencapsulated solar cells was tracked under open-circuit conditions in ambient air with a RH of 40%–60% and a temperature of 30 ± 5 °C, resembling the aging conditions of the International Summit on Organic Photovoltaic Stability (ISOS) protocol, ISOS-D-1. Following 2160 h of aging, the PCl devices exhibit a low PCE loss of 10.5% relative to the original PCE, compared to 27.1%, 19.0% and 20.9% efficiency losses for the control, PBr and PI devices (Fig. S48). Subsequently, devices were tested under prolonged heat exposure at 85 °C in nitrogen, following the ISOS-D-2 procedure (Fig. S49). PCl devices retain 88.7% of their initial PCE after 960 h of continuous heating, whereas the control, PBr and PI devices maintain 68.0%, 83.3% and 81.4% of their initial PCEs, respectively. Operational stability was characterized under AM 1.5G continuous 1-sun illumination (white LED) at various time intervals (30 ± 5 °C, 40%–60% RH) (ISOS-L-1). The PCl devices show superior operational stability with over 91% of the initial PCE after maximum power point (MPP) operation for 1200 h (Fig. S50), compared to 85% and 80% PCEs retention for PBr and PI devices. On the contrary, the PCE of control devices rapidly decays to only 58.8% of its initial efficiency after 1160 h. The improved operational stability of the PCl device is attributed to the reduced I_2_ formation during light soaking, resulting from lower PbI_2_ defects and less residual tensile stress within the PCl perovskite film [68].

### Performance of Monolithic Perovskite/Silicon Tandem Device

**Fig. 5:**
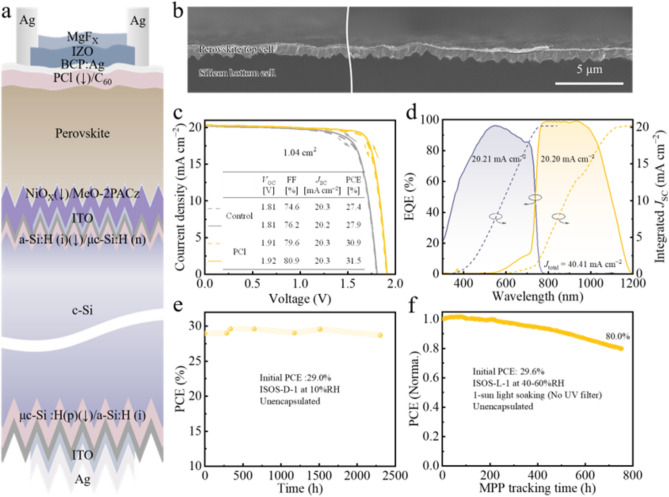
**a** Schematic illustration of the perovskite/silicon heterojunction tandem solar cell (PVSK/Si TSC) architecture. **b** Cross-section SEM image of the PVSK/Si TSC in a broader area. **c** *J–V* curves of the champion control and PCl-based PVSK/Si TSCs with an aperture area of 1.04 cm^2^. The inset table shows the corresponding efficiency parameters. **d** EQE spectrum of the champion PCl-based PVSK/Si TSC. **e** Shelf stability of the unencapsulated PCl-based PVSK/Si TSC in N_2_ (25–35 °C, 10% RH). **f** The operational stability of the unencapsulated PCl-based PVSK/Si TSC at maximum power point under continuous AM1.5G xenon lamp illumination without any UV–blocking filter in air (25–35 °C, 40%–60% RH)

Encouraged by the good homogeneity and promising performance in single-junction (SJ) devices imparted by PCl. We proceeded to fabricate monolithic perovskite/silicon tandem solar cells (PVSK/Si TSCs) with the following stack: silicon heterojunction (SHJ)/indium tin oxide (ITO)/sputtered nickel oxide (NiO_X_)/MeO-2PACz/1.68-eV Cs_0.05_(FA_0.77_MA_0.23_)_0.95_Pb(I_0.77_Br_0.23_)_3_/PCl/C_60_/BCP:Ag/IZO/Ag/MgF_*X*_, as schematically sketched in Fig. 5a. To minimize shunt loss at the buried interface and enhance the adsorption of MeO-2PACz, the hole-selective contact was switched from MeO-2PACz monolayer to a sputtered NiO_*X*_/MeO-2PACz hybrid bilayer [69]. The cross-section SEM image of the fabricated tandem device is shown in Fig. 5b, featuring a double-side textured SHJ bottom cell. Broader top-view and cross-section SEM images of the bottom SHJ cell are depicted in Figs. S51 and S52. The SHJ bottom cell is featured with a front-side random sub-micron pyramid structure and a rear-side micro-pyramidal structure, which is conducive to enhancing the near-infrared absorption of SHJ and enabling the deposition of spin-coated perovskite [70]. Figure S53 exhibits the image of PVSK/Si TSCs visually with an aperture area of 1.04 cm^2^.

The improvement in tandem structures is primarily attributed to enhancements in *V*_OC_ and FF as discussed above. The *J–V* curves of the champion tandem devices for the control and PCl are sketched in Fig. 5c, with corresponding PV parameters summarized in the inset. The control device yields a champion PCE of 27.9% (27.4%) with a *V*_OC_ of 1.81 (1.81) V, an FF of 76.2% (74.6%) and a *J*_SC_ of 20.2 (20.3) mA cm^−2^ under reverse (forward) scan, showing noticeable hysteresis. Encouragingly, the best PCl tandem delivers a remarkable PCE of 31.5% (30.9%), with a *V*_OC_ of 1.92 (1.91) V, an FF of 80.9% (79.6%) and a *J*_SC_ of 20.3 (20.3) mA cm^−2^ under reverse (forward) scan, featuring negligible hysteresis, which is benefited from the reduced nonradiative recombination and improved band alignment of PCl-induced dual-interface. The impressive PCE over 31% is one of the highest values for reported textured PVSK/Si TSCs (Table S10), highlighting the significant effectiveness of our top-down carrier management strategy. The in-house steady-state PCE of the champion PCl tandem reaches 31.2% (Fig. S54). The statistic of PCE shows an average PCE of 30.6%, demonstrating the high reproducibility of the top-down dual-interface carrier management strategy facilitated by PCl (Fig. S55). The integrated *J*_SC_ of the perovskite and silicon sub-cells are 20.21 and 20.20 mA cm^−2^ (Fig. 5d), respectively, matching well with the *J–V* results. Additionally, one of the best PCl-based tandems was sent to Shanghai Institute of Micro-system and Information Technology (SIMIT) for independent certification to verify our in-house measurement. We successfully attained a third-party certified steady-state PCE of 30.4% (for the 5-min steady-state MPP tracking), with a PCE of 30.5% (30.7%), *V*_OC_ of 1.90 (1.91) V, *J*_SC_ of 20.3 (20.3) mA cm^−2^ and FF of 79.4% (79.2%) under reverse (forward) scan (Fig. S56).

We finally evaluated the long-term stability of PCl-based PVSK/Si TSCs using ISOS-D-1 and ISOS-L-1 procedures, as shown in Fig. 5e, f. After 2304 h of exposure to a temperature of 30 ± 5 °C and 10% RH conditions (ISOS-D-1), the unencapsulated PCl tandem retains 99.0% of its initial PCE (29.0%) (Figs. 5e and S57). Moreover, under continuous 1-sun xenon lamp illumination (without a UV filter) at 30 ± 5 °C and 40%–60% RH (ISOS-L-1), the unencapsulated PCl tandem device maintains 80.0% of the original PCE (29.6%) after 755 h (Fig. 5f), showing a decent operational stability. Notably, this is one of the best stabilities achieved in an unencapsulated tandem device at MPP tracking under similar test situation (Table S11). Nevertheless, the stability of PVSK/Si TSCs still lags behind perovskite SJ devices under extended light soaking, a likely possibility being the current mismatch between sub-cells inducing charge accumulation at interfaces, accelerating device degradation [71]. In our work, since the silicon bottom cell is the limiting cell (Fig. 5d) and thus the photoexcited charge carriers in the perovskite top cell are not all extracted, causing additional instability (Fig. S58), therefore, further exploration is urgently needed to optimize and accurately evaluate tandems in this regard. Overall, the high potential of this top-down dual-interface carrier management strategy induced by PCl for achieving high efficiency and scalable fabrication of PVSK/Si TSCs has been demonstrated, which is expected to advance the commercialization of PVSK/Si TSCs.

## Conclusions

A simple top-down dual-interface carrier management strategy has been successfully employed to improve both interfaces of the wide-bandgap perovskites, achieved through a one-step post-treatment using multifunctional piperazinium chloride. We have demonstrated that piperazinium cations with large steric hindrance primarily enrich on the perovskite top surface, while chloride anions with smaller ionic radii spontaneously migrate downward and accumulate at the buried interface, forming a bidirectional ions distribution. The enrichment of piperazinium cations at the top surface effectively mitigates surface defects and creates a negative surface dipole, thereby suppressing nonradiative recombination and improving energy band alignment. Meanwhile, the accumulation of chloride anions at the buried interface forms strong Pb–Cl bonds and induces a *p*-type transformation of the surface energetics, which heals defects and reduces energy offset. This dual-interface management strategy results in a champion PCE of 22.3% for the 1.68 eV perovskite single-junction device, achieving a record *V*_OC_ × FF product and demonstrating excellent stability. Additionally, an efficient and stable monolithic perovskite/silicon tandem solar cell based on a silicon heterojunction bottom cell with an impressive PCE of 31.5% is achieved. This strategy provides a promising pathway on dual-interface engineering for achieving efficient perovskite-based tandems and may be extended in future applications.

## Supplementary Information

Below is the link to the electronic supplementary material.

Supplementary file1 (DOCX 10419 KB)

## References

[1] M.A. Green, E.D. Dunlop, M. Yoshita, N. Kopidakis, K. Bothe et al., Solar cell efficiency tables (Version 64). Prog. Photovolt. Res. Appl. **32**(7), 425–441 (2024). 10.1002/pip.3831

[2] M.H. Futscher, B. Ehrler, Efficiency limit of perovskite/Si tandem solar cells. ACS Energy Lett. **1**, 863–868 (2016). 10.1021/acsenergylett.6b00405

[3] Z. Ying, X. Yang, X. Wang, J. Ye, Towards the 10-year milestone of monolithic perovskite/silicon tandem solar cells. Adv. Mater. **36**, e2311501 (2024). 10.1002/adma.202311501 39049723 10.1002/adma.202311501

[4] W. Peng, K. Mao, F. Cai, H. Meng, Z. Zhu et al., Reducing nonradiative recombination in perovskite solar cells with a porous insulator contact. Science **379**, 683–690 (2023). 10.1126/science.ade3126 36795834 10.1126/science.ade3126

[5] H. Lin, M. Yang, X. Ru, G. Wang, S. Yin et al., Silicon heterojunction solar cells with up to 26.81% efficiency achieved by electrically optimized nanocrystalline-silicon hole contact layers. Nat. Energy **8**, 789–799 (2023). 10.1038/s41560-023-01255-2

[6] M. Stolterfoht, C.M. Wolff, J.A. Márquez, S. Zhang, C.J. Hages et al., Visualization and suppression of interfacial recombination for high-efficiency large-area pin perovskite solar cells. Nat. Energy **3**, 847–854 (2018). 10.1038/s41560-018-0219-8

[7] J. Warby, F. Zu, S. Zeiske, E. Gutierrez-Partida, L. Frohloff et al., Understanding performance limiting interfacial recombination in *pin* perovskite solar cells. Adv. Energy Mater. **12**, 2103567 (2022). 10.1002/aenm.202103567

[8] A.J. Ramadan, R.D.J. Oliver, M.B. Johnston, H.J. Snaith, Methylammonium-free wide-bandgap metal halide perovskites for tandem photovoltaics. Nat. Rev. Mater. **8**, 822–838 (2023). 10.1038/s41578-023-00610-9

[9] J. Liu, M. De Bastiani, E. Aydin, G.T. Harrison, Y. Gao et al., Efficient and stable perovskite-silicon tandem solar cells through contact displacement by MgF_*x*_. Science **377**, 302–306 (2022). 10.1126/science.abn8910 35737811 10.1126/science.abn8910

[10] S. Mariotti, E. Köhnen, F. Scheler, K. Sveinbjörnsson, L. Zimmermann et al., Interface engineering for high-performance, triple-halide perovskite-silicon tandem solar cells. Science **381**, 63–69 (2023). 10.1126/science.adf5872 37410849 10.1126/science.adf5872

[11] X.Y. Chin, D. Turkay, J.A. Steele, S. Tabean, S. Eswara et al., Interface passivation for 31.25%-efficient perovskite/silicon tandem solar cells. Science **381**, 59–63 (2023). 10.1126/science.adg0091 37410835 10.1126/science.adg0091

[12] K. Artuk, D. Turkay, M.D. Mensi, J.A. Steele, D.A. Jacobs et al., A universal perovskite/C60 interface modification *via* atomic layer deposited aluminum oxide for perovskite solar cells and perovskite-silicon tandems. Adv. Mater. **36**, e2311745 (2024). 10.1002/adma.202311745 38300183 10.1002/adma.202311745

[13] Z.-W. Gao, Y. Wang, W.C.H. Choy, Buried interface modification in perovskite solar cells: a materials perspective. Adv. Energy Mater. **12**, 2104030 (2022). 10.1002/aenm.202104030

[14] Q. Jiang, K. Zhu, Rapid advances enabling high-performance inverted perovskite solar cells. Nat. Rev. Mater. **9**, 399–419 (2024). 10.1038/s41578-024-00678-x

[15] F.H. Isikgor, S. Zhumagali, L.V.T. Merino, M. De Bastiani, I. McCulloch et al., Molecular engineering of contact interfaces for high-performance perovskite solar cells. Nat. Rev. Mater. **8**, 89–108 (2022). 10.1038/s41578-022-00503-3

[16] D. Turkay, K. Artuk, X.-Y. Chin, D.A. Jacobs, S.-J. Moon et al., Synergetic substrate and additive engineering for over 30%-efficient perovskite-Si tandem solar cells. Joule **8**, 1735–1753 (2024). 10.1016/j.joule.2024.04.015

[17] X. Zhang, Q. Ma, Y. Wang, J. Zheng, Q. Liu et al., Ligand homogenized Br-I wide-bandgap perovskites for efficient NiO_*x*_-based inverted semitransparent and tandem solar cells. ACS Nano **18**, 15991–16001 (2024). 10.1021/acsnano.4c04341 38829730 10.1021/acsnano.4c04341

[18] L. Qiao, T. Ye, T. Wang, W. Kong, R. Sun et al., Freezing halide segregation under intense light for photostable perovskite/silicon tandem solar cells. Adv. Energy Mater. **14**, 2302983 (2024). 10.1002/aenm.202302983

[19] F. Sahli, J. Werner, B.A. Kamino, M. Bräuninger, R. Monnard et al., Fully textured monolithic perovskite/silicon tandem solar cells with 25.2% power conversion efficiency. Nat. Mater. **17**, 820–826 (2018). 10.1038/s41563-018-0115-4 29891887 10.1038/s41563-018-0115-4

[20] S. Yuan, K. Mao, F. Cai, Z. Zhu, H. Meng et al., Understanding and engineering the perovskite/organometallic hole transport interface for high-performance p–i–n single cells and textured tandem solar cells. ACS Energy Lett. **9**, 3557–3566 (2024). 10.1021/acsenergylett.4c01301

[21] J. Li, B. Farhadi, S. Liu, L. Liu, H. Wang et al., Built-in field manipulation through a perovskite homojunction for efficient monolithic perovskite/silicon tandem solar cells. Nano Energy **129**, 109976 (2024). 10.1016/j.nanoen.2024.109976

[22] R. Azmi, D.S. Utomo, B. Vishal, S. Zhumagali, P. Dally et al., Double-side 2-dimensional/3-dimensional heterojunctions for inverted perovskite solar cells. Nature **628**, 93–98 (2024). 10.1038/s41586-024-07189-3 38382650 10.1038/s41586-024-07189-3

[23] N. Yan, Y. Gao, J. Yang, Z. Fang, J. Feng et al., Wide-bandgap perovskite solar cell using a fluoride-assisted surface gradient passivation strategy. Angew. Chem. Int. Ed. **62**, e202216668 (2023). 10.1002/anie.202216668 10.1002/anie.202216668 36593561

[24] D.B. Khadka, Y. Shirai, M. Yanagida, H. Ota, A. Lyalin et al., Defect passivation in methylammonium/bromine free inverted perovskite solar cells using charge-modulated molecular bonding. Nat. Commun. **15**, 882 (2024). 10.1038/s41467-024-45228-9 38287031 10.1038/s41467-024-45228-9 PMC10824754

[25] F. Yang, P. Tockhorn, A. Musiienko, F. Lang, D. Menzel et al., Minimizing interfacial recombination in 1.8 eV triple-halide perovskites for 27.5% efficient all-perovskite tandems. Adv. Mater. **36**, e2307743 (2024). 10.1002/adma.202307743 37988595 10.1002/adma.202307743

[26] T.C. Leung, C.L. Kao, W.S. Su, Y.J. Feng, C.T. Chan, Relationship between surface dipole, work function and charge transfer: Some exceptions to an established rule. Phys. Rev. B **68**, 195408 (2003). 10.1103/physrevb.68.195408

[27] M. Zhang, Q. Chen, R. Xue, Y. Zhan, C. Wang et al., Reconfiguration of interfacial energy band structure for high-performance inverted structure perovskite solar cells. Nat. Commun. **10**, 4593 (2019). 10.1038/s41467-019-12613-8 31597916 10.1038/s41467-019-12613-8 PMC6785549

[28] L. Shen, P. Song, L. Zheng, L. Wang, X. Zhang et al., Ion-diffusion management enables all-interface defect passivation of perovskite solar cells. Adv. Mater. **35**, e2301624 (2023). 10.1002/adma.202301624 37358373 10.1002/adma.202301624

[29] X. Jiang, B. Liu, X. Wu, S. Zhang, D. Zhang et al., Top-down induced crystallization orientation toward highly efficient p-i-n perovskite solar cells. Adv. Mater. **36**, e2313524 (2024). 10.1002/adma.202313524 38453665 10.1002/adma.202313524

[30] C. Luo, G. Zheng, F. Gao, X. Wang, C. Zhan et al., Engineering the buried interface in perovskite solar cells *via* lattice-matched electron transport layer. Nat. Photon. **17**, 856–864 (2023). 10.1038/s41566-023-01247-4

[31] F. Li, X. Deng, F. Qi, Z. Li, D. Liu et al., Regulating surface termination for efficient inverted perovskite solar cells with greater than 23% efficiency. J. Am. Chem. Soc. **142**, 20134–20142 (2020). 10.1021/jacs.0c09845 33190487 10.1021/jacs.0c09845

[32] Y. Zheng, X. Wu, R. Zhuang, C. Tian, A. Sun et al., Managing interfacial hot-carrier cooling and extraction kinetics for inverted ma-free perovskite solar cells over 23% efficiency *via* Dion–Jacobson 2D capping layer. Adv. Funct. Mater. **33**, 2300576 (2023). 10.1002/adfm.202300576

[33] Q. Jiang, J. Tong, Y. Xian, R.A. Kerner, S.P. Dunfield et al., Surface reaction for efficient and stable inverted perovskite solar cells. Nature **611**, 278–283 (2022). 10.1038/s41586-022-05268-x 36049505 10.1038/s41586-022-05268-x

[34] X. Liu, T. Webb, L. Dai, K. Ji, J.A. Smith et al., Influence of halide choice on formation of low-dimensional perovskite interlayer in efficient perovskite solar cells. Energy Environ. Mater. **5**, 670–682 (2022). 10.1002/eem2.12321

[35] D. Luo, W. Yang, Z. Wang, A. Sadhanala, Q. Hu et al., Enhanced photovoltage for inverted planar heterojunction perovskite solar cells. Science **360**, 1442–1446 (2018). 10.1126/science.aap9282 29954975 10.1126/science.aap9282

[36] X. Wang, Y. Wang, Y. Chen, X. Liu, Y. Zhao, Efficient and stable CsPbI_3_ inorganic perovskite photovoltaics enabled by crystal secondary growth. Adv. Mater. **33**, e2103688 (2021). 10.1002/adma.202103688 34515363 10.1002/adma.202103688

[37] S. Tan, C. Tan, Y. Cui, B. Yu, Y. Li et al., Constructing an interfacial gradient heterostructure enables efficient CsPbI_3_ perovskite solar cells and printed minimodules. Adv. Mater. **35**, e2301879 (2023). 10.1002/adma.202301879 37022759 10.1002/adma.202301879

[38] J. Fang, D. Lin, G. Xie, S. Li, H. Li et al., Anion exchange promoting non-impurities enables conformable and efficient inverted perovskite solar cells. Energy Environ. Sci. **17**, 7829–7837 (2024). 10.1039/d4ee02718c

[39] M.A. Mahmud, H.T. Pham, T. Duong, Y. Yin, J. Peng et al., Combined bulk and surface passivation in dimensionally engineered 2D–3D perovskite films *via* chlorine diffusion. Adv. Funct. Mater. **31**, 2104251 (2021). 10.1002/adfm.202104251

[40] X. Zhang, W. Zhou, X. Chen, Y. Chen, X. Li et al., Dual optimization of bulk and surface *via* guanidine halide for efficient and stable 2D/3D hybrid perovskite solar cells. Adv. Energy Mater. **12**, 2201105 (2022). 10.1002/aenm.202201105

[41] C. Luo, G. Zheng, X. Wang, F. Gao, C. Zhan et al., Solid–solid chemical bonding featuring targeted defect passivation for efficient perovskite photovoltaics. Energy Environ. Sci. **16**, 178–189 (2023). 10.1039/d2ee02732a

[42] Y. Zhang, Y. Wang, L. Zhao, X. Yang, C.-H. Hou et al., Depth-dependent defect manipulation in perovskites for high-performance solar cells. Energy Environ. Sci. **14**, 6526–6535 (2021). 10.1039/d1ee02287c

[43] H. Wu, X. Yin, L. Lu, J. Song, L. Hu et al., Cation and anion optimization of ammonium halide for interfacial passivation of inverted perovskite solar cells. Chem. Commun. **59**, 6183–6186 (2023). 10.1039/D3CC01195J 10.1039/d3cc01195j 37128955

[44] H. Wang, H. Liu, Z. Dong, T. Song, W. Li et al., Size mismatch induces cation segregation in CsPbI_3_: forming energy level gradient and 3D/2D heterojunction promotes the efficiency of carbon-based perovskite solar cells to over 15%. Nano Energy **89**, 106411 (2021). 10.1016/j.nanoen.2021.106411

[45] X. Yang, D. Luo, Y. Xiang, L. Zhao, M. Anaya et al., Buried interfaces in halide perovskite photovoltaics. Adv. Mater. **33**, 2006435 (2021). 10.1002/adma.202006435 10.1002/adma.202006435 33393159

[46] P. Chen, W. Pan, S. Zhu, F. Cao, A. Tong et al., Buried modification with tetramethylammonium chloride to enhance the performance of perovskite solar cells with n-i-p structure. Chem. Eng. J. **468**, 143652 (2023). 10.1016/j.cej.2023.143652

[47] B. Yang, J. Keum, O.S. Ovchinnikova, A. Belianinov, S. Chen et al., Deciphering halogen competition in organometallic halide perovskite growth. J. Am. Chem. Soc. **138**, 5028–5035 (2016). 10.1021/jacs.5b13254 26931634 10.1021/jacs.5b13254

[48] S. Liu, J. Li, W. Xiao, R. Chen, Z. Sun et al., Buried interface molecular hybrid for inverted perovskite solar cells. Nature **632**, 536–542 (2024). 10.1038/s41586-024-07723-3 38925147 10.1038/s41586-024-07723-3

[49] A. Al-Ashouri, E. Köhnen, B. Li, A. Magomedov, H. Hempel et al., Monolithic perovskite/silicon tandem solar cell with >29% efficiency by enhanced hole extraction. Science **370**, 1300–1309 (2020). 10.1126/science.abd4016 33303611 10.1126/science.abd4016

[50] A. Al-Ashouri, A. Magomedov, M. Roß, M. Jošt, M. Talaikis et al., Conformal monolayer contacts with lossless interfaces for perovskite single junction and monolithic tandem solar cells. Energy Environ. Sci. **12**, 3356–3369 (2019). 10.1039/C9EE02268F

[51] M. Stolterfoht, P. Caprioglio, C.M. Wolff, J.A. Márquez, J. Nordmann et al., The impact of energy alignment and interfacial recombination on the internal and external open-circuit voltage of perovskite solar cells. Energy Environ. Sci. **12**, 2778–2788 (2019). 10.1039/C9EE02020A

[52] X. Luo, H. Luo, H. Li, R. Xia, X. Zheng et al., Efficient perovskite/silicon tandem solar cells on industrially compatible textured silicon. Adv. Mater. **35**, 2207883 (2023). 10.1002/adma.202207883 10.1002/adma.202207883 36599055

[53] F. Wang, D. Duan, Y. Sun, T. Wang, G. Yang et al., Uncovering chemical structure-dependency of ionic liquids as additives for efficient and durable perovskite photovoltaics. Nano Energy **125**, 109549 (2024). 10.1016/j.nanoen.2024.109549

[54] Z. Zhang, X. Tian, C. Wang, J. Jin, Y. Jiang et al., Revealing superoxide-induced degradation in lead-free tin perovskite solar cells. Energy Environ. Sci. **15**, 5274–5283 (2022). 10.1039/d2ee02796h

[55] Y. Shao, Z. Xiao, C. Bi, Y. Yuan, J. Huang, Origin and elimination of photocurrent hysteresis by fullerene passivation in CH_3_NH_3_PbI_3_ planar heterojunction solar cells. Nat. Commun. **5**, 5784 (2014). 10.1038/ncomms6784 25503258 10.1038/ncomms6784

[56] W.-J. Yin, T. Shi, Y. Yan, Unusual defect physics in CH_3_NH_3_PbI_3_ perovskite solar cell absorber. Appl. Phys. Lett. **104**, 063903 (2014). 10.1063/1.4864778

[57] M.I. Saidaminov, J. Kim, A. Jain, R. Quintero-Bermudez, H. Tan et al., Suppression of atomic vacancies *via* incorporation of isovalent small ions to increase the stability of halide perovskite solar cells in ambient air. Nat. Energy **3**, 648–654 (2018). 10.1038/s41560-018-0192-2

[58] J. Wu, J. Shi, Y. Li, H. Li, H. Wu et al., Quantifying the interface defect for the stability origin of perovskite solar cells. Adv. Energy Mater. **9**, 1901352 (2019). 10.1002/aenm.201901352

[59] J. Endres, D.A. Egger, M. Kulbak, R.A. Kerner, L. Zhao et al., Valence and conduction band densities of states of metal halide perovskites: a combined experimental-theoretical study. J. Phys. Chem. Lett. **7**, 2722–2729 (2016). 10.1021/acs.jpclett.6b00946 27364125 10.1021/acs.jpclett.6b00946 PMC4959026

[60] Z. Yang, Y. Wu, N. Yang, R. Yang, Y. Hao, Revealing the role of polyacrylonitrile for highly efficient and stable perovskite solar cells at extremely low temperatures. Adv. Energy Mater. **14**, 2400638 (2024). 10.1002/aenm.202400638

[61] J. Zhang, B. Yu, Y. Sun, H. Yu, Minimized energy loss at the buried interface of p-i-n perovskite solar cells *via* accelerating charge transfer and forming p–n homojunction. Adv. Energy Mater. **13**, 2300382 (2023). 10.1002/aenm.202300382

[62] Y. Chen, Y. Shen, W. Tang, Y. Wu, W. Luo et al., Ion compensation of buried interface enables highly efficient and stable inverted MA-free perovskite solar cells. Adv. Funct. Mater. **32**, 2206703 (2022). 10.1002/adfm.202206703

[63] J. Yao, T. Kirchartz, M.S. Vezie, M.A. Faist, W. Gong et al., Quantifying losses in open-circuit voltage in solution-processable solar cells. Phys. Rev. Appl. **4**, 014020 (2015). 10.1103/physrevapplied.4.014020

[64] S. Chen, X. Xiao, H. Gu, J. Huang, Iodine reduction for reproducible and high-performance perovskite solar cells and modules. Sci. Adv. **7**, eabe8130 (2021). 10.1126/sciadv.abe8130 33658200 10.1126/sciadv.abe8130 PMC7929519

[65] Y.-H. Lin, N. Sakai, P. Da, J. Wu, H.C. Sansom et al., A piperidinium salt stabilizes efficient metal-halide perovskite solar cells. Science **369**, 96–102 (2020). 10.1126/science.aba1628 32631893 10.1126/science.aba1628

[66] S. Chen, X. Dai, S. Xu, H. Jiao, L. Zhao et al., Stabilizing perovskite-substrate interfaces for high-performance perovskite modules. Science **373**, 902–907 (2021). 10.1126/science.abi6323 34413234 10.1126/science.abi6323

[67] C. Deger, S. Tan, K.N. Houk, Y. Yang, I. Yavuz, Lattice strain suppresses point defect formation in halide perovskites. Nano Res. **15**, 5746–5751 (2022). 10.1007/s12274-022-4141-9

[68] W. Nie, J.-C. Blancon, A.J. Neukirch, K. Appavoo, H. Tsai et al., Light-activated photocurrent degradation and self-healing in perovskite solar cells. Nat. Commun. **7**, 11574 (2016). 10.1038/ncomms11574 27181192 10.1038/ncomms11574 PMC4873646

[69] J. Sun, C. Shou, J. Sun, X. Wang, Z. Yang et al., NiO_*x*_-seeded self-assembled monolayers as highly hole-selective passivating contacts for efficient inverted perovskite solar cells. Sol. RRL **5**, 2100663 (2021). 10.1002/solr.202100663

[70] X. Li, Z. Ying, X. Wang, Y. Zeng, X. Yang et al., How to enable highly efficient and large-area fabrication on specific textures for monolithic perovskite/silicon tandem solar cells? Inf. Funct. Mater. **1**, 160–180 (2024). 10.1002/ifm2.18

[71] X. Wang, Z. Ying, J. Zheng, X. Li, Z. Zhang et al., Long-chain anionic surfactants enabling stable perovskite/silicon tandems with greatly suppressed stress corrosion. Nat. Commun. **14**, 2166 (2023). 10.1038/s41467-023-37877-z 37061510 10.1038/s41467-023-37877-z PMC10105702

