# Supplementary material for: Top-Down Dual-Interface Carrier Management for Highly Efficient and Stable Perovskite/Silicon Tandem Solar Cells
Source: Nanomicro Lett. 2025 Feb 11;17:141. doi: 10.1007/s40820-024-01631-x (PMC11813841; doi:10.1007/s40820-024-01631-x)
Supplement: Supplementary file 1 — Supplementary file1 (DOCX 10419 KB) [file 40820_2024_1631_MOESM1_ESM.docx]

Supporting Information for

**Top-Down Dual-Interface Carrier Management for Highly Efficient and Stable Perovskite/Silicon Tandem Solar Cells**

Xin Li^1,2^, Zhiqin Ying^1,^*, Shuo Li^3^, Lei Chen^3^, Meili Zhang^1,2^, Linhui Liu^1^, Xuchao Guo^1^, Jun Wu^1^, Yihan Sun^1^, Chuanxiao Xiao^1^, Yuheng Zeng^1^, Jian Wu^3^, Xi Yang^1,^*, Jichun Ye^1,^*

^1^Zhejiang Provincial Engineering Research Center of Energy Optoelectronic Materials and Devices, Ningbo Institute of Materials Technology and Engineering, Chinese Academy of Sciences, Ningbo 315201, P. R. China

^2^University of Chinese Academy of Sciences, No.19(A) Yuquan Road, Shijingshan District, Beijing 100049, P. R. China

^3^CSI Solar Technologies (JiaXing) Co., Ltd, No.325 Kanghe Road, Gaozhao Street, Xiuzhou Jiaxing, Zhejiang 314001, P. R. China

*Corresponding authors. E-mail: [yingzhiqin@nimte.ac.cn](mailto:yingzhiqin@nimte.ac.cn) (Zhiqin Ying); [yangx@nimte.ac.cn](mailto:yangx@nimte.ac.cn) (Xi Yang); [jichun.ye@nimte.ac.cn](mailto:jichun.ye@nimte.ac.cn) (Jichun Ye)

**S1 Experimental Section**

**S1.1 Synthesis of** **piperazinium chloride (PCl)**

Piperazine (3.74 g, 46 mmol) was dissolved in ethanol (30 mL) and placed in an ice-water bath. 38% hydrochloric acid solution in water (4.41 g, 46 mmol) was added dropwise and reacted for 30 minutes. The ethanol solvent was then rotary evaporated under reduced pressure, and the residual solid was washed with ethyl acetate for 5 times and subsequently dried for 12 hours to obtain PCl as white sheet-like crystal (4.20 g, 74.6%). ^1^H NMR (600 MHz, DMSO-*d*_6_): δ 7.17 (s, 2H), 2.91 (s, 8H), ^13^C NMR (400 MHz, DMSO-d6) δ 43.11 (Fig. S2).

**S1.2 Synthesis of** **piperazinium bromide (PBr)**

PBr was synthesized similar to PCl. Piperazine (3.62 g, 42 mmol) was dissolved in ethanol (30 mL) and placed in an ice-water bath. 48% hydrobromic acid solution in water (7.08 g, 42 mmol) was added dropwise and reacted for 30 minutes. The ethanol solvent was then rotary evaporated under reduced pressure, and the residual solid was washed with ethyl acetate for 5 times and subsequently dried for 12 hours to obtain PBr powders (6.12 g, 87.2%). ^1^H NMR (600 MHz, DMSO-*d*_6_): δ 6.74 (s, 2H), 2.92 (s, 8H), ^13^C NMR (400 MHz, DMSO-d6) δ 43.11 (Fig. S3).

**S1.3 DFT calculation**

***S1.3.1 ESP and dipole moment calculation***

The theoretical calculations were performed via the Gaussian 16 suite of program. The structures of the studied molecules (denoted by M^+^) were fully optimized at the B3LYP-D3BJ/def2-SVP level of theory. The vibrational frequencies of the optimized structures were carried out at the same level. The structures were characterized as a local energy minimum on the potential energy surface by verifying that all the vibrational frequencies were real. The dipole moment of the molecule was calculated with a larger basis set def2-TZVPD basis set. The Visual Molecular Dynamics (VMD) program was used to plot the color-filled iso-surface graphs to visualize the molecular electrostatic potential (MESP).

***S1.3.2 Formation energy of V_I_ calculation***

First-principles calculations based on density functional theory (DFT) were performed by using the Vienna Ab initio Simulation Package. The projector augmented wave (PAW) method was employed to describe the ion-electron interaction, and the generalized gradient approach (GGA) of the Perdew-Burke-Ernzerhof (PBE) functional was used to describe the electron-electron exchange correlation. The van der Waals interaction was also considered in this study by using DFT-D3 correction. The cutoff energy for the plane wave basis sets was defined as 450 eV. The 2×2 supercell of FAPbI_3_ associated with 3×3×1 Morkhost-Pack k-point grids were used. The geometry optimization was converged if the maximum force on each atom is less than 0.03 eV Å^−1^. A vacuum region of 15 Å was added along the z-direction to avoid interactions between slabs. The whole systems were fully relaxed, except that the bottom layer was fixed.

**S1.4 Calculation Methods**

***S1.4.1 Calculation of the quasi-fermi level splitting (QFLS) based on the PLQY***

To investigate the open-circuit voltage potential for every individual stack e.g., surface treatments and depositing C_60_ layer, the internal QFLS values of 1.68-eV bandgap perovskite films on a bare glass substrate and glass/ITO/MeO-2PACz substrates were calculated by PLQY measurements [S1]:

$$QFLS={QFLS}_{rad}+K_{B}T\cdot ln\left( PLQY \right)=K_{B}T\cdot\ln(PLQY\frac{J_{G}}{J_{0,rad}})$$

Here, QFLS is the difference between the electron and hole quasi-Fermi levels in the perovskite layer, K_B_ is the Boltzmann constant, and T is the temperature (300 K). *J*_G_ is the generated current density under illumination, in this case, approximated to the short-circuit current density *J*_SC_ (20.8 mA cm^−2^) of devices. *J*_0,rad_ is the dark radiative recombination saturation current density. According to the detailed balance at open-circuit conditions, the *J*_0,rad_ can be calculated by the following equations:

$$J_{0,rad}=e\int_{0}^{\infty} {EQE}_{PV}\left( E \right)\emptyset_{BB}\left( E \right)dE$$

$$\emptyset_{BB}\left( E \right)=\frac{2\pi E^{2}}{h^{3}c^{2}}\frac{1}{\exp\left( \frac{E}{k_{B}T} \right)-1}$$

External quantum efficiency (EQE) of the p-i-n PSCs and the emitted spectral photon flux are calculated when the device is in equilibrium with the black-body radiation (T = 300 K). E is the photo energy, h is the Plank constant, and c is the light speed in vacuum. Based on the above equations, the *J*_0,rad_ was calculated similarly as 2.656 × 10^−23^ A m^−2^ for all systems independent of the surface treatment. Subsequently, combined with the PLQY value of the samples, the QFLS of the samples can be obtained.

***S1.4.2 Trap density of states (tDOS) measurement***

The tDOS can be deduced pursuant to the following equations:

$$N_{t}\left( E_{\omega} \right)=-\frac{V_{bi}}{eW}\frac{\omega}{K_{b}T}\frac{dC}{dW}$$

$$E_{\omega}=K_{b}Tln(\frac{\beta T^{2}}{\omega})$$

where *V_bi_* is the built-in potential, e is the elementary charge, W is the depletion width, ω is the frequency and C is the capacitance. The *V*_bi_ and W can be inferred from the Mott–Schottky analysis [S2].

***S1.4.3 Calculation of interface defect density (N_SS_)***

An expanded equivalent circuit model and corresponding admittance spectroscopy analysis was introduced as follows:


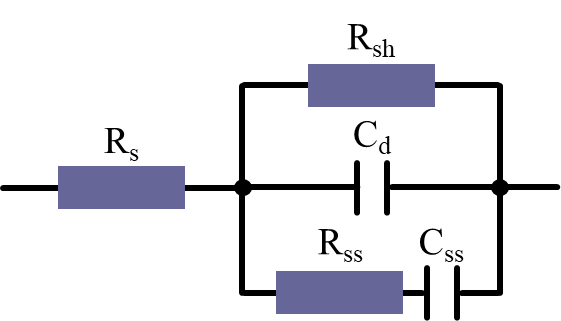


Where R_SS_ is the resistance that a charge needs before being captured by the interface defect, C_SS_ reflects the interface defects response. The relationship between C_SS_ and the interface defect density (N_SS_) is:

$$C_{SS}=qAN_{SS}$$

where q is the elementary charge and A is the device active area (0.16 cm^2^). With this theoretical model, the experimental admittance measurements can be fitted by using the following formula:

$$\frac{1}{\omega}\left( G-\frac{1}{R_{sh}} \right)=\frac{\omega R_{SS}C_{SS}^{2}}{1+{(\omega R_{SS}C_{SS})}^{2}}$$

Where G is the admittance and ω is the angular frequency. In this case, the C_SS_ and the N_SS_ can be then calculated from the admittance spectra.

***S1.4.4 Calculation of V_OC_ loss induced by non-radiative recombination (∆V_OC,nr_)***

In a real perovskite solar cell, additional non-radiative recombination loss should be considered according to the following equation:

$$V_{OC}=V_{OC,rad}-\Delta V_{OC,nr}=V_{OC,rad}+\frac{K_{B}T}{q}ln({EQE}_{EL})$$

in which ∆*V*_OC,nr_ refers to the non-radiative recombination loss, and EQE_EL_ refers to the external quantum efficiency of electroluminescence under an injection current density equal to *J*_SC_.

***S1.4.5 FF deficit analysis***

The FF deficit in devices has considerable contributions from two main reasons: the trap-assisted non-radiative recombination loss and charge transport loss. By deducing the maximum FF (FF_max_), according to the following empirical equation, the above two losses could be separately quantified:

${FF}_{max}=\frac{\upsilon_{OC}-ln(\upsilon_{OC}+0.72)}{\upsilon_{OC}+1}$,

where

$\upsilon_{OC}=\frac{V_{OC}}{nK_{B}T/q}$.

The ideality factors (n) were extracted from the *V*_OC_ as a function of light intensity on a logarithmic scale, which were shown in Figure 4g. Accordingly, the trap-assisted non-radiative recombination loss and charge transport loss can be distinguished and quantified.

***S1.4.6 Residual stress measurement***

The macro-residual stress (σ) within perovskite films can be deduced by fitting 2θ as a function of sin^2^ψ according to the following equation:

$$\sigma=-\frac{E}{2(1+)}\frac{\pi}{180}\cot\theta_{0}\frac{(2\theta)}{{sin}^{2}}$$

Where σ is the in-plane macro-residual stress within the perovskite film, Ε and υ are the Young’s modulus and the Poisson’s ratio, respectively. θ_0_ is the diffraction peak for a strain-free perovskite crystal plane. θ is the corresponding diffraction peak under different tilt angles (ψ). By fitting 2θ as a function of sin^2^ψ, σ can be deduced from the slope of the fitting line.

**S2 Supplementary Figures and Tables**

**Fig. S1** *J–V* curves of champion devices treated with 0 (control), 0.1, 0.15, 0.3 and 0.5 mg mL^−1^ of PCl


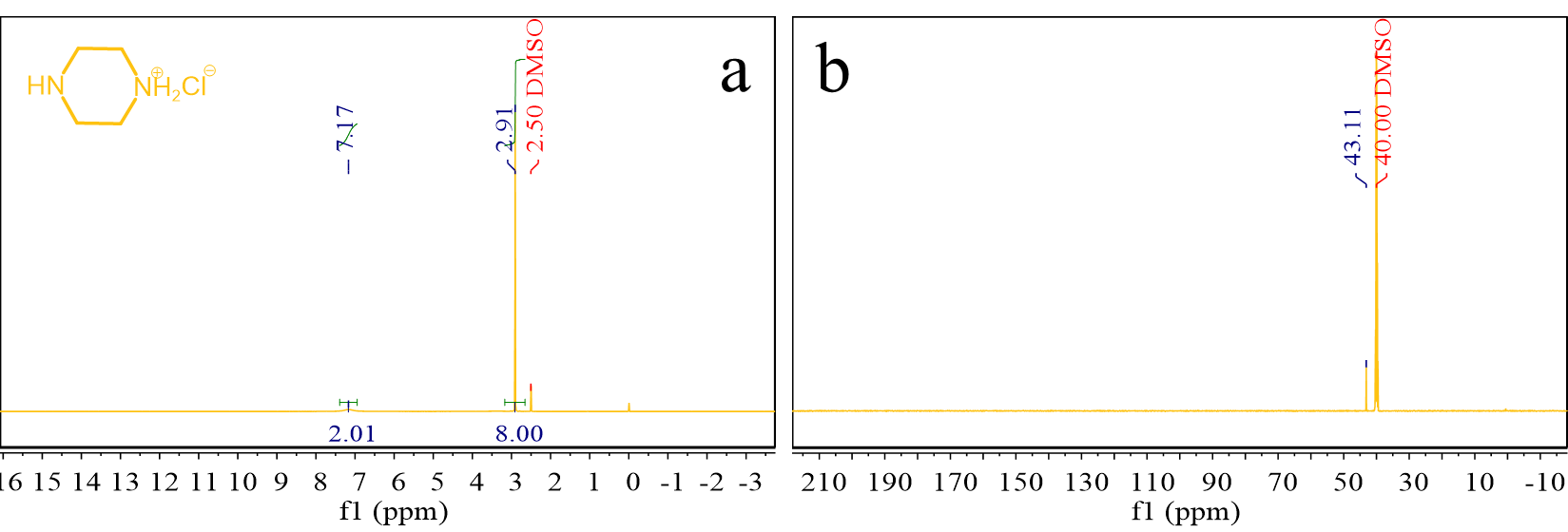


**Fig. S2** Characterisation of PCl after synthesis. a) ^1^H NMR in DMSO-d_6_ (signal at 2.50 ppm corresponds to the DMSO solvent). b) ^13^C NMR DMSO-d_6_ (signal at 40.00 ppm corresponds to the DMSO solvent)


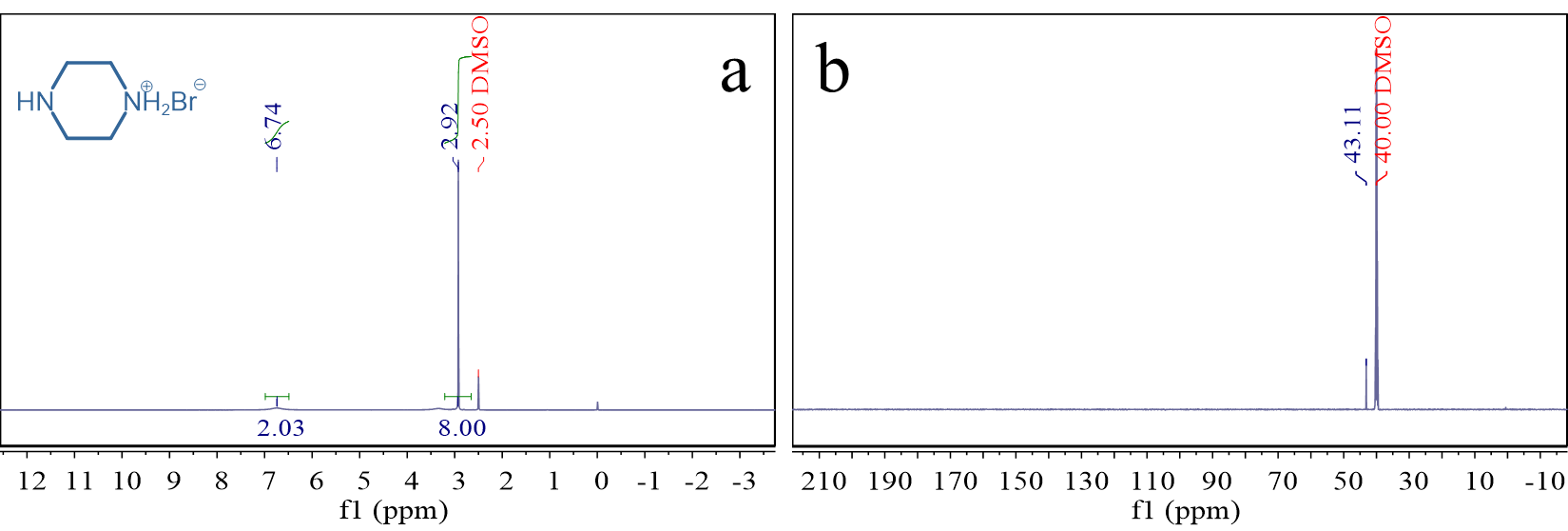


**Fig. S3** Characterisation of PBr after synthesis. a) ^1^H NMR in DMSO-d_6_ (signal at 2.50 ppm corresponds to the DMSO solvent). b) ^13^C NMR DMSO-d_6_ (signal at 40.00 ppm corresponds to the DMSO solvent)


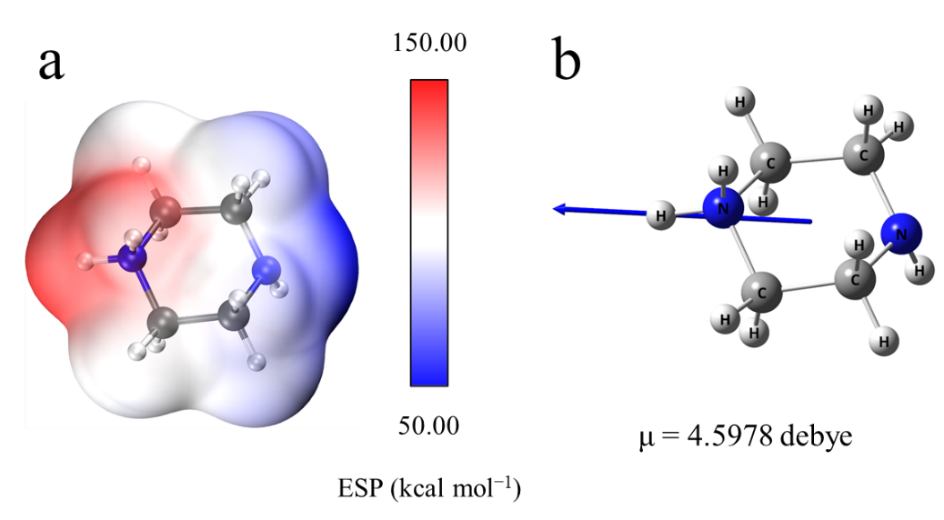


**Fig. S4** The distribution of a) electrostatic potential (ESP) and b) dipole moment (μ) of piperazinium cation


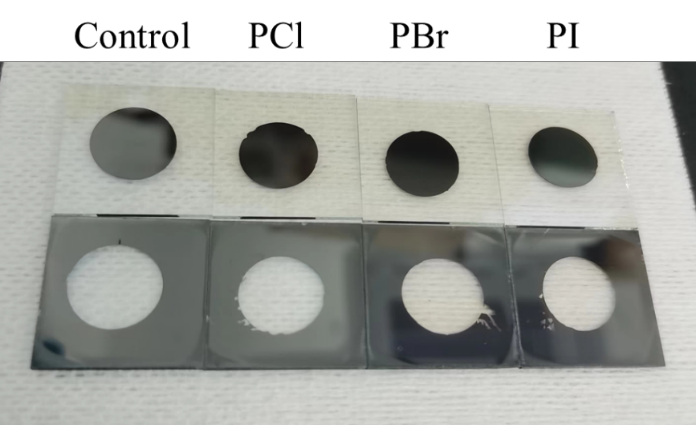


**Fig. S5** The photographys of perovskite films for buried interface characterization. The bottom surface samples of perovskite films were fabricated with the following method: the upper surface of the prepared perovskite film was partly pasted together with a ITO glass through UV glue. After the glue is solidified, the film was peeled off from the ITO/MeO-2PACz substrate, then the bottom surface of perovskite film for characterization was got. Notably, unless otherwise stated, all samples for buried interface characterization are peeled off from the ITO/MeO-2PACz substrate


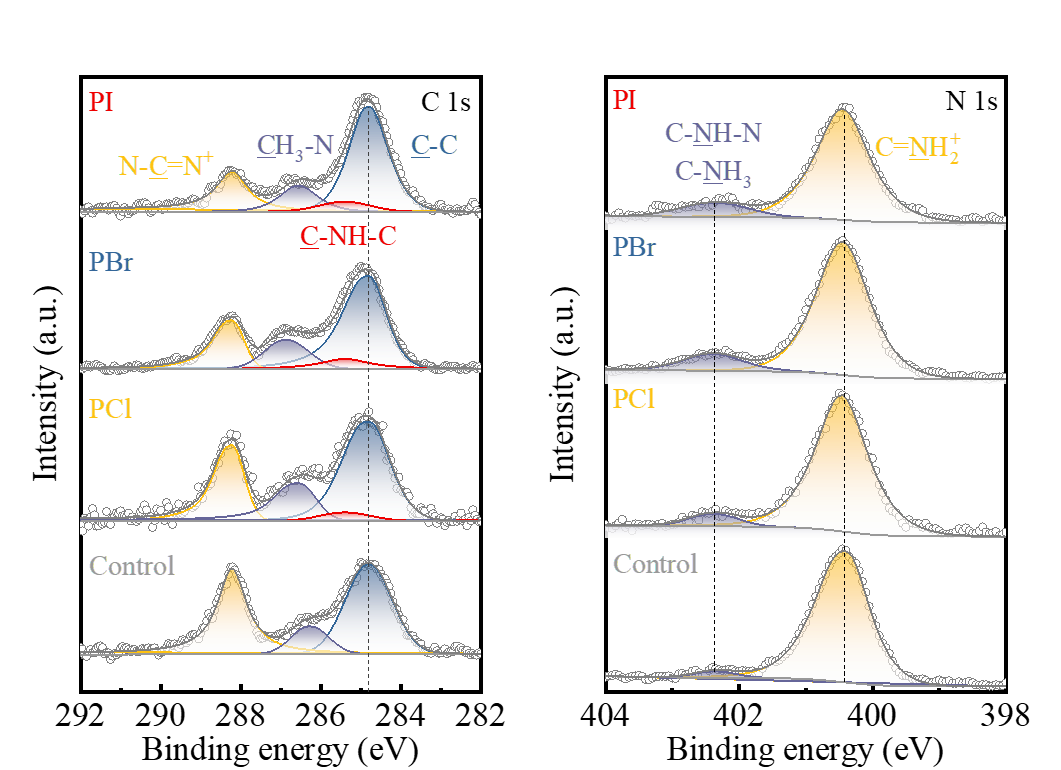


**Fig. S6** C 1s and N 1s XPS spectra of the upper surfaces for control, PCl, PBr and PI films

**Fig. S7** N 1s XPS spectra of PCl, PBr and PI powders

**
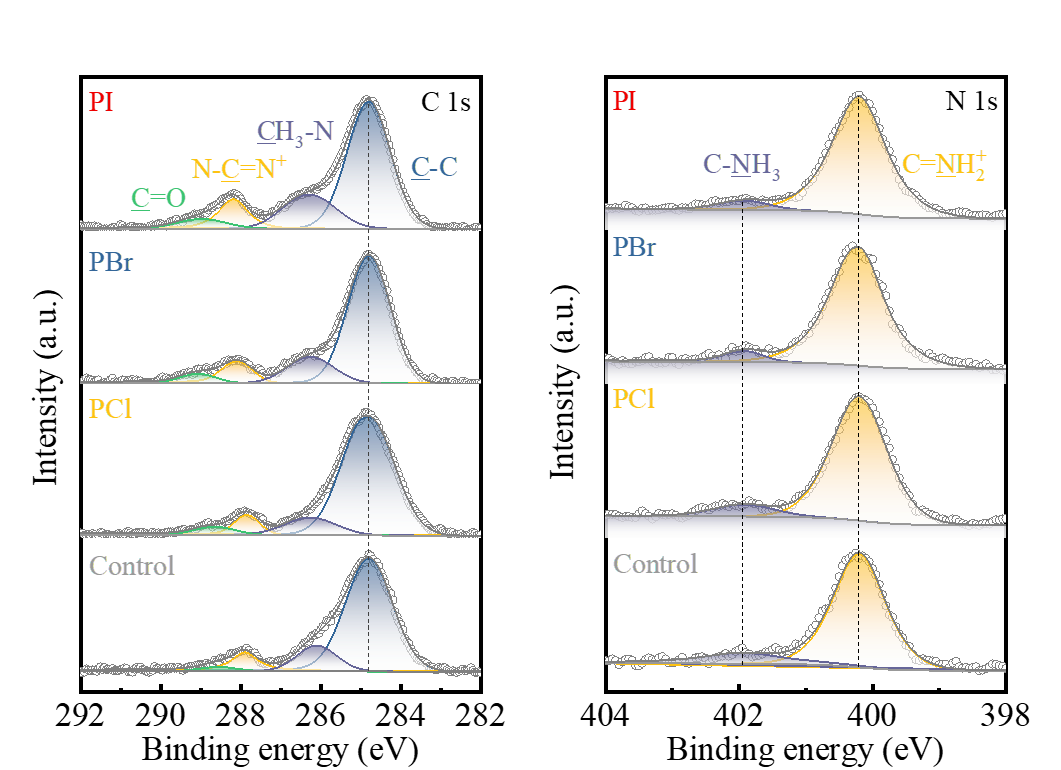
**

**Fig. S8** C 1s and N 1s XPS spectra of the buried interfaces for control, PCl, PBr and PI films


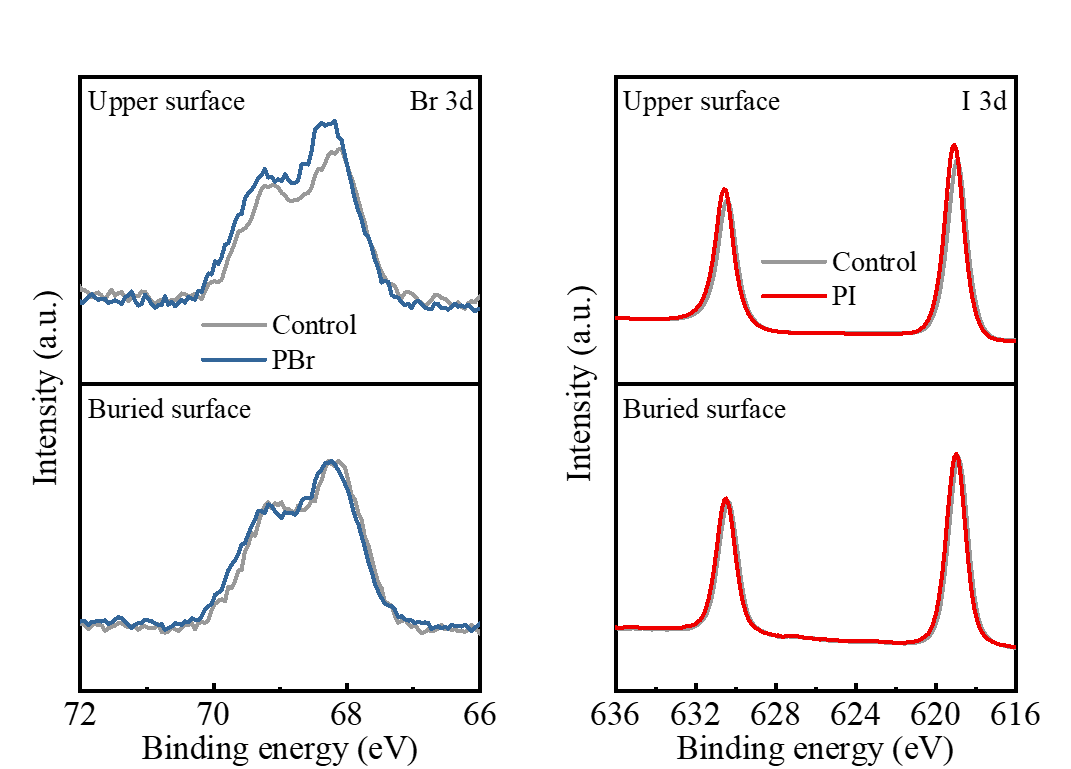


**Fig. S9** Br 3d and I 3d XPS spectra of the PBr and PI films


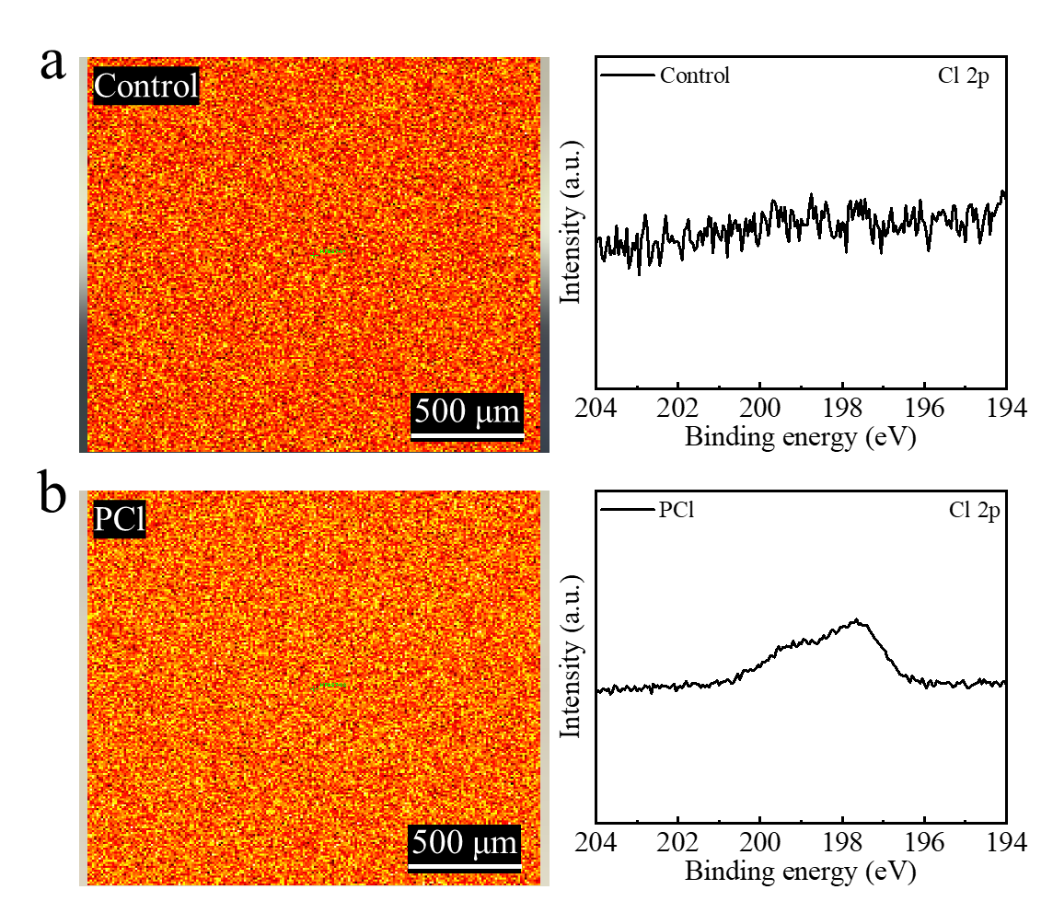


**Fig. S10** XPS mapping images and corresponding fine spectra of Cl 2p at the buried interface for the (a) control and (b) PCl-modified perovskite films. In comparison with the control film, XPS mapping reveals a greater Cl intensity at the buried interface for the PCl-modified perovskite film, which is consistent with the fine spectra of Cl 2p extracted from the XPS images. These results intuitively and collectively confirm the diffusion of Cl to the buried interface upon post-treatment

**Fi. S11** Cl 2p XPS spectra of the buried interface for the control and PCl-treated perovskite deposited on NiO_X_/MeO-2PACz substrate. No visible Cl signal is detected at the buried interface in the control film, but apparent Cl peaks are probed in the PCl case, which indicates the accumulation of Cl at the buried interface regardless of the introduction of NiO_X_ HTL

**
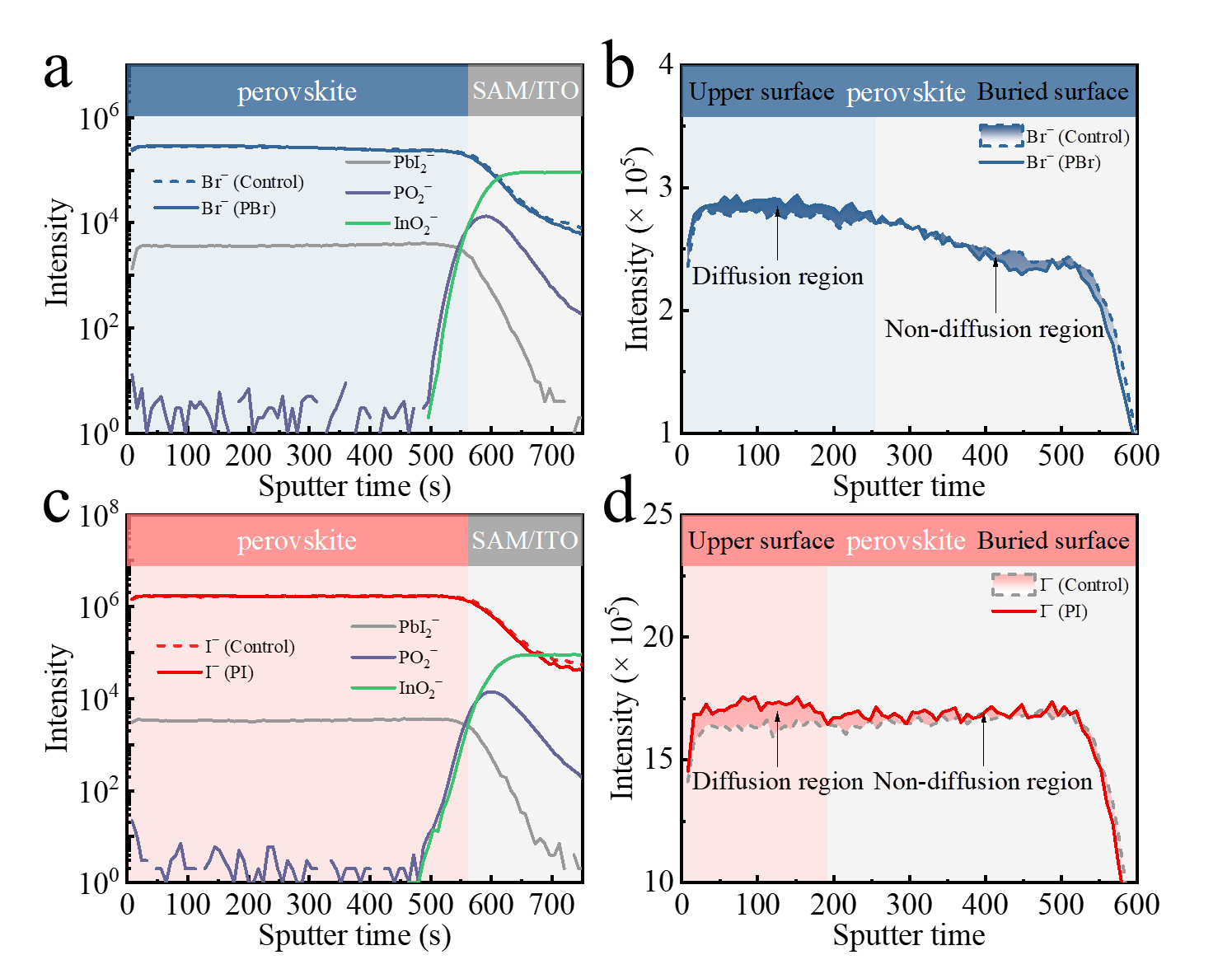
**

**Fig. S12 a,b**) ToF-SIMS depth profiles for the PBr-treated perovskite film deposited on ITO/MeO-2PACz substrate. **c,d**) ToF-SIMS depth profiles for the PI-treated perovskite film deposited on ITO/MeO-2PACz substrate.

Notably, no significantly higher signals of Br^−^ and I^−^ ions from PBr and PI films are detected on the upper surface compared to the control film, possibly due to the low concentration of PBr and PI (only 1.2 mmol mL^−1^) and the high noise level caused by the occurrence of Br^−^ and I^−^ ions inherent to the perovskite itself [S3, S4]. The two films appear to exhibit uniformly consistent distributions of Br^−^ and I^−^ signals with the control film throughout the entire perovskite films. To differentiate the Br^−^ and I^−^ ionic signals from PBr and PI treatments from those originating in the perovskite itself, the depth profiles of Br^−^ and I^−^ ions were individually plotted using linear coordinates[5], as shown in Fig. S12b, d.

**Fig. S13** ^1^H nuclear magnetic resonance (NMR) spectra of PX (X = Cl, Br, I) and corresponding mixtures of PX (X = Cl, Br, I)/PbI_2_.

The chemical shift of the hydrogen peak position of the -NH_2_^+^- group in the piperazinium cation gradually increase from 6.67 ppm for PI to 6.74 and 7.17 ppm for PBr and PCl, respectively, indicating a decrease in the shielding effect due to the substitution of Br^−^ and Cl^−^ anions [S6]. Since Cl^−^ has a higher electron-withdrawing ability than I^−^ and Br^−^, the electrostatic interaction between -NH_2_^+^- and Cl^−^ reduces the electron cloud density of hydrogen in the amino group [S7]. After mixing PbI_2_ with PBr and PI in solvent, a slight offset in the chemical shift of the hydrogen peak associated with -NH_2_^+^- is observed, indicating a weak ion exchange reaction of PBr and PI with PbI_2_. However, after adding PbI_2_ into the PCl solution, the chemical shift of the hydrogen peak associated with -NH_2_^+^- dramatically down-shifted from 7.17 ppm to 6.84 ppm, substantiating a stronger ion exchange of PCl with PbI_2_ compared to PBr and PI.

**Fig. S14** FTIR spectra of the pristine perovskite film and PX (X = Cl, Br, I) powders.

In order to investigate the distribution of piperazinium cation on the perovskite upper surface, AFM-IR was employed in this work. A feature peak at 1461 cm^−1^ was selected for AFM-IR measurements to exclusively display piperazinium cations on the perovskite film surface.

**
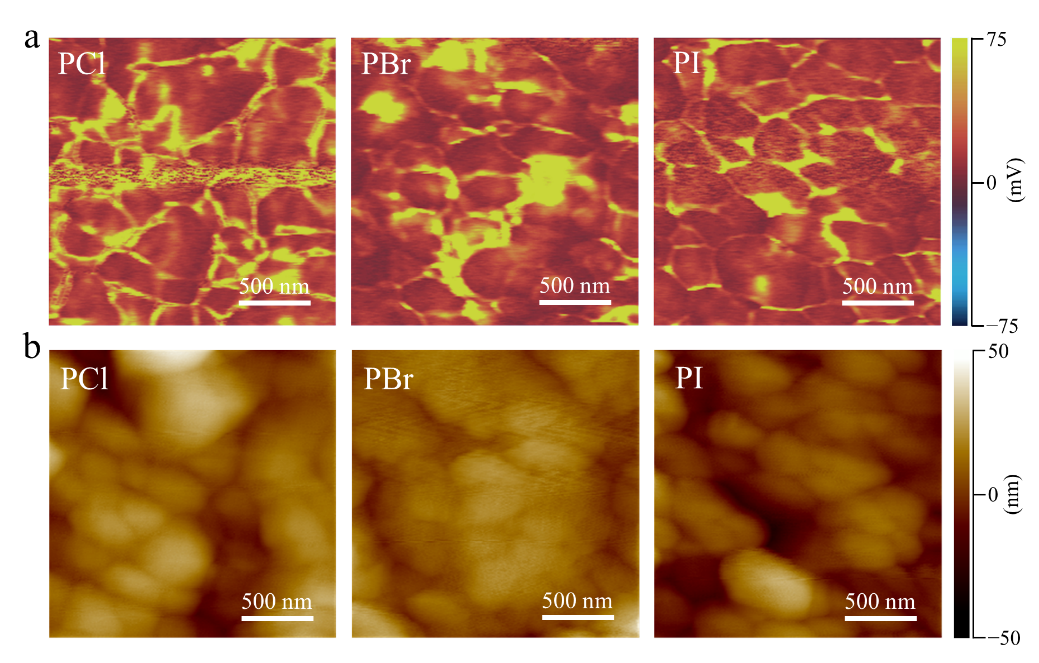
**

**Fig. S15** **a**) IR images and corresponding **b**) AFM topography images of piperazinium cation in PCl, PBr and PI films.

For IR images, the yellow regions correspond to the 1461 cm^−1^ feature peak signal of piperazinium cation. The piperazinium cation signals were mainly detected at the grain boundaries for all samples, implying that grain boundaries rather than grain surfaces are seriously defective [S8], but are successfully healed by piperazinium cations.


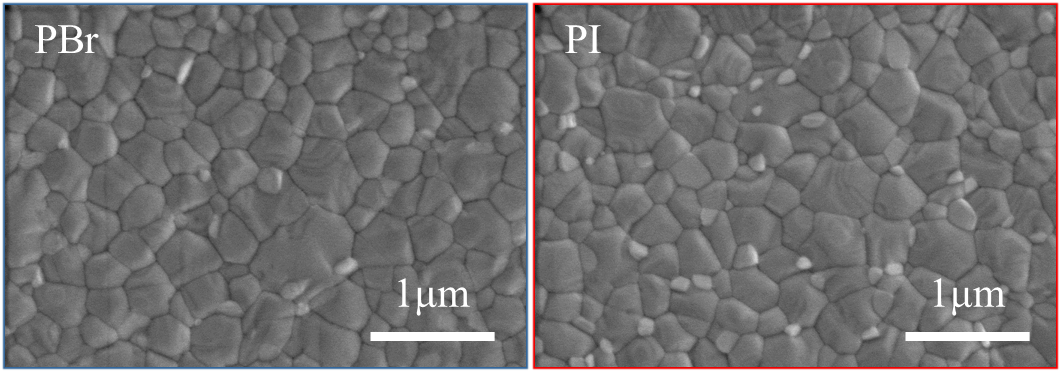


**Fig. S16** Top-view SEM images of the top surface for PBr and PI perovskite films

**Fig. S17** I 3d XPS spectra of upper surfaces for control, PCl, PBr and PI perovskite films

**
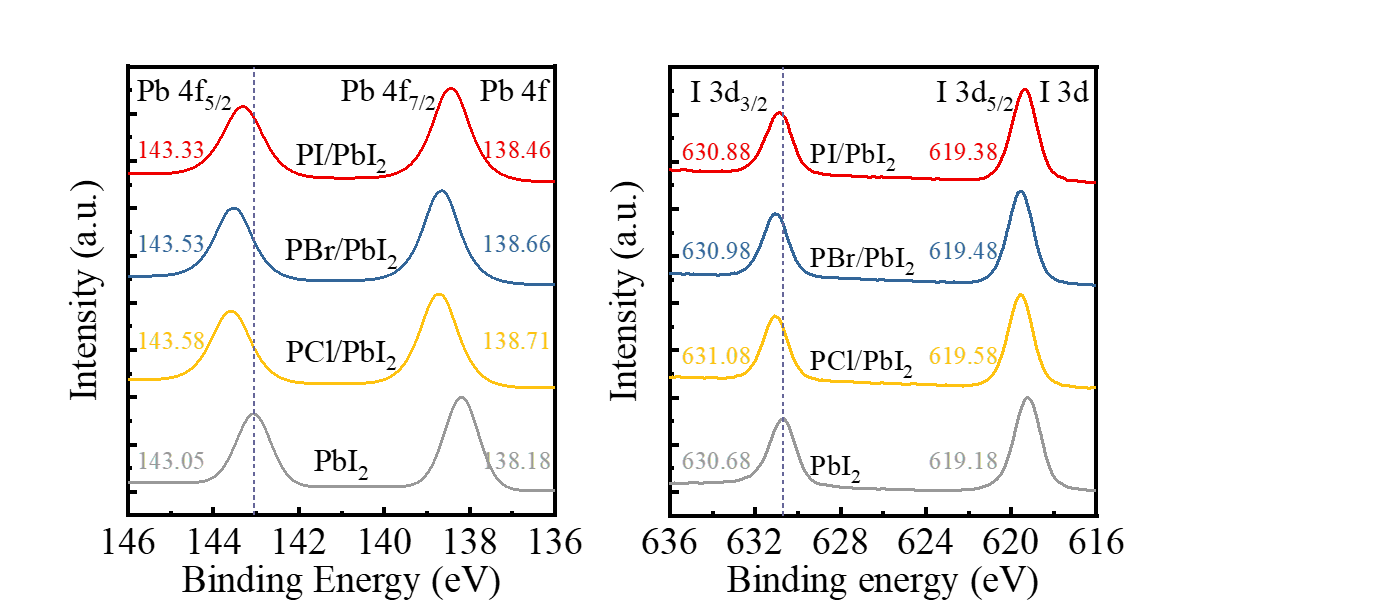
**

**Fig. S18** Pb 4f and I 3d XPS spectra of the original PbI_2_, PCl/PbI_2_, PBr/PbI_2_, and PI/PbI_2_ films

To prove that PCl is more reactive with PbI_2_, we used X-ray photoelectron spectroscopy (XPS) to evaluate the chemical interactions of the original PbI_2_, PCl/PbI_2_, PBr/PbI_2_, and PI/PbI_2_ films. As shown in the Pb 4f XPS spectra and Table S5, the Pb 4f spectrum of the control PbI_2_ film exhibits two prominent peaks at 143.05 and 138.18 eV, corresponding to Pb 4f_5/2_ and Pb 4f_7/2_, respectively. In the PbI_2_ films treated by PI, PBr and PCl, the binding energies of the two Pb 4f peaks were measured at 143.33 and 138.46 eV for PI-treated, 143.53 and 138.66 eV for PBr-treated, and 143.58 and 138.71 eV for PCl-treated PbI_2_ films. The gradually increased binding energies of the Pb 4f peaks from the PI- to PCl-treated PbI_2_ films confirm the progressively enhanced defect passivation ability. Notably, the I 3d peak of the PbI_2_ films treated with PI, PBr and PCl shifted to a higher binding energy compared to that of the control PbI_2_ film, which may be attributed to the incorporation of Cl^−^ inducing changes in the overlapping density of the Pb−I electron cloud [S9].


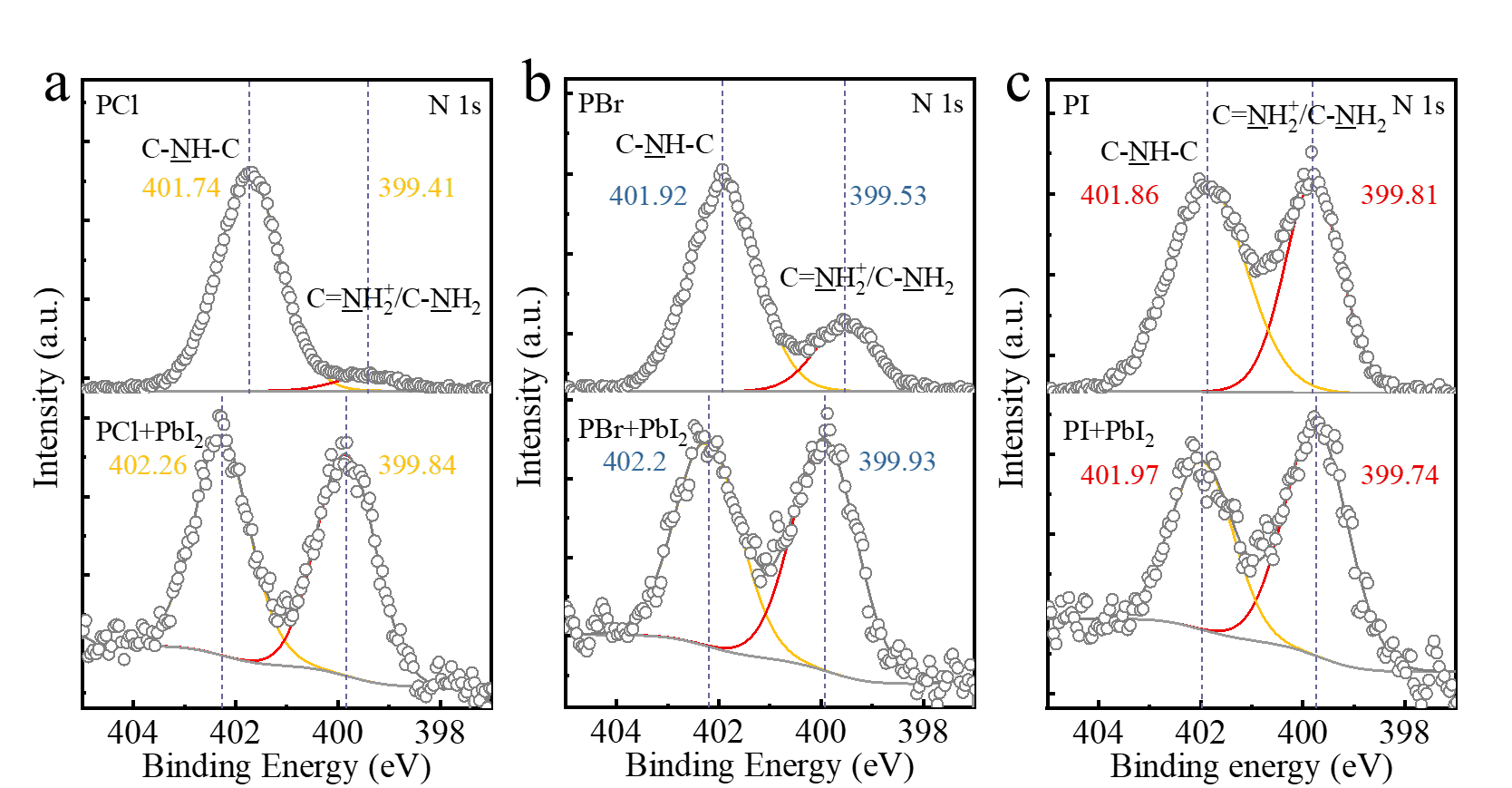


**Fig. S19** N 1s XPS spectra of **a**) PCl powders and PCl/PbI_2_ film, **b**) PBr powders and PBr/PbI_2_ film and **c**) PI powders and PI/PbI_2_ film

In comparison with PI-treated and PBr-treated PbI_2_ films, the N 1s of PbI_2_ film treated with PCl exhibits greater binding energy shifts, indicating that the chemical environment of piperazinium cations is altered by Cl^−^ anions [S10, S11], making it easier to bond with undercoordinated Pb^2+^.


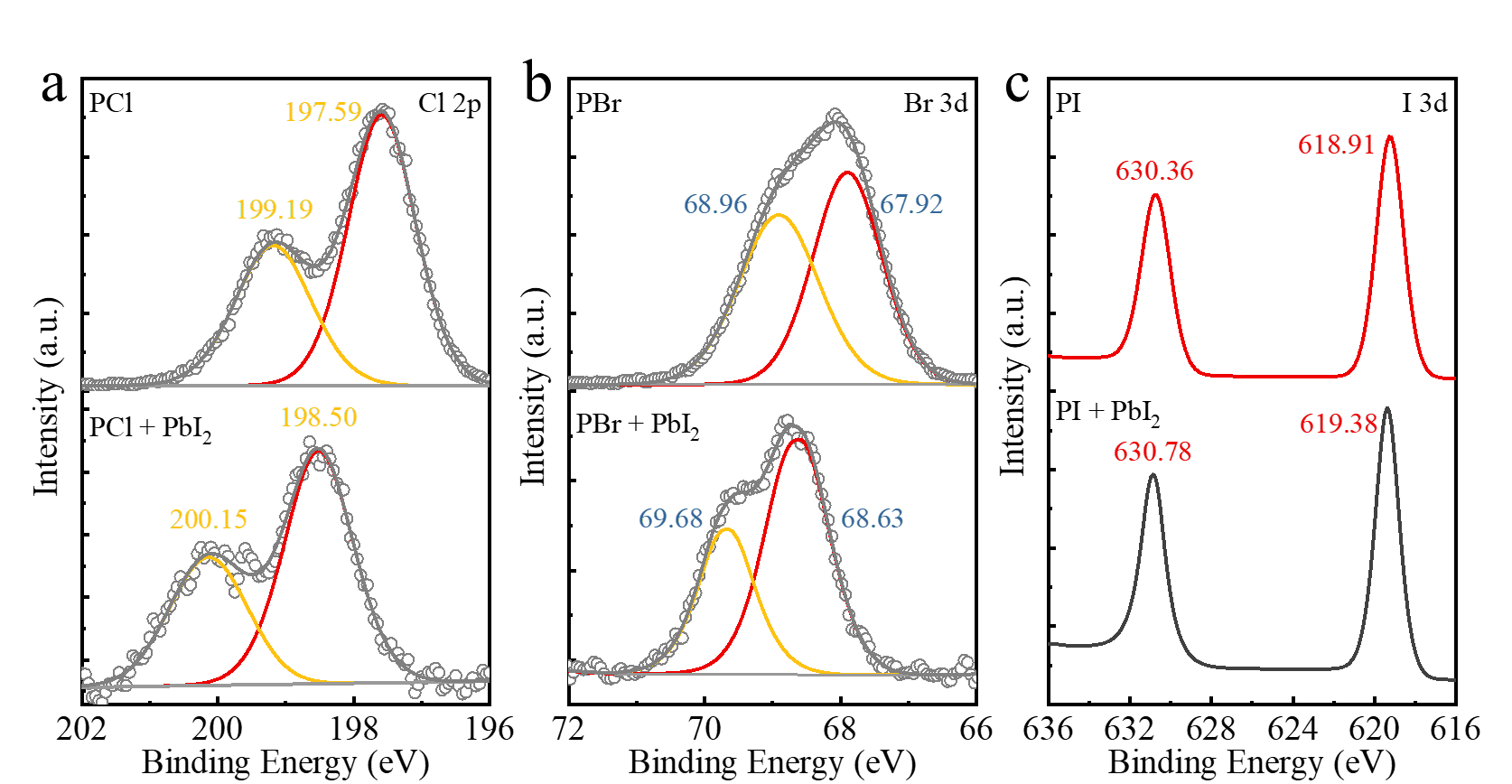


**Fig. S20** **a**) Cl 2p XPS spectra of PCl powders and PCl/PbI_2_ film; **b**) Br 3d XPS spectra of PBr powders and PBr/PbI_2_ film; **c**) I 3d XPS spectra of PI powders and PI/PbI_2_ film. The larger binding energy offset for Cl^−^ anions compared to Br^−^ and I^−^ anions suggests a stronger ionic bonding of Pb―Cl than Pb―Br and Pb―I [S12, S13], which contributes to a better passivation effect [S5].

**
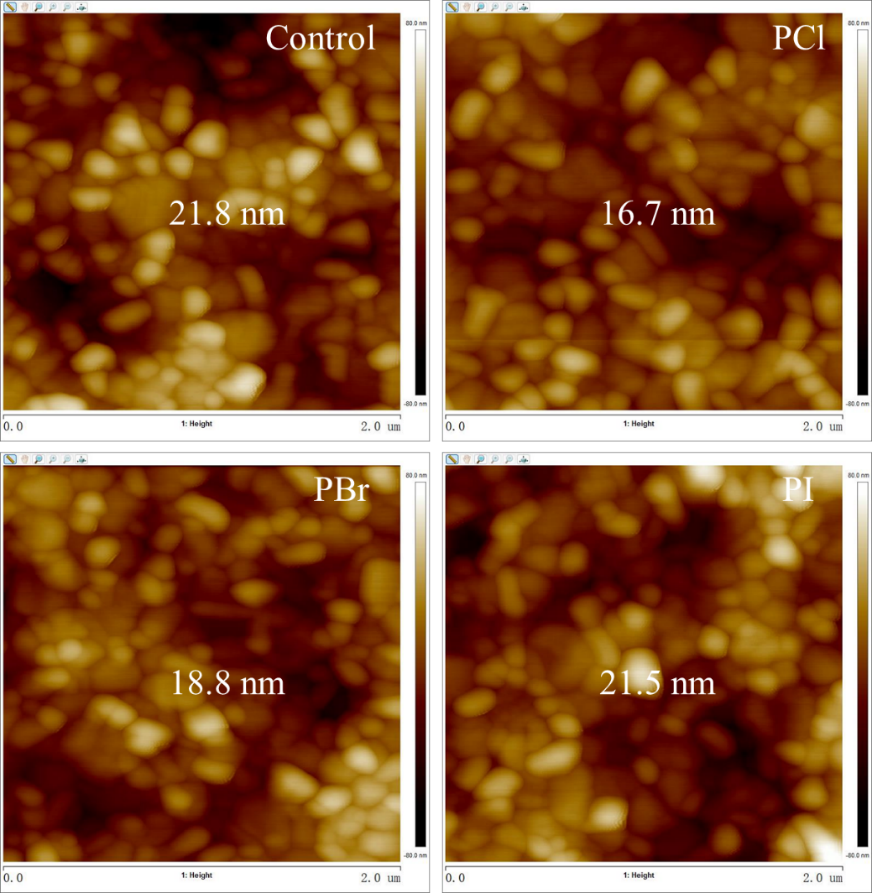
**

**Fig. S21** Atomic force microscopy (AFM) images of control, PCl, PBr and PI perovskite films. The root mean square surface roughness was marked in the images

**
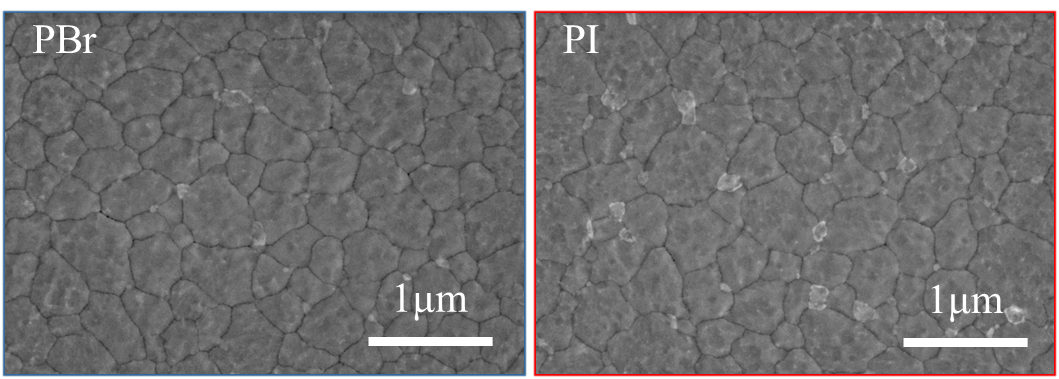
**

**Fig. S22** Top-view SEM images of the buried interface for PBr and PI perovskite films

**Fig. S23** I 3d XPS spectra of buried interfaces for control, PCl, PBr and PI films

**
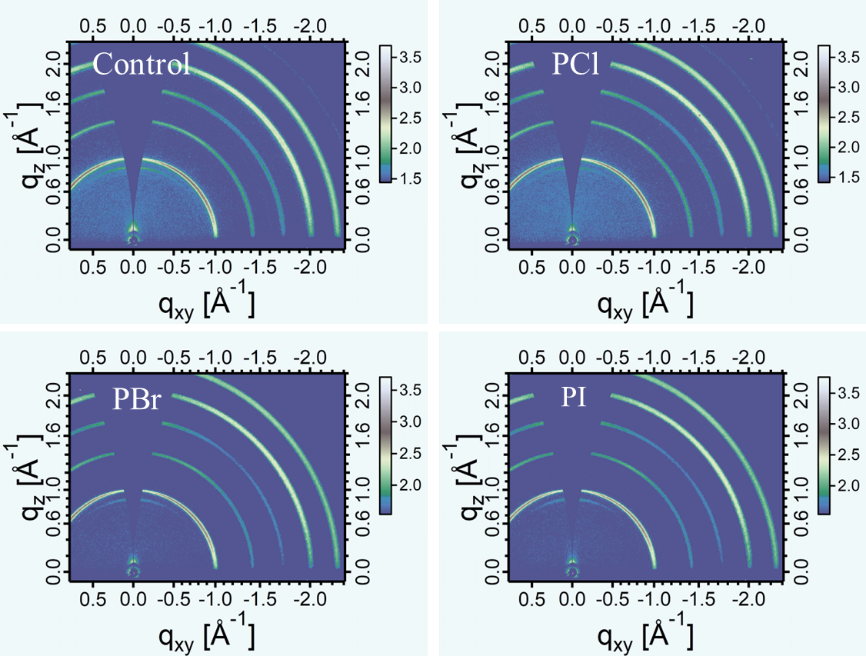
**

**Fig. S24** Grazing incident wide-angle X-ray scattering (GIWAXS) patterns of **a**) control, **b**) PCl-treated, **c**) PBr-treated and (**d**) PI-treated perovskite films. The incidence angle is 0.2°

**Fig. S25** The (001) out-of-plane line cuts of GIWAXS images of the control, PCl, PBr, and PI perovskite films

The peak area ratio of perovskite (001)/PbI_2_ for the control, PCl, PBr and PI films derived from the (001) out-of-plane line cuts of GIWAXS images (Fig. S24) are 4.31, 7.48, 5.36 and 5.28, respectively, which implies that the crystal crystallinity of perovskite films at the top region is gradually improved. Additionally, excessive PbI_2_ in the position at |q| = 0.88 Å^−1^ gradually decreases after PI, PBr, and PCl treatment. This result is consistent with the measurements from SEM images. The PbI_2_ signal that gradually decreases after PI, PBr, and PCl treatment indicates stronger interaction of PCl with PbI_2._

.**
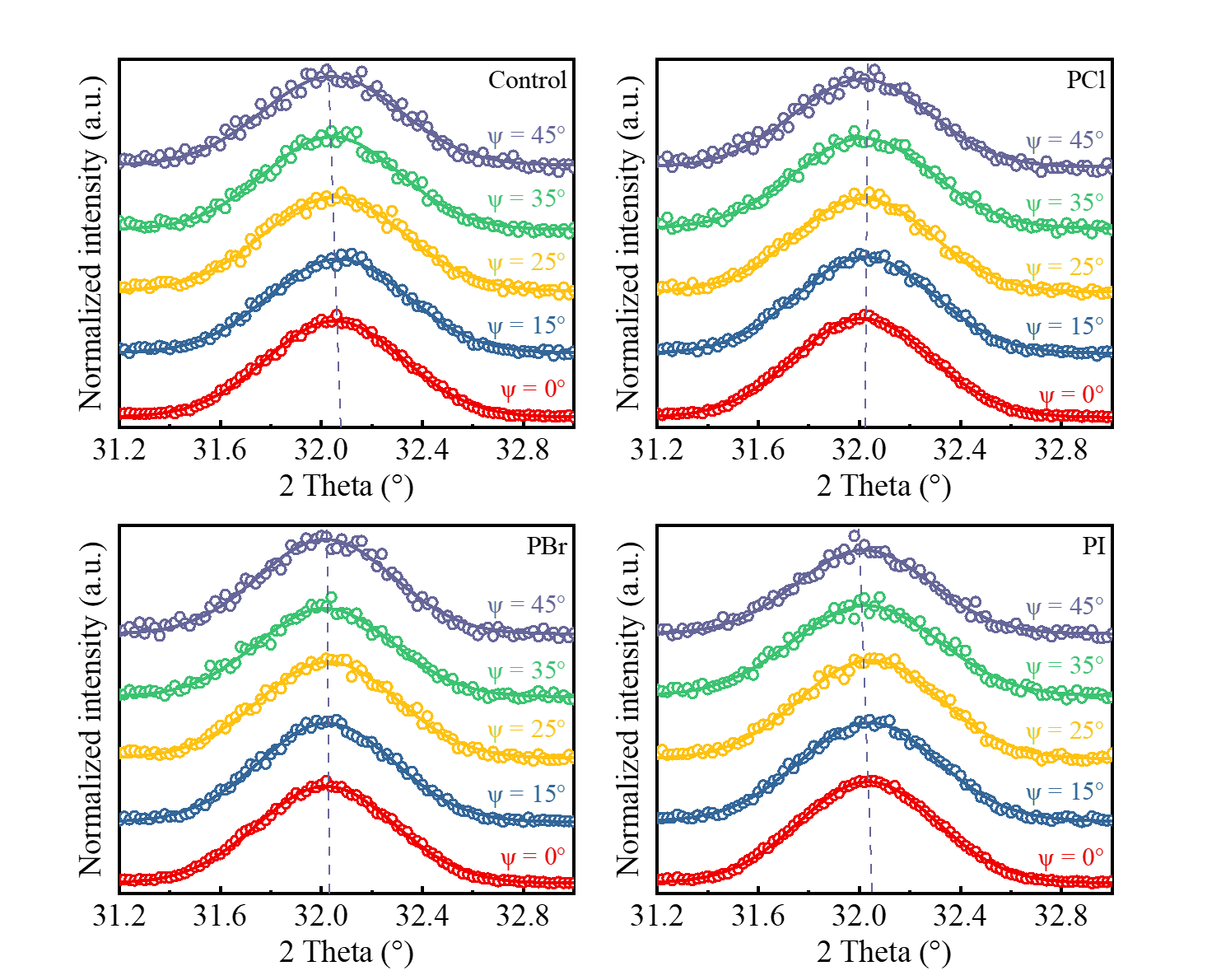
**

**Fig. S26** GIXRD patterns with different tilt angles for a) control, b) PCl, c) PBr and d) PI perovskite films

**Fig. S27** The C–F profiles of the control, PCl, PBr and PI devices

**
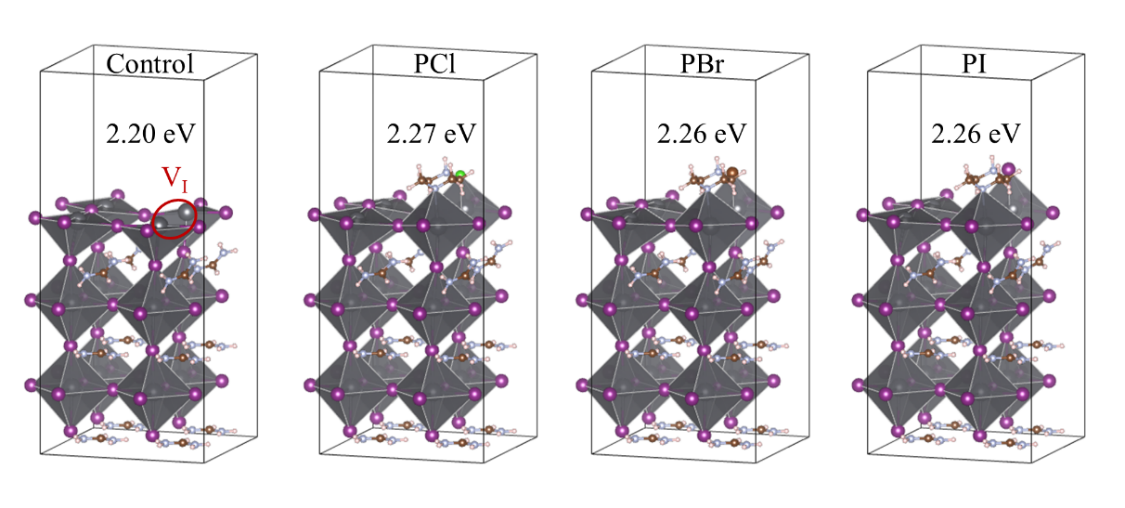
**

**Fig. S28** The formation energy of V_I_ (PbI_2_-terminated surface) in control, PCl, PBr and PI perovskite surface

**
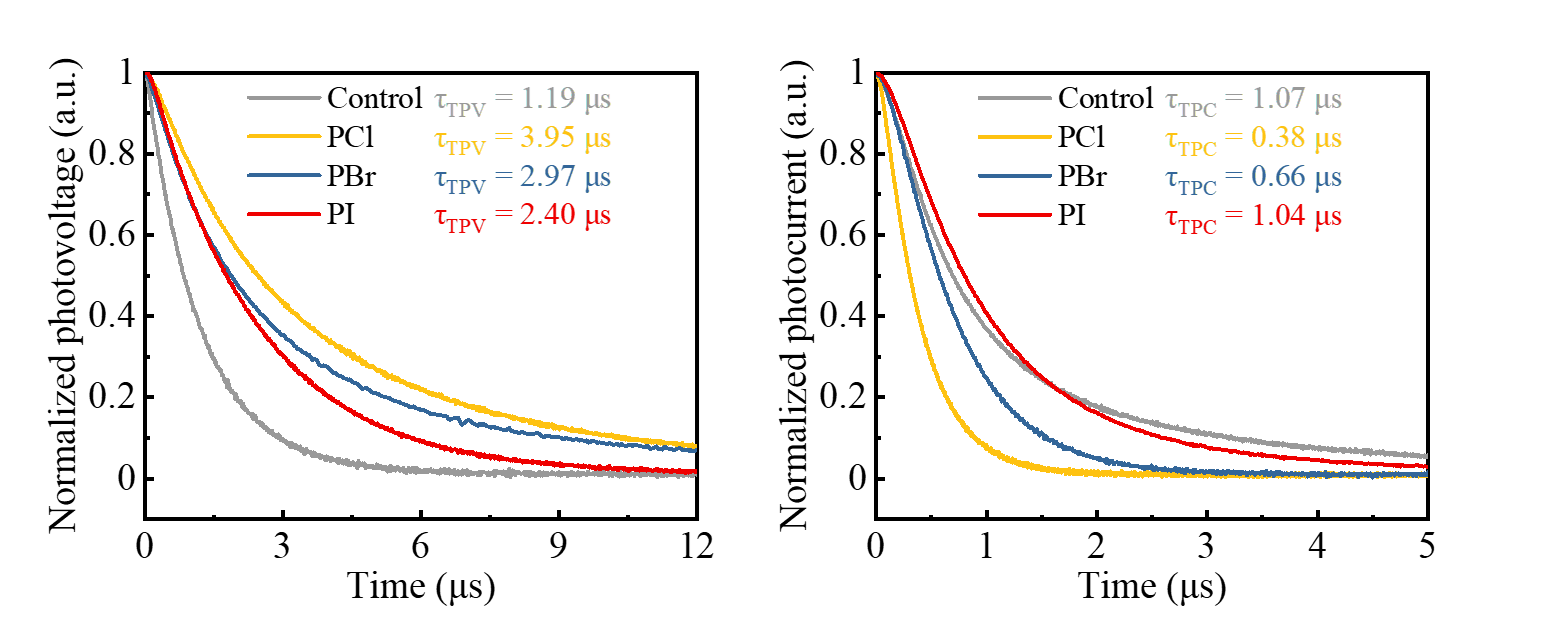
**

**Fig. S29 a**) Transient photovoltage (TPV), **b**) Transient photocurrent (TPC) decay curves of control, PCl, PBr and PI devices


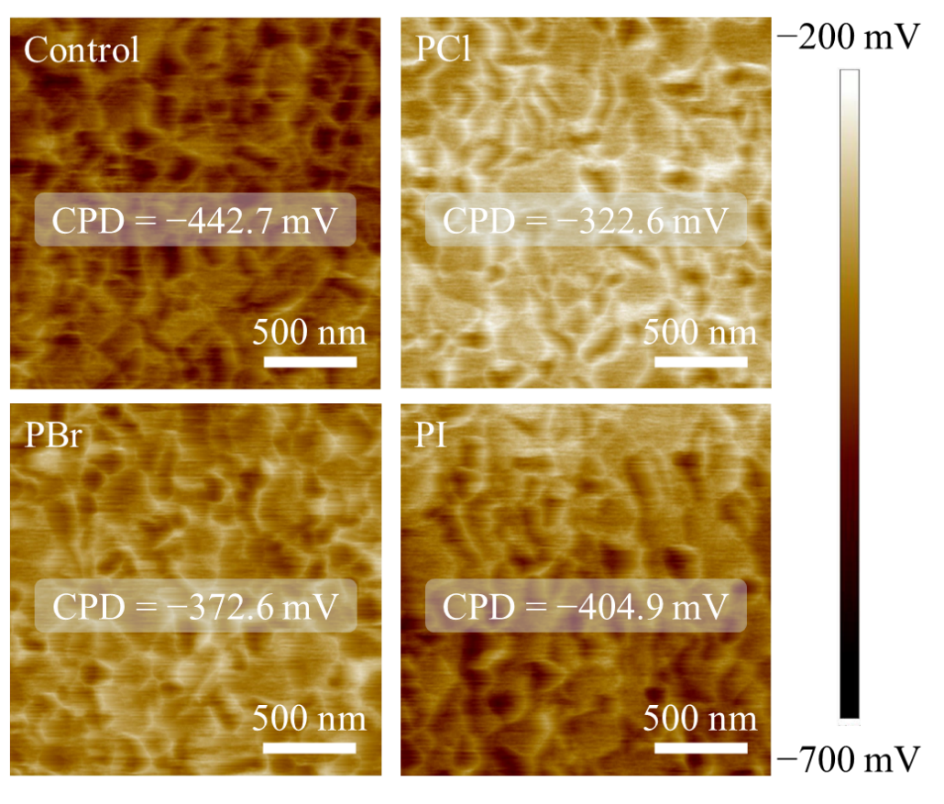


**Fig. S30** KPFM images of the top surface for the control, PCl, PBr and PI perovskites


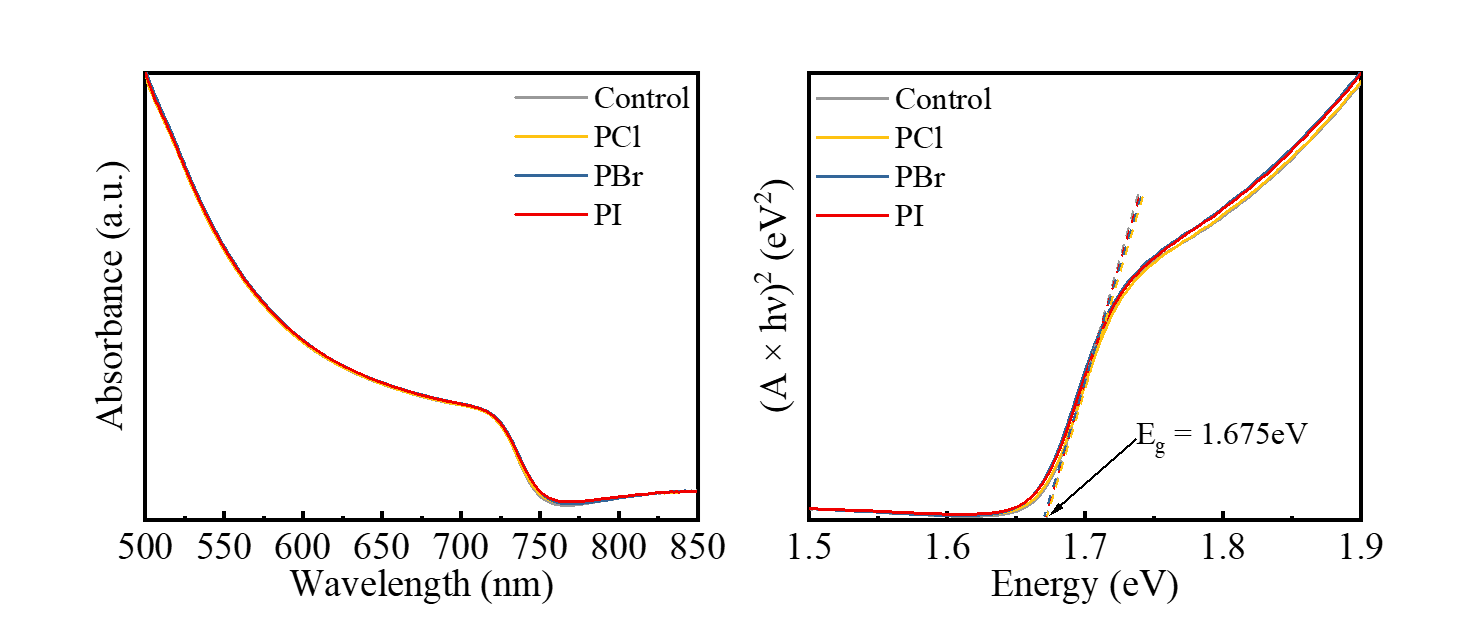


**Fig. S31** **a**) UV–vis absorption spectra and corresponding **b**) Tauc plots of control, PCl, PBr and PI perovskite films

**
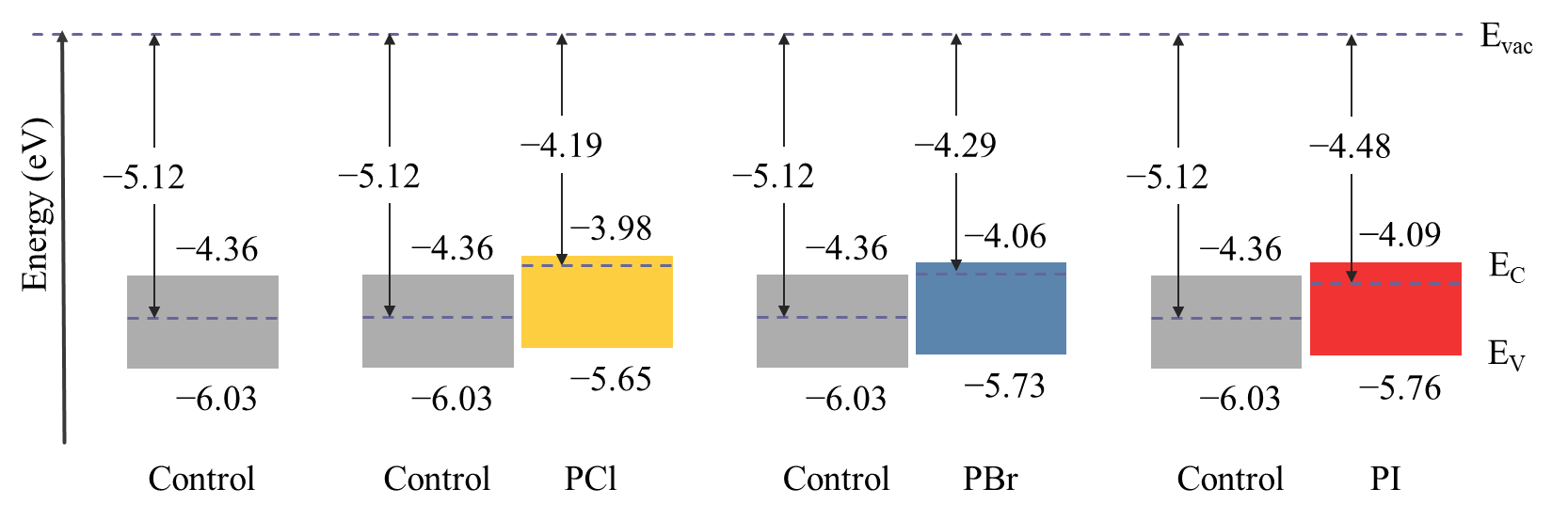
**

**Fig. S32** Energy level diagram of upper surfaces for control, PCl, PBr, and PI perovskite films, reference to the vacuum level

**
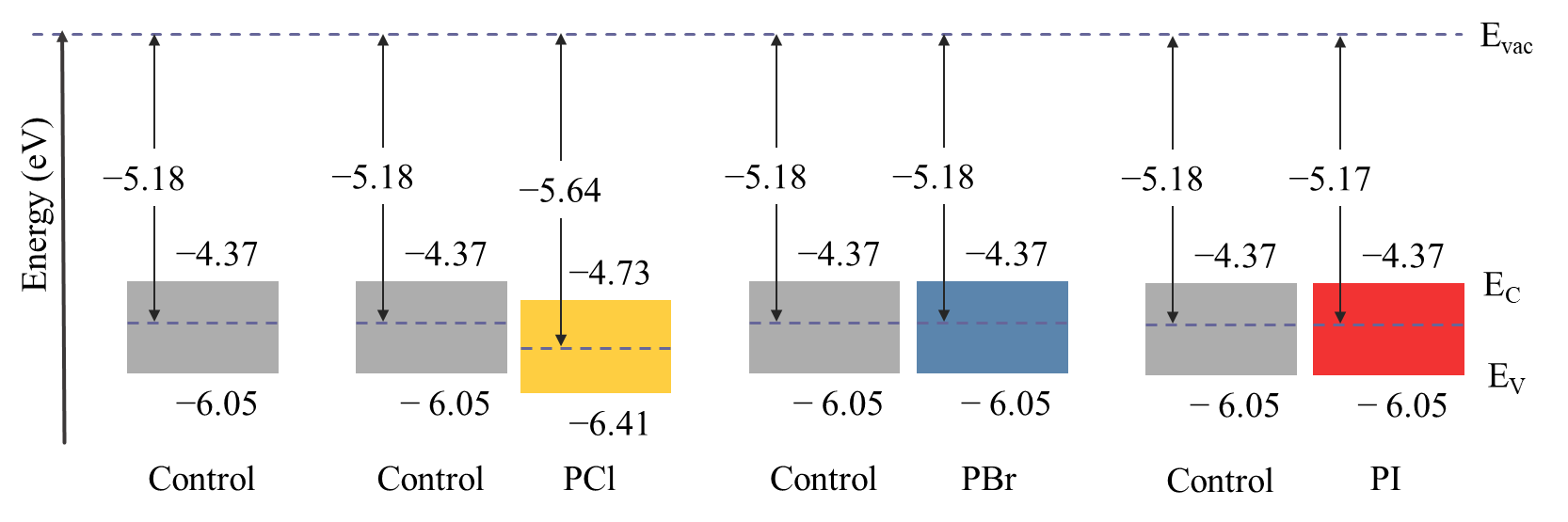
**

**Fig. S33** Energy level diagram of buried interfaces for control, PCl, PBr, and PI perovskite films, reference to the vacuum level

**Fig. S34** Mott–Schottky plots for control, PCl, PBr, and PI devices. The curves on top of the data were obtained by linear fitting the drop region of Mott–Schottky plots, and the built-in electric field was extracted via the intercept of the straight line with the x axis

**
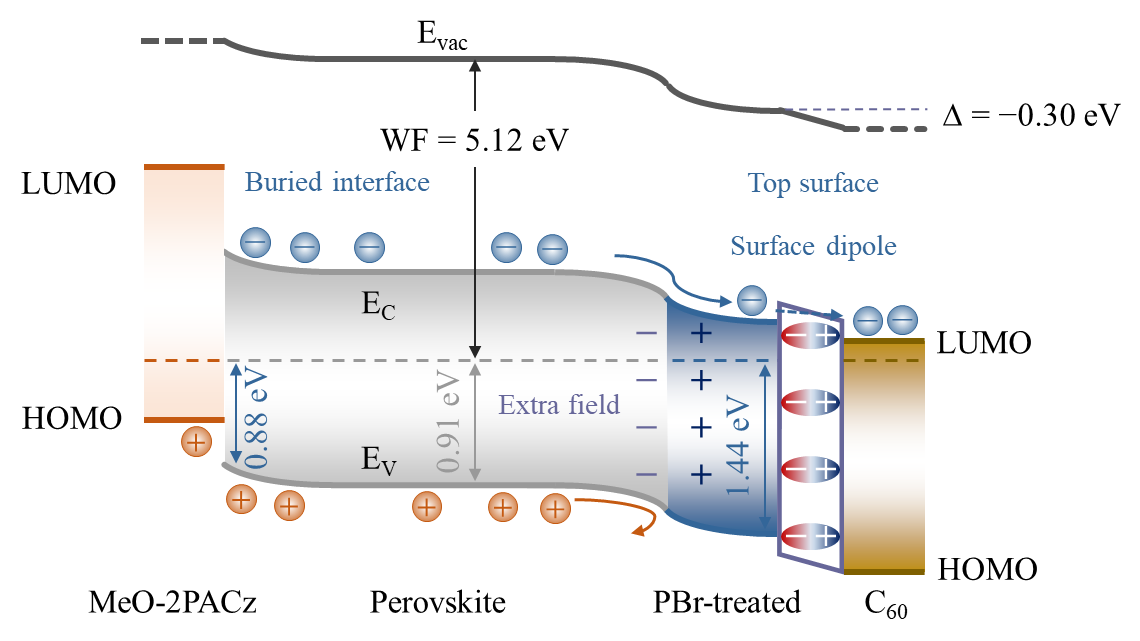
**

**Fig. S35** Energy band diagrams of the PBr film, illustrating the negative surface dipole arrangement, band bending and built-in electric field distribution. E_V_, E_C_, WF, Evac, LUMO and HOMO represent the valence band maximum, conduction band minimum, work function, vacuum energy level, lowest unoccupied molecular orbital and highest occupied molecular orbital, respectively.

**
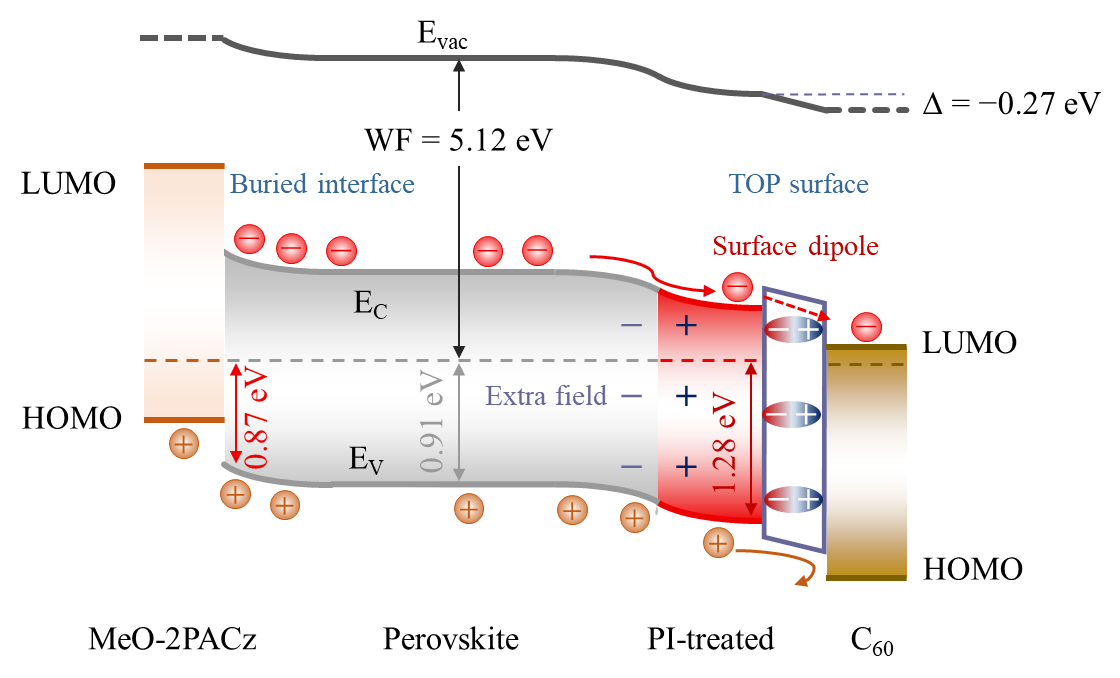
**

**Fig. S36** Energy band diagrams of the PI film, illustrating the negative surface dipole arrangement, band bending and built-in electric field distribution. E_V_, E_C_, WF, Evac, LUMO and HOMO represent the valence band maximum, conduction band minimum, work function, vacuum energy level , lowest unoccupied molecular orbital and highest occupied molecular orbital, respectively.

**
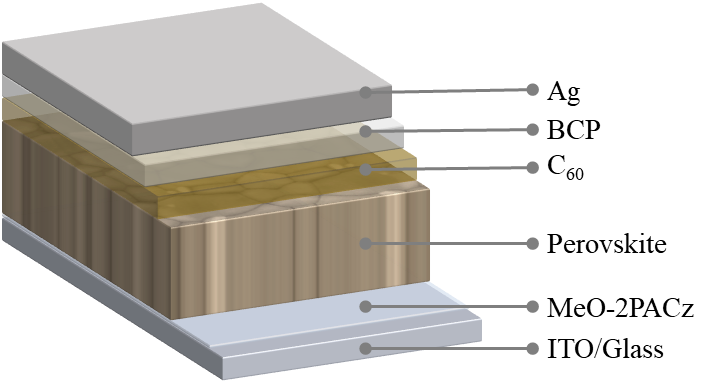
**

**Fig. S37** Device architecture of the perovskite single-junction PSCs

**Fig. S38** *J–V* curves of champion devices treated with 0.14, 0.20, 0.41 and 0.68 mg mL^−1^ of PBr

**Fig. S39** J–V curves of champion devices treated with 0.17, 0.26, 0.52 and 0.87 mg mL^−1^ of PI

**Fig. S40** Statistical distributions of PV parameters for control, PCl, PBr and PI PSCs. The data were based on the *J–V* results from 25 cells

**Fig. S41** Steady-state output of PCEs for the champion control, PCl-treated, PBr-treated and PI-treated PSCs measured at maximum power points and illuminated under 1 sun AM 1.5G

**Fig. S42** EQE characteristic spectra and the corresponding integrated *J*_SC_ profiles of the champion control, PCl-treated, PBr-treated and PI-treated PSCs. The extracted integrated *J*_SCs_ were 20.62, 20.79, 20.68, and 20.49 mA cm^−2^, respectively.


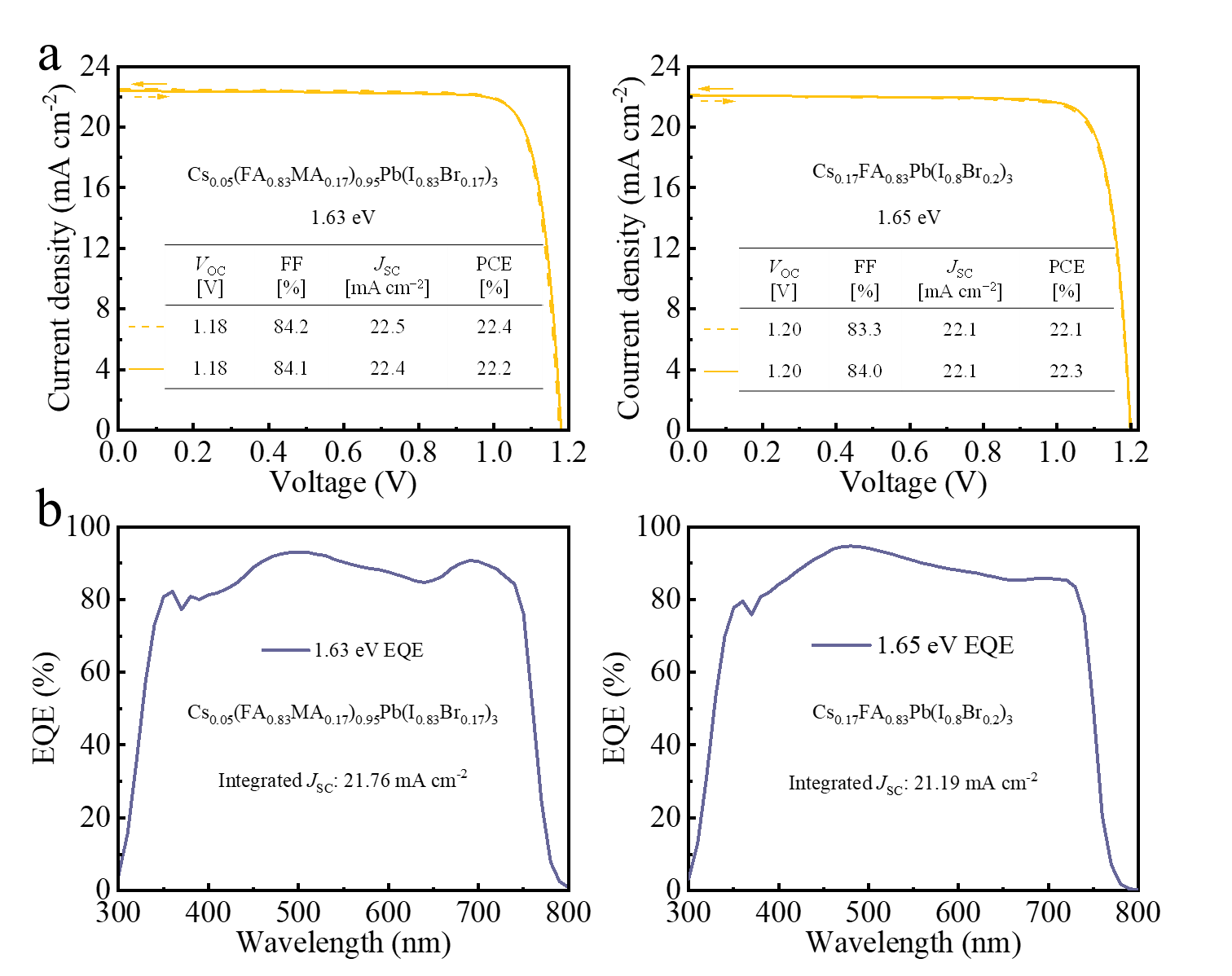


**Fig. S43** a) *J–V* curves of the champion 1.63 eV (left) and 1.65 eV (right) PSCs post-treated with PCl. b) EQE spectra of 1.63 eV (left) and 1.65 eV (right) PSCs

**Fig. S44** *J-V* curves and corresponding photovoltaic parameters of the PCl-treated PSCs with different hole transport layer substrates

**
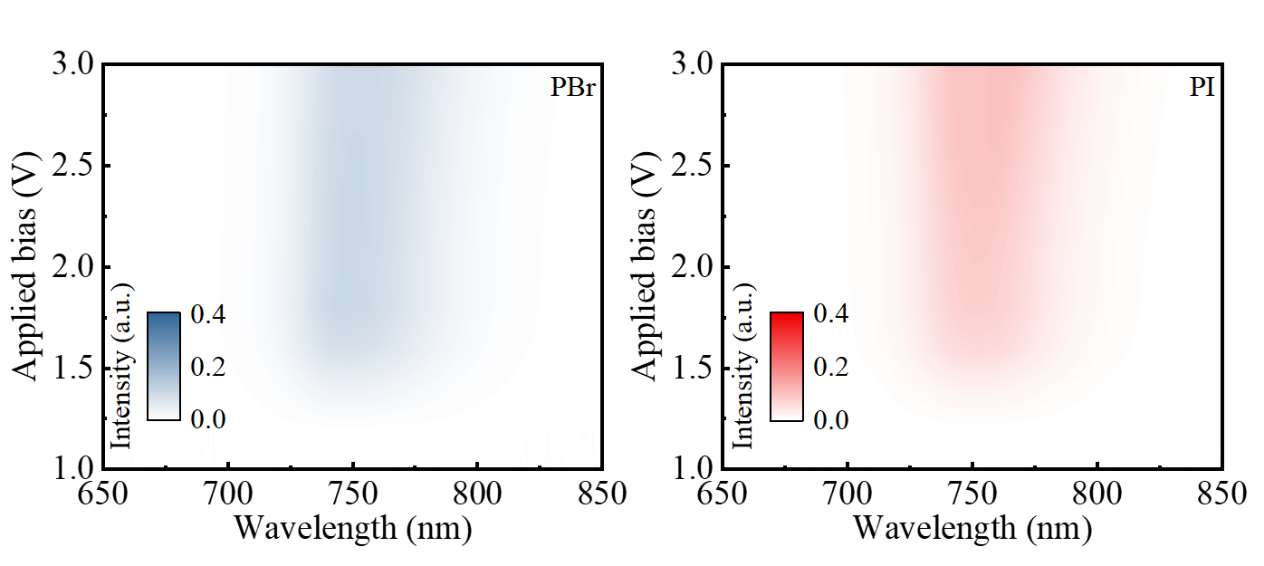
**

**Fig. S45** EL spectra of the PBr-treated and PI-treated PSCs under different applied voltage biases

**Fig. S46** XRD patterns of control, PCl, PBr and PI perovskite films in air ambient (25–35 ℃, 40–60%RH) for various times

**Fig. S47** XRD patterns of control, PCl, PBr and PI perovskite films heated at 85 ℃ in N_2_ (10%RH)

**Fig. S48** The humidity stability of the unencapsulated control, PCl, PBr, and PI PSCs in ambient air (30 ± 5 ℃, 40–60%RH)

**Fig. S49** The thermal stability of the unencapsulated control, PCl, PBr, and PI PSCs under constant heating at 85 °C in N_2_ (10%RH)

**Fig. S50** Continuous MPP tracking of the unencapsulated control, PCl, PBr, and PI PSCs under 1-sun illumination with a white light-emitting diode (100 mW cm^−2^) in ambient air (30 ± 5 °C, 40–60% RH)

**
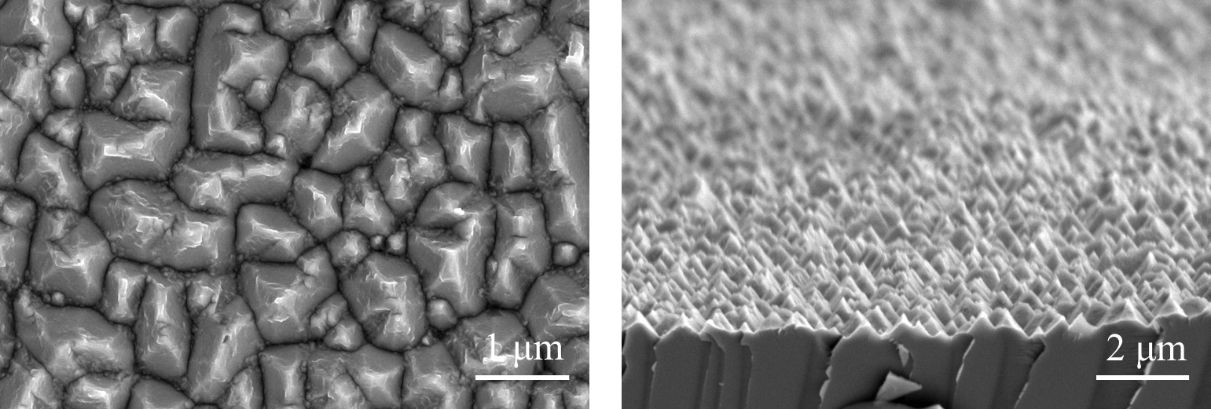
**

**Fig. S51** **a**) Top-view and **b**) cross-section (front side) SEM images of the SHJ bottom cell

**
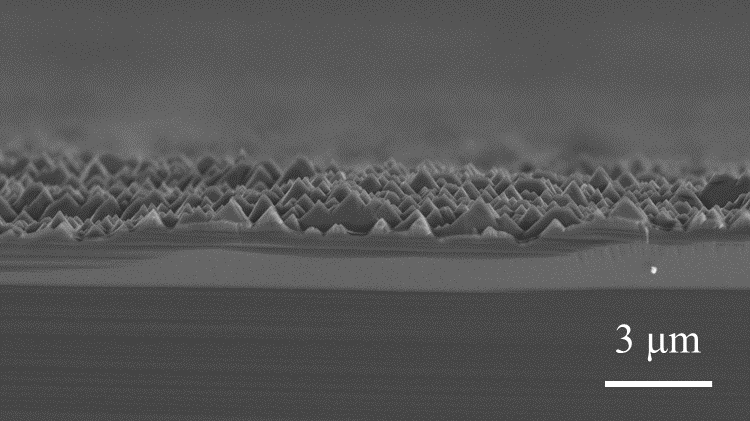
**

**Fig. S52** Cross-section (rear side) SEM image of the SHJ bottom cell

**
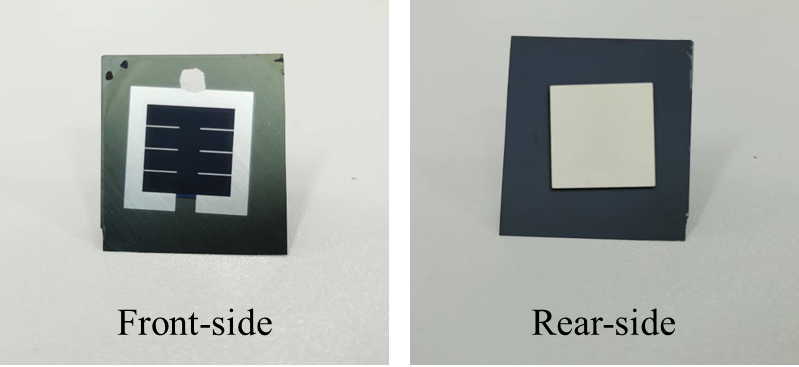
**

**Fig. S53** Photographs of the large-area (1.04 cm^2^) monolithic PVSK/Si TSCs

**Fig. S54** Steady-state output of PCE for the champion large-area (1.04 cm^2^) PCl-based PVSK/Si TSCs

**Fig. S55** Statistical distributions of PV parameters for control and PCL PVSK/Si TSCs. The data were based on the *J–V* results from 25 tandems

**
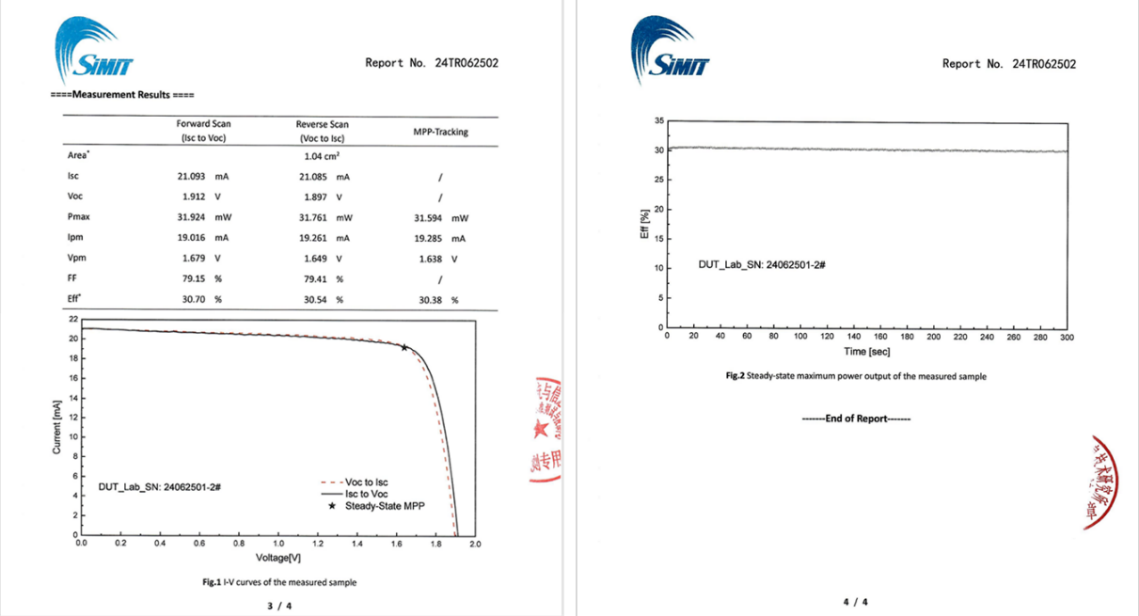
**

**Fig. S56** The certified result of the PCl-based monolithic PVSK/Si TSCs (without encapsulation, 1.04 cm^2^) measured at SIMIT. The device exhibited certified PCEs of 30.54% (*V*_OC_ = 1.897 V, *J*_SC_ = 20.27 mA cm^−2^, FF = 79.41%) under reverse scan and of 30.70% (*V*_OC_ = 1.912 V, *J*_SC_ = 20.28 mA cm^−2^, FF = 79.15%) under forward scan. After the 5 min steady-state MPP tracking, the stabilized PCE of 30.4% (under a V_MPP_ of 1.638 V) was recorded

**Fig. S57** *J‒V* curves of one of the best unencapsulated PCl-based PVSK/Si TSCs after 2304 hours of storage in N_2_


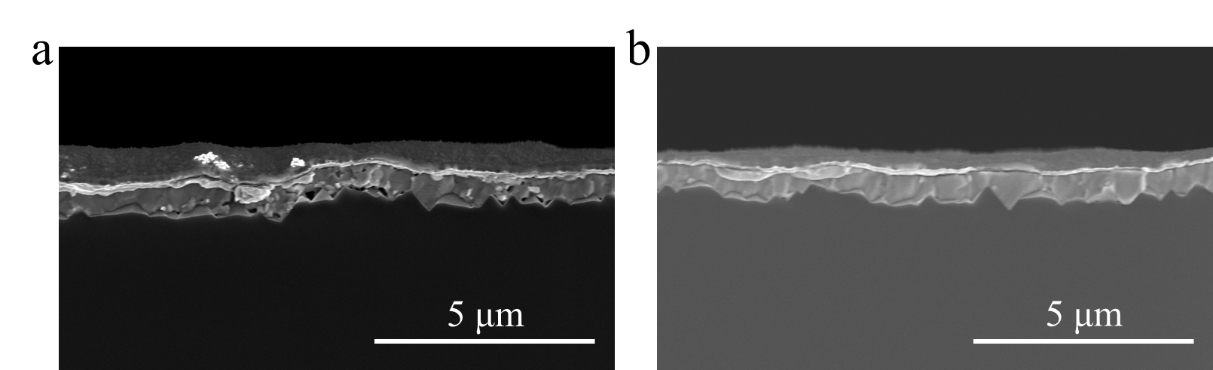


**Fig. S58** Cross-section SEM images of PCl-based PVSK/Si TSCs. **a**) after 755 hours of MPP tracking in ambient air; **b**) after 2304 hours of storage in N_2_

**Table S1** *J‒V* parameters of perovskite single-junction solar cells treated with 0 (control), 0.10, 0.15, 0.30 and 0.50 mg mL^−1^ of PCl

| Concentration  [mg mL^−1^] | *V*_OC_  [V] | FF  [%] | *J*_SC_  [mA cm^−2^] | PCE  [%] |
| --- | --- | --- | --- | --- |
| Control | 1.141 | 80.2 | 20.7 | 19.0 |
| 0.10 | 1.226 | 83.2 | 20.9 | 21.3 |
| 0.15 | 1.254 | 85.6 | 20.8 | 22.3 |
| 0.30 | 1.250 | 82.4 | 20.9 | 21.5 |
| 0.50 | 1.256 | 79.3 | 20.9 | 20.8 |

**Table S2** Slopes of the fitted lines in Fig. 2g and the corresponding residual stresses

| Sample | Slope | Stress (MPa) |
| --- | --- | --- |
| Control | −0.03300 | 13.83 |
| PCl | 0.00131 | −0.55 |
| PBr | −0.00102 | 0.43 |
| PI | −0.02675 | 11.18 |

**Table S3** PLQY and the correspondingly derived QFLS values for different stacks

| Stacks | PLQY (%) | QFLS (eV) |
| --- | --- | --- |
| Neat perovskite | 0.01211 | 1.251 |
| MeO-2PACz/perovskite | 0.0029 | 1.214 |
| MeO-2PACz/perovskite/PCl | 0.01587 | 1.258 |
| MeO-2PACz/perovskite/PBr | 0.00899 | 1.243 |
| MeO-2PACz/perovskite/PI | 0.00679 | 1.236 |
| MeO-2PACz/perovskite/C_60_ | 0.00059 | 1.173 |
| MeO-2PACz/perovskite/PCl/C_60_ | 0.01527 | 1.257 |
| MeO-2PACz/perovskite/PBr/C_60_ | 0.00823 | 1.241 |
| MeO-2PACz/perovskite/PI/C_60_ | 0.00538 | 1.230 |

**Table S4** The TRPL fitted parameters of the glass/ITO/MeO-2PACz/perovskite/PX (X=Cl, Br and I)/C_60_ stack by bi-exponential fitting. The incident excitation light was irradiated from C_60_ side

| Devices | A_1_ | τ_1_  [ns] | A_2_ | τ_2_  [ns] | τ_avg_  [ns] |
| --- | --- | --- | --- | --- | --- |
| Control | 1.99 | 1.19 | 0.24 | 11.09 | 2.25 |
| PCl | 0.56 | 10.01 | 0.51 | 42.01 | 25.26 |
| PBr | 0.50 | 7.19 | 0.60 | 16.93 | 12.50 |
| PI | 0.80 | 4.40 | 0.35 | 14.76 | 7.55 |

**Table S5** The TRPL fitted parameters of the glass/ITO/MeO-2PACz/perovskite/PX (X=Cl, Br and I)/C_60_ stack by bi-exponential fitting. The incident excitation light was irradiated from glass/ITO side

| Devices | A_1_ | τ_1_  [ns] | A_2_ | τ_2_  [ns] | τ_avg_  [ns] |
| --- | --- | --- | --- | --- | --- |
| Control | 0.5 | 67.63 | 0.39 | 10.14 | 42.44 |
| PCl | 0.12 | 16.89 | 0.5 | 116.43 | 97.16 |
| PBr | 0.27 | 24.30 | 0.32 | 107.26 | 69.30 |
| PI | 0.29 | 20.85 | 0.21 | 108.65 | 57.73 |

**Table S6** *J‒V* parameters of perovskite single-junction solar cells treated with 0.14, 0.20, 0.41 and 0.68 mg mL^−1^ of PBr

| Concentration  [mg mL^−1^] | *V*_OC_  [V] | FF  [%] | *J*_SC_  [mA cm^−2^] | PCE  [%] |
| --- | --- | --- | --- | --- |
| 0.14 | 1.223 | 83.0 | 20.4 | 20.7 |
| 0.20 | 1.238 | 83.8 | 20.7 | 21.5 |
| 0.41 | 1.242 | 80.4 | 20.6 | 20.6 |
| 0.68 | 1.248 | 78.2 | 20.6 | 20.1 |

**Table S7** *J‒V* parameters of perovskite single-junction solar cells treated with 0.17, 0.26, 0.52 and 0.87 mg mL^−1^ of PI

| Concentration  [mg mL^−1^] | *V*_OC_  [V] | FF  [%] | *J*_SC_  [mA cm^−2^] | PCE  [%] |
| --- | --- | --- | --- | --- |
| 0.17 | 1.218 | 82.4 | 20.3 | 20.5 |
| 0.26 | 1.224 | 82.7 | 20.6 | 20.8 |
| 0.52 | 1.225 | 82.0 | 20.5 | 20.6 |
| 0.87 | 1.238 | 79.4 | 20.7 | 20.3 |

**Table S8** Photovoltaic parameters derived from the *J−V* curves of the champion control, PCl, PBr and PI single-junction PSCs and the statistical results of *V*_OC_, *J*_SC_, FF, and PCE.

| Device | Scans | *V*_OC_  [V] | *J*_SC_  [mA cm^−2^] | FF  [%] | PCE  [%] | SPO  [%] |
| --- | --- | --- | --- | --- | --- | --- |
| Control | Forward | 1.138 | 20.7 | 80.1 | 18.9 | 18.8 |
|  | Reverse | 1.141 | 20.7 | 80.2 | 19.0 |  |
|  | Average | 1.137±0.011 | 20.6±0.13 | 79.8±0.79 | 18.6±0.26 |  |
| PCl | Forward | 1.251 | 20.8 | 85.5 | 22.2 | 21.8 |
|  | Reverse | 1.254 | 20.8 | 85.6 | 22.3 |  |
|  | Average | 1.249±0.004 | 20.7±0.21 | 84.4±0.41 | 21.6±0.30 |  |
| PBr | Forward | 1.233 | 20.7 | 82.3 | 21.0 | 20.9 |
|  | Reverse | 1.238 | 20.7 | 83.8 | 21.5 |  |
|  | Average | 1.233±0.003 | 20.6±0.17 | 83.7±0.30 | 21.0±0.28 |  |
| PI | Forward | 1.224 | 20.5 | 82.3 | 20.6 | 20.4 |
|  | Reverse | 1.224 | 20.6 | 82.7 | 20.8 |  |
|  | Average | 1.219±0.003 | 20.6±0.19 | 82.7±0.50 | 20.5±0.22 |  |

**Table S9** Summary of reported state-of-the-art 1.68 eV PSCs and comparisons of their *V*_OC_ × FF relative to the S-Q limit. N* = (*V*_OC_ × FF)/(*V*_OC,SQ_ × FF_SQ_).

| *V*_OC_  [V] | FF  [%] | *J*_SC_  [mA cm^−2^] | PCE  [%] | N*  [%] | Refs. |
| --- | --- | --- | --- | --- | --- |
| 1.272 | 82.01 | 21.09 | 22.00 | 82.0 | [S14] |
| 1.262 | 82.7 | 20.9 | 21.8 | 82.1 | [S15] |
| 1.239 | 82.5 | 21.16 | 21.63 | 80.4 | [S16] |
| 1.248 | 83.9 | 21.11 | 22.10 | 82.3 | [S17] |
| 1.26 | 82.6 | 20.5 | 21.3 | 81.8 | [S18] |
| 1.24 | 83.07 | 21.18 | 21.82 | 81.0 | [S19] |
| 1.25 | 84.5 | 21.5 | 22.7 | 83.0 | [S20] |
| 1.27 | 78.86 | 20.51 | 20.58 | 78.7 | [S21] |
| 1.242 | 82.97 | 20.41 | 21.02 | 81.0 | [S22] |
| 1.24 | 83.44 | 20.81 | 21.53 | 81.3 | [S23] |
| 1.22 | 82.35 | 21.84 | 22.02 | 79.0 | [S24] |
| 1.25 | 83.9 | 21.02 | 22.06 | 82.5 | [S25] |
| 1.21 | 83.7 | 20.6 | 20.9 | 79.6 | [S26] |
| 1.202 | 82.0 | 20.73 | 20.43 | 77.5 | [S27] |
| 1.216 | 82.87 | 22.18 | 22.35 | 79.2 | [S28] |
| 1.21 | 82.1 | 20.3 | 20.18 | 78.1 | [S29] |
| 1.22 | 77.22 | 20.38 | 19.24 | 74.1 | [S30] |
| 1.19 | 81.8 | 20.94 | 20.31 | 76.5 | [S31] |
| 1.19 | 83.1 | 21.84 | 21.6 | 77.7 | [S32] |
| 1.236 | 83.7 | 20.6 | 20.9 | 81.3 | [S33] |
| 1.2 | 81.47 | 20.39 | 19.88 | 76.9 | [S34] |
| 1.207 | 80.8 | 20.8 | 20 | 76.7 | [S35] |
| 1.19 | 80.07 | 20.54 | 19.6 | 74.9 | [S36] |
| 1.2 | 84 | 20.79 | 20.96 | 79.2 | [S27] |
| 1.215 | 84.28 | 20.59 | 21.1 | 80.5 | [S26] |
| 1.202 | 82.39 | 21.33 | 21.13 | 77.9 | [S37] |
| 1.204 | 81.73 | 20.72 | 20.39 | 77.4 | [S38] |
| 1.218 | 80.6 | 80.6 | 20.2 | 77.2 | [S39] |
| 1.23 | 78.71 | 20.45 | 19.79 | 76.1 | [S40] |
| 1.21 | 76 | 17.97 | 16.53 | 72.3 | [S41] |
| 1.185 | 80.5 | 21.28 | 20.3 | 77.8 | [S42] |
| 1.20 | 81.02 | 20.94 | 20.38 | 76.4 | [S43] |
| 1.196 | 81.5 | 21.65 | 21.1 | 76.6 | [S44] |
| 1.16 | 82.9 | 20.09 | 19.28 | 75.6 | [S45] |
| 1.22 | 81.46 | 20.98 | 20.77 | 78.1 | [S46] |
| 1.28 | 81.71 | 19.87 | 20.78 | 82.2 | [S47] |
| 1.22 | 76.9 | 20.6 | 19.3 | 73.8 | [S48] |
| 1.21 | 83.7 | 20.6 | 20.9 | 79.6 | [S49] |
| 1.185 | 83.53 | 21.66 | 21.44 | 77.8 | [S50] |
| 1.26 | 84.0 | 19.5 | 20.6 | 83.2 | [S51] |
| 1.293 | 79.43 | 20.84 | 21.41 | 80.7 | [S52] |
| 1.236 | 86.67 | 21.12 | 22.63 | 84.2 | [S53] |
| 1.234 | 81.22 | 20.90 | 20.95 | 78.8 | [S54] |
| 1.25 | 83.1 | 22.31 | 23.05 | 81.7 | [S55] |
| 1.175 | 81.81 | 22.45 | 21.58 | 75.6 | [S56] |
| 1.18 | 81.76 | 22.56 | 21.76 | 75.9 | [S7] |
| 1.17 | 79.8 | 21.2 | 19.8 | 73.4 | [S57] |
| 1.254 | 85.6 | 20.8 | 22.3 | 84.4 | **This work** |

**Table S10** Photovoltaic parameters of reported state-of-the-art PVSK/Si TSCs with efficiencies exceeding 30%, alongside their associated passivation strategies.

| *V*_OC_  [V] | FF  [%] | *J*_SC_  [mA cm^−2^] | PCE  [%] | Target | Strategy | Surface structure of silicon | Refs. |
| --- | --- | --- | --- | --- | --- | --- | --- |
| 1.91 | 79.8 | 20.47 | 31.25 | Top surface | FBPAc additive | Micron-scale pyramid | [S58] |
| 1.98 | 81.18 | 20.24 | 32.5 | Top surface | PI post-treatment | Planar | [S21] |
| 1.949 | 80.5 | 20.9 | 32.8 | Recombination layer | Amorphous IZO interconnect | Submicron-pyramid | [S59] |
| 1.91 | 79.29 | 20.47 | 31.01 | Top surface | CF_3_-TEA dynamic spray coating | Micron-scale pyramid | [S60] |
| 1.903 | 78.7 | 20.1 | 30.0 | Top surface | Urea additive | Micron-scale pyramid | [S61] |
| 1.97 | 77.71 | 20.08 | 30.8 | Buffer layer | Sn-doped In_2_O_3_ buffer | Planar | [S62] |
| 1.81 | 82.91 | 20.01 | 30.1 | Top surface | F-PMAI post-treatment | Micron-scale pyramid | [S63] |
| 1.87 | 83.33 | 20.65 | 32.13 | Buried interface | CsPbCl_3_ seed layer | Micron-scale pyramid | [S64] |
| 1.899 | 80.59 | 20.54 | 31.42 | Top surface | Binary 2D perovskite post-treatment | Submicron-pyramid | [S54] |
| 1.929 | 81.54 | 19.58 | 30.80 | Buried interface | Potassium sorbate additive | Planar | [S14] |
| 1.953 | 79.9 | 19.8 | 30.9 | Buried interface | SiO_2_-NPs post-treatment + FBPAc additive | Planar | [S65] |
| 1.985 | 81.6 | 21.02 | 34.0 | Bulk | THTZ-H^+^ additive | Submicron-pyramid | [S66] |
| 1.96 | 81.0 | 20.4 | 32.5 | Bulk | OAmI additive | Submicron-pyramid | [S67] |
| 1.96 | 78.64 | 20.01 | 30.78 | Top surface | CcPF_6_ post-treatment | Submicron-pyramid | [S47] |
| 1.80 | 85.4 | 20.0 | 30.7 | Texture design | Pyramid micro/nano-reconstruction | Micro/nano-pyramid | [S68] |
| 1.9 | 79.8 | 20.12 | 30.5 | Hole transport layer | ALD Cu-NiO_X_ | Submicron-pyramid | [S69] |
| 1.918 | 80.9 | 20.3 | 31.5 | Dual-interface | PCl post-treatment | Submicron-pyramid | This work |

**Table S11** Summary of MPP stability for monolithic perovskite/silicon tandems reported in press releases or the literature

| Structure | Environment | Encapsulation | Light condition | PCE retention | Refs. |
| --- | --- | --- | --- | --- | --- |
| p-i-n | In ambient air; 20–30% RH; 25 ℃ | no | Two-lamp (halogen and xenon) (100 mW cm^−2^) | T_92_=61 h | [S70] |
| p-i-n | In ambient air;  25–35%RH; 30 ℃ | no | 1-sun illumination | T_92_=100 h | [S71] |
| p-i-n | In ambient air;  30–40%RH; 25 ℃ | no | LED with wavelengths of 470 and 940 nm | T_95.5_=300 h | [S72] |
| p-i-n | In N_2_ atmosphere; 25±1 ℃ | no | 1-sun illumination | T_90_=100 h | [S73] |
| p-i-n | In ambient air; 55 ℃ | no | 1-sun illumination | T_100_=2 h | [S74] |
| p-i-n | In ambient air; 20%RH; 25 ℃ | no | 1-sun illumination | T_98_=300 h | [S75] |
| p-i-n | In ambient air | no | 1-sun illumination | T_99_=4.3 h | [S76] |
| p-i-n | In N_2_ atmosphere; 25±1 ℃ | no | 1-sun illumination | T_118.2_=100 h | [S77] |
| p-i-n | In N_2_ atmosphere; 25 ℃ | no | White LED light source  (940 nm LED light for extra compensation) | T_98_=200 h | [S29] |
| p-i-n | In ambient air; 40–70%RH; 20–35 ℃ | no | Xenon lamp  (100 mW cm^−2^) | T_93.6_=450 h | [S78] |
| p-i-n | In ambient air; 40–70%RH; 20–40 ℃ | no | Xenon lamp  (100 mW cm^−2^) | T_90_=64 h | [S79] |
| p-i-n | In ambient air; 25–75%RH, most often ≈60%RH; 20–35 ℃ | no | Xenon lamp  (100 mW cm^−2^) | T_86.6_=306 h | [S80] |
| p-i-n | In N_2_ atmosphere; 25 ℃ | no | 1-sun illuminations | T_80_=1200 h | [S81] |
| p-i-n | In ambient air; 30–40%RH; 25℃ | no | 1-sun illuminations | T_100_=700 h | [S63] |
| p-i-n | In ambient air; 25%RH; 25℃ | no | 1-sun illuminations | T_96_=527 h | [S54] |
| p-i-n | In N_2_ atmosphere; 25 ℃ | no | 1-sun LED illumination | T_90_=1200 h | [S82] |
| p-i-n | In ambient air; 30–50%RH; 25℃ | no | 1-sun illumination (without a UV filter) | T_85_=84 h | [S65] |
| p-i-n | In ambient air; 50%RH; 75℃ | no | 1-sun illumination | T_82_=5 h | [S66] |
| p-i-n | In ambient air; 40%RH; 25℃ | no | 1-sun illumination | T_90.6_=100 h | [S83] |
| p-i-n | In N_2_ atmosphere; 25 ℃ | no | 1-sun illumination | T_95_=280 h | [S84] |
| n-i-p | In N_2_ atmosphere; 25 ℃ | no | 1-sun LED illumination | T_80_=700 h | [S85] |
| p-i-n | In ambient air; 40–60%RH; 25–35 ℃ | no | 1-sun xenon-lamp illumination (without a UV filter) | T_80_=755 h | This work |

**Supplementary References**

1. S. Liu, J. Li, W. Xiao, R. Chen, Z. Sun et al., Buried interface molecular hybrid for inverted perovskite solar cells. Nature **632**(8025), 536-542 (2024). <https://doi.org/10.1038/s41586-024-07723-3>
2. W. Li, W. Zhang, S. Van Reenen, R.J. Sutton, J. Fan et al., Enhanced UV-light stability of planar heterojunction perovskite solar cells with caesium bromide interface modification. Energy Environ Sci. **9**(2), 490-498 (2016). <https://doi.org/10.1039/C5EE03522H>
3. F. Yang, P. Tockhorn, A. Musiienko, F. Lang, D. Menzel et al., Minimizing Interfacial Recombination in 1.8 Ev Triple-Halide Perovskites for 27.2% Efficient All-Perovskite Tandems. Adv Mater. **36**(6), 2307743 (2023). <https://doi.org/10.1002/adma.202307743>
4. S. Hu, K. Otsuka, R. Murdey, T. Nakamura, M.A. Truong et al., Optimized carrier extraction at interfaces for 23.6% efficient tin–lead perovskite solar cells. Energy Environ Sci. **15**(5), 2096-2107 (2022). <https://doi.org/10.1039/D2EE00288D>
5. L. Shen, P. Song, L. Zheng, L. Wang, X. Zhang et al., Ion diffusion management enables all-interface defect passivation of perovskite solar cells. Adv Mater. **35**(39), 2301624 (2023). <https://doi.org/10.1002/adma.202301624>
6. W. Zhao, J. Xu, K. He, Y. Cai, Y. Han et al., A special additive enables all cations and anions passivation for stable perovskite solar cells with efficiency over 23%. Nano-Micro Letters. **13**(1), 169 (2021). <https://doi.org/10.1007/s40820-021-00688-2>
7. Y. Zheng, X. Wu, J. Liang, Z. Zhang, J. Jiang et al., Downward homogenized crystallization for inverted wide-bandgap mixed-halide perovskite solar cells with 21% efficiency and suppressed photo-induced halide segregation. Adv Funct Mater. **32**(29), 2200431 (2022). <https://doi.org/10.1002/adfm.202200431>
8. H. Wang, F. Ye, J. Liang, Y. Liu, X. Hu et al., Pre-annealing treatment for high-efficiency perovskite solar cells via sequential deposition. Joule. **6**(12), 2869-2884 (2022). <https://doi.org/10.1016/j.joule.2022.10.001>
9. S. Wang, M.-H. Li, Y. Zhang, Y. Jiang, L. Xu et al., Surface N-type band bending for stable inverted CsPbI3 perovskite solar cells with over 20% efficiency. Energy Environ Sci. **16**(6), 2572-2578 (2023). <https://doi.org/10.1039/D3EE00423F>
10. Y. Zhou, Z. Wang, J. Jin, X. Zhang, J. Zou et al., Manipulation of the buried interface for robust formamidinium-based Sn-Pb perovskite solar cells with NiO(x) hole-transport layers. Angew Chem Int Ed Engl. **62**(15), e202300759 (2023). <https://doi.org/10.1002/anie.202300759>
11. Y. Li, P.J. Lohr, A. Segapeli, J. Baltram, D. Werner et al., Influence of halides on the interactions of ammonium acids with metal halide perovskites. ACS Appl Mater Interfaces. **15**(20), 24387-24398 (2023). <https://doi.org/10.1021/acsami.3c01432>
12. X. Zheng, B. Chen, J. Dai, Y. Fang, Y. Bai et al., Defect passivation in hybrid perovskite solar cells using quaternary ammonium halide anions and cations. Nat Energy. **2**(7), 17102 (2017). <https://doi.org/10.1038/nenergy.2017.102>
13. N. Li, S. Tao, Y. Chen, X. Niu, C.K. Onwudinanti et al., Cation and anion immobilization through chemical bonding enhancement with fluorides for stable halide perovskite solar cells. Nat Energy. **4**(5), 408-415 (2019). <https://doi.org/10.1038/s41560-019-0382-6>
14. L. Qiao, T. Ye, T. Wang, W. Kong, R. Sun et al., Freezing halide segregation under intense light for photostable perovskite/silicon tandem solar cells. Adv Energy Mater. **14**(7), 2302983 (2024). <https://doi.org/10.1002/aenm.202302983>
15. Z. Liu, H. Li, Z. Chu, R. Xia, J. Wen et al., Reducing perovskite/c(60) interface losses via sequential interface engineering for efficient perovskite/silicon tandem solar cell. Adv Mater. **36**(8), e2308370 (2024). <https://doi.org/10.1002/adma.202308370>
16. N. Yan, Y. Gao, J. Yang, Z. Fang, J. Feng et al., Wide-bandgap perovskite solar cell using a fluoride-assisted surface gradient passivation strategy. Angew Chem Int Ed. **62**(11), e202216668 (2023). <https://doi.org/10.1002/anie.202216668>
17. H. Zhang, S. Zhang, X. Ji, J. He, H. Guo et al., Formamidinium lead iodide-based inverted perovskite solar cells with efficiency over 25% enabled by an amphiphilic molecular hole-transporter. Angew Chem Int Ed. **63**(16), e202401260 (2024). <https://doi.org/10.1002/anie.202401260>
18. G. Wang, J. Zheng, W. Duan, J. Yang, M.A. Mahmud et al., Molecular engineering of hole-selective layer for high band gap perovskites for highly efficient and stable perovskite-silicon tandem solar cells. Joule. **7**(11), 2583-2594 (2023). <https://doi.org/10.1016/j.joule.2023.09.007>
19. Y. Yang, Q. Chang, Y. Yang, Y. Jiang, Z. Dai et al., Multifunctional molecule interface modification for high-performance inverted wide-bandgap perovskite cells and modules. J Mater Chem A. **11**(31), 16871-16877 (2023). <https://doi.org/10.1039/D3TA02209A>
20. Z. Li, X. Sun, X. Zheng, B. Li, D. Gao et al., Stabilized hole-selective layer for high-performance inverted p-i-n perovskite solar cells. Science. **382**(6668), 284-289 (2023). <https://doi.org/10.1126/science.ade9637>
21. S. Mariotti, E. Köhnen, F. Scheler, K. Sveinbjörnsson, L. Zimmermann et al., Interface engineering for high-performance, triple-halide perovskite–silicon tandem solar cells. Science. **381**(6653), 63-69 (2023). <https://doi.org/10.1126/science.adf5872>
22. S. Zhang, F. Ye, X. Wang, R. Chen, H. Zhang et al., Minimizing buried interfacial defects for efficient inverted perovskite solar cells. Science. **380**(6643), 404-409 (2023). <https://doi.org/10.1126/science.adg3755>
23. Z. Xiong, L. Wu, X. Zhou, S. Yang, Z. Liu et al., Constructing tin oxides interfacial layer with gradient compositions for efficient perovskite/silicon tandem solar cells with efficiency exceeding 28. Small. **20**(15), e2308024 (2023). <https://doi.org/10.1002/smll.202308024>
24. Y. Gan, X. Hao, W. Li, J. Zhang, L. Wu, Additive combining passivator for inverted wide‐bandgap perovskite solar cells with 22% efficiency and reduced voltage loss. Sol RRL. **7**(24), 2300519 (2023). <https://doi.org/10.1002/solr.202300519>
25. X. Han, J. Wang, L. Jin, P. Wang, B. Shi et al., Zwitterion reduces open-circuit voltage loss in wide-bandgap perovskite solar cells with 22% efficiency and its application in tandem devices. Sol RRL. **n/a**(n/a), (2023). https://doi.org/10.1002/solr.202300648
26. C. Li, Z. Zhang, H. Zhang, W. Yan, Y. Li et al., Fully aromatic self-assembled hole-selective layer toward efficient inverted wide-bandgap perovskite solar cells with ultraviolet resistance. Angew Chem Int Ed. **63**(1), e202315281 (2023). <https://doi.org/10.1002/anie.202315281>
27. T. Han, W. Zhu, T. Wang, M. Yang, Y. Zhou et al., MXene-interconnected two-terminal, mechanically-stacked perovskite/silicon tandem solar cell with high efficiency. Adv Funct Mater. **34**(12), 2311679 (2023). <https://doi.org/10.1002/adfm.202311679>
28. L. Yang, Z. Fang, Y. Jin, H. Feng, B. Deng et al., Suppressing halide segregation via pyridine-derivative isomers enables efficient 1.68-ev bandgap perovskite solar cells. Adv Mater. **36**(21), 2311923 (2024). <https://doi.org/10.1002/adma.202311923>
29. S.G. Ji, I.J. Park, H. Chang, J.H. Park, G.P. Hong et al., Stable pure-iodide wide-band-gap perovskites for efficient Si tandem cells via kinetically controlled phase evolution. Joule. **6**(10), 2390-2405 (2022). <https://doi.org/10.1016/j.joule.2022.08.006>
30. B. Chen, P. Wang, R. Li, N. Ren, Y. Chen et al., Composite electron transport layer for efficient N-I-P type monolithic perovskite/silicon tandem solar cells with high open-circuit voltage. J Energy Chem. **63**(461-467 (2021). <https://doi.org/10.1016/j.jechem.2021.07.018>
31. C. Chen, J. Liang, J. Zhang, X. Liu, X. Yin et al., Interfacial engineering of a thiophene-based 2D/3D perovskite heterojunction for efficient and stable inverted wide-bandgap perovskite solar cells. Nano Energy. **90**(106608 (2021). <https://doi.org/10.1016/j.nanoen.2021.106608>
32. X. Zheng, Z. Li, Y. Zhang, M. Chen, T. Liu et al., Co-deposition of hole-selective contact and absorber for improving the processability of perovskite solar cells. Nat Energy. **8**(5), 462-+ (2023). <https://doi.org/10.1038/s41560-023-01227-6>
33. A. Al-Ashouri, M. Marčinskas, E. Kasparavičius, T. Malinauskas, A. Palmstrom et al., Wettability improvement of a carbazole-based hole-selective monolayer for reproducible perovskite solar cells. ACS Energy Lett. **8**(2), 898-900 (2023). <https://doi.org/10.1021/acsenergylett.2c02629>
34. X. Guo, C. Lu, W. Zhang, H. Yuan, H. Yang et al., In situ surface sulfidation of cspbi3 for inverted perovskite solar cells. ACS Energy Lett. **9**(1), 329-335 (2024). <https://doi.org/10.1021/acsenergylett.3c01855>
35. H. Luo, X. Zheng, W. Kong, Z. Liu, H. Li et al., Inorganic framework composition engineering for scalable fabrication of perovskite/silicon tandem solar cells. ACS Energy Lett. **8**(12), 4993-5002 (2023). <https://doi.org/10.1021/acsenergylett.3c02002>
36. Y. Wang, S. Akel, B. Klingebiel, T. Kirchartz, Hole Transporting bilayers for efficient micrometer-thick perovskite solar cells. Adv Energy Mater. **14**(5), 2302614 (2023). <https://doi.org/10.1002/aenm.202302614>
37. T. Nie, J. Yang, Z. Fang, Z. Xu, X. Ren et al., Amino-acid-type alkylamine additive for high-performance wide-bandgap perovskite solar cells. Chem Eng J. **468**, 143341 (2023). https://doi.org/10.1016/j.cej.2023.143341
38. Z. Fang, L. Jia, N. Yan, X. Jiang, X. Ren et al., Proton-transfer-induced in situ defect passivation for highly efficient wide-bandgap inverted perovskite solar cells. InfoMat. **4**(6), e12307 (2022). https://doi.org/10.1002/inf2.12307
39. J. Liu, E. Aydin, J. Yin, M. De Bastiani, F.H. Isikgor et al., 28.2%-efficient, outdoor-stable perovskite/silicon tandem solar cell、27.1（3.8cm2）、28.2（1cm2）. Joule. **5**(12), 3169-3186 (2021). <https://doi.org/10.1016/j.joule.2021.11.003>
40. X. Sun, Z. Shao, Z. Li, D. Liu, C. Gao et al., Highly efficient CsPbI3/Cs1-xDMAxPbI_3_ bulk heterojunction perovskite solar cell. Joule. **6**(4), 850-860 (2022). https://doi.org/10.1016/j.joule.2022.02.004
41. T. He, S. Li, Y. Jiang, C. Qin, M. Cui et al., Reduced-dimensional perovskite photovoltaics with homogeneous energy landscape. Nat Commun. **11**(1), 1672 (2020). <https://doi.org/10.1038/s41467-020-15451-1>
42. W. Chai, L. Li, W. Zhu, D. Chen, L. Zhou et al., Graded heterojunction improves wide-bandgap perovskite for highly efficient 4-terminal perovskite/silicon tandem solar cells. Research. **6**, 0196 (2023). <https://doi.org/10.34133/research.0196>
43. H. Guo, Y. Fang, Y. Lei, J. Wu, M. Li et al., Mitigating ion migration with an ultrathin self-assembled ionic insulating layer affords efficient and stable wide-bandgap inverted perovskite solar cells. Small. **19**(n/a), 2302021 (2023). https://doi.org/10.1002/smll.202302021
44. J.Y. Ye, J. Tong, J. Hu, C. Xiao, H. Lu et al., Enhancing charge transport of 2d perovskite passivation agent for wide-bandgap perovskite solar cells beyond 21%. Sol RRL. **4**(6), 2000082 (2020). https://doi.org/10.1002/solr.202000082
45. H. Guo, C. Liu, H. Hu, S. Zhang, X. Ji et al., Neglected acidity pitfall: boric acid-anchoring hole-selective contact for perovskite solar cells. Natl Sci Rev. **10**(5), nwad057 (2023). <https://doi.org/10.1093/nsr/nwad057>
46. X. Zheng, W. Kong, J. Wen, J. Hong, H. Luo et al., Solvent engineering for scalable fabrication of perovskite/silicon tandem solar cells in air. Nat Commun. **15**(1), 4907 (2024). <https://doi.org/10.1038/s41467-024-49351-5>
47. C. Wang, S. Wang, W. Shi, Z. Su, K. Gao et al., Solvent-assisted surface modification using metallocene-based molecules for high-efficiency perovskite/silicon tandem solar cells. Adv Energy Mater. **14**(31), 2401039 (2024). https://doi.org/10.1002/aenm.202401039
48. J. Zhao, A.S.R. Chesman, J. Yan, L.J. Sutherland, J. Jasieniak et al., Precursor engineering of lead acetate-based precursors for high-open-circuit voltage wide-bandgap perovskite solar cells. ACS Appl Mater Interfaces. **15**(15), 18800-18807 (2023). <https://doi.org/10.1021/acsami.2c22179>
49. S.-W. Kim, S.J. Moon, S.-H. Kim, J.J. Yoo, D. Kim et al., Reducing humidity dependency of ambient-air-processed wide-bandgap inverted perovskite solar cells. ACS Energy Lett. **8**(11), 4777-4781 (2023). <https://doi.org/10.1021/acsenergylett.3c01952>
50. X. Niu, N. Li, Z. Cui, L. Li, F. Pei et al., Anion confinement for homogeneous mixed halide perovskite film growth by electrospray. Adv Mater. **35**(45), e2305822 (2023). <https://doi.org/10.1002/adma.202305822>
51. Y.-H. Lin, V. null, F. Yang, X.-L. Cao, A. Dasgupta et al., Bandgap-universal passivation enables stable perovskite solar cells with low photovoltage loss. Science. **384**(6697), 767-775 (2024). <https://doi.org/10.1126/science.ado2302>
52. X. Ji, S. Zhang, F. Yu, H. Zhang, L. Zhan et al., Efficient wide-bandgap perovskite solar cells with open-circuit voltage deficit below <sc>0.4 V</sc> <italic>via</italic> hole-selective interface engineering. Sci China Chem. **67**(6), 2102-2110 (2024). https://doi.org/10.1007/s11426-023-1966-1
53. C. Li, Y.H. Li, Y. Chen, H.F. Zhang, S.T. Zhang et al., Enhancing efficiency of industrially-compatible monolithic perovskite/silicon tandem solar cells with dually-mixed self-assembled monolayers. Adv Funct Mater. 2407805 (2024). <https://doi.org/10.1002/adfm.202407805>
54. F. Pei, Y. Chen, Q. Wang, L. Li, Y. Ma et al., A binary 2D perovskite passivation for efficient and stable perovskite/silicon tandem solar cells. Nat Commun. **15**(1), 7024 (2024). <https://doi.org/10.1038/s41467-024-51345-2>
55. T. Huang, F. Xu, J. Hu, J. Wu, S. Li et al., Rational heterostructure stacking enables 23% wide-bandgap perovskite solar cells by side-reaction inhibition. Energy Environ Sci. **17**(16), 5984-5992 (2024). <https://doi.org/10.1039/D4EE01547A>
56. Y.-a. Ding, X. Yang, X. Wang, Y. Liu, Y. Yan et al., Multifunctional spacer in 2d/3d wide-bandgap perovskite for monolithic perovskite/silicon tandem solar cells. Sol RRL. **8**(11), 2400189 (2024). https://doi.org/10.1002/solr.202400189
57. D.H. Kim, C.P. Muzzillo, J. Tong, A.F. Palmstrom, B.W. Larson et al., Bimolecular additives improve wide-band-gap perovskites for efficient tandem solar cells with CIGS. Joule. **3**(7), 1734-1745 (2019). https://doi.org/10.1016/j.joule.2019.04.012
58. X.Y. Chin, D. Turkay, J.A. Steele, S. Tabean, S. Eswara et al., Interface passivation for 31.25%-efficient perovskite/silicon tandem solar cells. Science. **381**(6653), 59-63 (2023). <https://doi.org/10.1126/science.adg0091>
59. E. Aydin, E. Ugur, B.K. Yildirim, T.G. Allen, P. Dally et al., Enhanced optoelectronic coupling for perovskite-silicon tandem solar cells. Nature. **623**(7988), 732-738 (2023). <https://doi.org/10.1038/s41586-023-06667-4>
60. J. Chen, S. Yang, L. Jiang, K. Fan, Z. Liu et al., Surface molecular engineering for fully textured perovskite/silicon tandem solar cells. Angew Chem Int Ed. e202407151 (2024). https://doi.org/10.1002/anie.202407151
61. O. Er-raji, M.A.A. Mahmoud, O. Fischer, A.J. Ramadan, D. Bogachuk et al., Tailoring perovskite crystallization and interfacial passivation in efficient, fully textured perovskite silicon tandem solar cells. Joule. (2024). https://doi.org/10.1016/j.joule.2024.06.018
62. Y. Jin, H. Feng, Z. Fang, H. Zhang, L. Yang et al., Efficient and stable monolithic perovskite/silicon tandem solar cells enabled by contact-resistance-tunable indium tin oxide interlayer. Adv Mater. **36**(35), 2404010 (2024). https://doi.org/10.1002/adma.202404010
63. J. Liu, B. Shi, Q. Xu, Y. Li, Y. Li et al., Textured perovskite/silicon tandem solar cells achieving over 30% efficiency promoted by 4-fluorobenzylamine hydroiodide. Nano-Micro Lett. **16**(1), 189 (2024). <https://doi.org/10.1007/s40820-024-01406-4>
64. Z. Liu, Z. Xiong, S. Yang, K. Fan, L. Jiang et al., Strained heterojunction enables high-performance, fully textured perovskite/silicon tandem solar cells. Joule. (2024). https://doi.org/10.1016/j.joule.2024.06.015
65. D. Turkay, K. Artuk, X.-Y. Chin, D.A. Jacobs, S.-J. Moon et al., Synergetic substrate and additive engineering for over 30%-efficient perovskite-Si tandem solar cells. Joule. **8**(6), 1735-1753 (2024). <https://doi.org/10.1016/j.joule.2024.04.015>
66. E. Ugur, A.A. Said, P. Dally, S. Zhang, C.E. Petoukhoff et al., Enhanced cation interaction in perovskites for efficient tandem solar cells with silicon. Science. **385**(6708), 533-538 (2024). <https://doi.org/10.1126/science.adp1621>
67. Y. Chen, N. Yang, G. Zheng, F. Pei, W. Zhou et al., Nuclei engineering for even halide distribution in stable perovskite/silicon tandem solar cells. Science. **385**(6708), 554-560 (2024). <https://doi.org/10.1126/science.ado9104>
68. Z. Ying, X. Guo, H. Du, X. Li, M. Zhang et al., Hierarchical micro/nanostructured perovskite/silicon tandem solar cells with fully textured solution-processed conformal perovskite absorbers. ACS Energy Lett. **9**(8), 4018-4023 (2024). <https://doi.org/10.1021/acsenergylett.4c01594>
69. Z. Zhu, S. Yuan, K. Mao, H. Meng, F. Cai et al., Low-temperature atomic layer deposition of hole transport layers for enhanced performance and scalability in textured perovskite/silicon tandem solar cells. Adv Energy Mater. 2402365 (2024). <https://doi.org/10.1002/aenm.202402365>
70. F. Sahli, J. Werner, B.A. Kamino, M. Braeuninger, R. Monnard et al., Fully textured monolithic perovskite/silicon tandem solar cells with 25.2% power conversion efficiency. Nat Mater. **17**(9), 820 (2018). <https://doi.org/10.1038/s41563-018-0115-4>
71. B. Chen, Z.J. Yu, S. Manzoor, S. Wang, W. Weigand et al., Blade-coated perovskites on textured silicon for 26%-efficient monolithic perovskite/silicon tandem solar cells. Joule. **4**(4), 850-864 (2020). <https://doi.org/10.1016/j.joule.2020.01.008>
72. A. Al-Ashouri, E. Köhnen, B. Li, A. Magomedov, H. Hempel et al., Monolithic perovskite/silicon tandem solar cell with >29% efficiency by enhanced hole extraction. Science. **370**(6522), 1300-1309 (2020). <https://doi.org/10.1126/science.abd4016>
73. Y. Li, B. Shi, Q. Xu, L. Yan, N. Ren et al., Wide bandgap interface layer induced stabilized perovskite/silicon tandem solar cells with stability over ten thousand hours. Adv Energy Mater. **11**(48), 2102046 (2021). https://doi.org/10.1002/aenm.202102046
74. K. Liu, B. Chen, Z.J. Yu, Y. Wu, Z. Huang et al., Reducing sputter induced stress and damage for efficient perovskite/silicon tandem solar cells. J Mater Chem A. **10**(3), 1343-1349 (2022). <https://doi.org/10.1039/D1TA09143C>
75. R. Li, B. Chen, N. Ren, P. Wang, B. Shi et al., CsPbCl_3_-cluster-widened bandgap and inhibited phase segregation in a wide-bandgap perovskite and its application to niox-based perovskite/silicon tandem solar cells. Adv Mater. **34**(27), 2201451 (2022). https://doi.org/10.1002/adma.202201451
76. J. Liu, M. De Bastiani, E. Aydin, G.T. Harrison, Y. Gao et al., Efficient and stable perovskite-silicon tandem solar cells through contact displacement by MgF(x). Science. **377**(6603), 302-306 (2022). <https://doi.org/10.1126/science.abn8910>
77. N. Ren, C. Zhu, R. Li, S. Mazumdar, C. Sun et al., 50 °C low-temperature ALD SnO_2_ driven by H_2_O_2_ for efficient perovskite and perovskite/silicon tandem solar cells. Appl Phys Lett. **121**(3), 033502 (2022). <https://doi.org/10.1063/5.0091311>
78. X. Wang, Z. Ying, J. Zheng, X. Li, Z. Zhang et al., Long-chain anionic surfactants enabling stable perovskite/silicon tandems with greatly suppressed stress corrosion. Nat Commun. **14**(1), 2166 (2023). <https://doi.org/10.1038/s41467-023-37877-z>
79. Z. Ying, Z. Yang, J. Zheng, H. Wei, L. Chen et al., Monolithic perovskite/black-silicon tandems based on tunnel oxide passivated contacts. Joule. **6**(11), 2644-2661 (2022). https://doi.org/10.1016/j.joule.2022.09.006
80. X. Li, Z. Ying, J. Zheng, X. Wang, Y. Chen et al., Surface reconstruction for efficient and stable monolithic perovskite/silicon tandem solar cells with greatly suppressed residual strain. Adv Mater. **35**(30), e2211962 (2023). <https://doi.org/10.1002/adma.202211962>
81. J. Liu, Y.C. He, L. Ding, H. Zhang, Q.Y. Li et al., Perovskite/silicon tandem solar cells with bilayer interface passivation. Nature. (2024). <https://doi.org/10.1038/s41586-024-07997-7>
82. Y. Sun, L. Mao, T. Yang, H. Zhang, J. Shi et al., Ionic liquid modified polymer intermediate layer for improved charge extraction toward efficient and stable perovskite/silicon tandem solar cells. Small. **20**(21), 2308553 (2024). https://doi.org/10.1002/smll.202308553
83. X. Wang, J. Zheng, Z. Ying, X. Li, M. Zhang et al., Ultrathin (∼30 µm) flexible monolithic perovskite/silicon tandem solar cell. Science Bulletin. **69**(12), 1887-1894 (2024). https://doi.org/10.1016/j.scib.2024.04.022
84. T. Yang, L. Mao, J.H. Shi, P. Zeng, F.M. Li et al., Efficient and stable perovskite/silicon tandem solar cells modulated with triple-functional passivator. Adv Energy Mater. **14**(7), 2303149 (2024). <https://doi.org/10.1002/aenm.202303149>
85. Z. Ding, C. Kan, S. Jiang, M. Zhang, H. Zhang et al., Highly passivated TOPCon bottom cells for perovskite/silicon tandem solar cells. Nat Commun. **15**(1), 8453 (2024). <https://doi.org/10.1038/s41467-024-52309-2>
